# The burden of tuberculosis and drug resistance in 22 Sub-Saharan African countries, 1990–2021: a GBD 2021 analysis and progress towards WHO 2035 targets with projections to 2050

**DOI:** 10.3389/fmicb.2025.1695592

**Published:** 2025-11-17

**Authors:** Shirong Li, Emmanuel Mensah, Min Liu, Lingling Pan, Wei Lu, Susheng Zhou, Liqin Zhang, Yusheng Cheng, Hui Zhao, Shuoshuo Wei, Lei Zha

**Affiliations:** 1 Department of Respiratory and Critical Care Medicine, The Second Affiliated Hospital of Anhui Medical University, Hefei, Anhui, China; 2 Department of Pulmonary and Critical Care, Wuhu Hospital of East China Normal University (The Second People’s Hospital of Wuhu), Wuhu, Anhui, China; 3 Department of Pulmonary and Critical Care Medicine, The First Affiliated Hospital of Wannan Medical College (Yijishan Hospital of Wannan Medical College), Wuhu, Anhui, China; 4 Takoradi Hospital, Takoradi, Ghana; 5 Graduate School of Bengbu Medical University, Bengbu Medical University, Bengbu, Anhui, China; 6 Department of Cardiology, The First Affiliated Hospital of Wannan Medical College (Yijishan Hospital of Wannan Medical College), Wuhu, Anhui, China

**Keywords:** tuberculosis, Sub-Saharan Africa, drug-resistant tuberculosis, multidrug-resistant tuberculosis, extensively drug-resistant tuberculosis, antibiotic resistance, WHO End TB targets, epidemiology

## Abstract

**Background:**

Tuberculosis (TB) remains a major public health challenge in Sub-Saharan Africa (SSA), compounded by rising multidrug-resistant (MDR-TB), and extensively drug-resistant tuberculosis (XDR-TB) strains. This study aimed to quantify the burden, temporal trends, and subregional heterogeneity of TB across 22 selected SSA countries; project future trends to 2050; and evaluate the alignment of national TB policies with WHO End TB 2035 targets.

**Methods:**

We conducted a mixed-methods analysis using Global Burden of Disease (GBD) 2021 data. Age- and sex-specific TB incidence, prevalence, mortality, and disability-adjusted life years (DALYs) were analyzed across 22 SSA countries from 1990 to 2021. BAPC model projected disease burden to 2050. National TB policy alignment with WHO targets was assessed qualitatively. Drug-susceptible (DS-TB), MDR-TB, and XDR-TB forms were evaluated alongside key attributable risk factors.

**Results:**

Between 1990 and 2021, absolute TB incidence in SSA increased by 25.6% and prevalence by 44.2%, while mortality and DALYs declined by 14.0 and 24.8%, respectively. Age-standardized rates declined significantly across all metrics; incidence (−46.2%), prevalence (−35.2%), mortality (−56.4%), and DALYs (−60.9%). Progress varied substantially by region: Western SSA showed the greatest improvement, while Southern and Central SSA continued to face high burdens, with rising mortality in some areas. MDR-TB incidence surged by 743.2%, with XDR-TB also increasing markedly, particularly in Eastern and Central SSA. HIV co-infection amplified MDR/XDR-TB mortality, with Southern SSA most affected. Age- and sex-specific analyses revealed early-adulthood incidence peaks, male predominance in mortality and DALYs, and the highest MDR-TB burden among older adults. Leading risk factors for TB mortality included high alcohol use, elevated fasting plasma glucose, tobacco use, and high body mass index. Projections indicate SSA is unlikely to meet the WHO 2035 mortality reduction target, though Ghana, Guinea, and Tanzania are projected to achieve the incidence target.

**Conclusion:**

Despite overall declines in TB mortality, the growing DR-TB/MDR-TB/XDR-TB epidemic, significant subregional disparities, and systemic health system challenges threaten progress toward WHO End TB goals. Strengthening diagnostics, expanding treatment access, integrating care services, and addressing key metabolic and behavioral risk factors are essential to accelerate TB control efforts and align SSA with the 2035 targets.

## 1 Introduction

Tuberculosis (TB), caused by *Mycobacterium tuberculosis*, remains a major global health challenge, with Sub-Saharan Africa (SSA) bearing a disproportionately high burden of the disease (Shegaze et al., 2022; Ledesma et al., 2024). In 2023, TB caused 10.8 million new cases and 1.25 million deaths worldwide; SSA accounted for 24% of incident cases and over 33% of deaths despite representing only 14% of the global population (Naghavi et al., 2024; World Health Organization, 2024). TB is primarily transmitted through airborne droplets and may present as latent infection, carried asymptomatically by an estimated 25% of the global population, or progress to active disease, especially among immuno-compromised individuals, with symptoms including chronic cough, weight loss, night sweats, and fever (Liebenberg et al., 2022; Kapata et al., 2025; World Health Organization, 2025).

A pressing global threat is the increasing prevalence of drug-resistant TB (DR-TB), including multi-drug-resistant (MDR-TB) and extensively drug-resistant TB (XDR-TB), which significantly complicate treatment and increase mortality (Salam et al., 2023; Ahmed et al., 2024; Dean et al., 2022; Wang et al., 2025). MDR-TB is defined as infection with *M. tuberculosis* resistant to at least isoniazid and rifampicin, the two most potent first-line anti-TB drugs and XDR-TB refers to MDR-TB strains that are also resistant to any fluoroquinolone and at least one additional Group A drug, such as bedaquiline or linezolid, as defined by the World Health Organization (2024). In 2022, there were 410,000 MDR-TB cases globally, with 62,000 rifampicin-resistant cases reported in the African region alone, posing a major threat to achieving WHO End TB Strategy targets by 2035 (World Health Organization African Region, 2025). SSA is home to 16 of the 39 high-TB-burden countries, with Nigeria and the Democratic Republic of the Congo accounting for 4.6 and 3.1% of global cases, respectively (Atake, 2023; Yang et al., 2024). The region also carries 73% of the global TB-HIV co-infection burden, with high prevalence in countries such as South Africa, Nigeria, and Cameroon (Mahmud et al., 2011; Chanda-Kapata et al., 2022). MDR-TB rates vary considerably, from 1.5% in Ghana to 22.8% in Sudan, and reach nearly 100% among retreatment cases in Zimbabwe (Asante-Poku et al., 2015; Bedewi Omer et al., 2016; Affolabi et al., 2017; Chala and Usmael, 2020; Hajissa et al., 2021). Countries like Nigeria, Uganda, Zimbabwe, Malawi, Ethiopia, Burundi, and the Central African Republic account for two-thirds of SSA’s DR-TB burden, with primary transmission of resistant strains driving epidemics in settings such as South Africa and Benin (Dheda et al., 2017; Liyew et al., 2025). Additionally, DR-TB variation is pronounced, with SSA and South Asia bearing the highest global resistance burdens (Murray et al., 2022; Adewole et al., 2024).

Socioeconomic and health-system challenges further exacerbate the crisis. Treatment for MDR/XDR-TB is prolonged and costly, often exceeding the WHO catastrophic cost threshold, particularly in high-poverty regions such as Karamoja, Uganda, where TB treatment success rates are as low as 48% (Kerrigan et al., 2017; Megerso et al., 2020; Nidoi et al., 2021). Limited access to molecular diagnostics contributes to an estimated 2.7 million undiagnosed TB cases globally each year, with a significant proportion in SSA (Cattamanchi et al., 2015). The COVID-19 pandemic further disrupted TB control efforts, reducing case detection and treatment access and exposing critical health system fragilities (Chapman and Veras-Estévez, 2021; Mbewana Ntshanka and Msagati, 2023).

Despite these pressing challenges, comprehensive and long-term analyses of TB and DR-TB trends across SSA remain scarce, with existing studies often localized, lacking projections, or disconnected from policy frameworks (Ombura et al., 2016; Monde et al., 2021; Molla et al., 2022; Osei Sekyere et al., 2019). This study aims to address these gaps by leveraging the Global Burden of Disease (GBD) 2021 dataset to analyze TB prevalence, DR-TB, and policy effectiveness across 22 high-burden SSA countries. Our objectives are to (1) quantify historical and projected trends in TB and DR-TB strains from 1990 to 2050, (2) evaluate the implementation and challenges of national TB control policies, and (3) assess progress toward WHO End TB 2035 targets. This research integrates epidemiological modeling with policy analysis to create a robust evidence base, directly informing policymaking for strategic TB control and advancing progress toward elimination in SSA.

## 2 Methods

### 2.1 Study design

This mixed-methods study integrated quantitative epidemiological trend analysis and projections of *M. tuberculosis* (TB) burden with qualitative content analysis of national TB policy documents. The study assessed prevalence, antibiotic resistance, and risk factors in 22 Sub-Saharan African (SSA) countries across four subregions (Central, Eastern, Southern, Western) from 1990 to 2021, with projections to 2050, using data from the Global Burden of Disease (GBD) 2021 Study.

### 2.2 Data sources

#### 2.2.1 Epidemiological data

Data on TB, drug-susceptible TB (DS-TB), multidrug-resistant TB (MDR-TB) (1990–2021), and extensively drug-resistant TB (XDR-TB) (2000–2021) were sourced from the GBD 2021 Study via the Global Health Data Exchange.^1^ Metrics included prevalence, incidence, mortality, disability-adjusted life years (DALYs), and age-standardized rates (ASRs), disaggregated by age (all ages, age-standardized, 15–95 + years in 5-year bands), sex, year, and location (SSA, its subregions, and 22 countries selected).

Country selection criteria: Countries were selected based on (i) inclusion in the World Health Organization (WHO) list of High TB Burden Countries during at least one of the reference years between 2000 and 2023, and (ii) availability of data and national reports. Specifically, 12 of the selected countries are among the 18 African nations listed in WHO’s top 33 global high-TB-burden countries (World Health Organization, 2024). The remaining countries were included based on substantial population size, availability of national TB policy and surveillance data, and to ensure balanced representation across the four SSA subregions: Eastern, Western, Southern, and Central Africa. This approach ensured that the study captured both high-burden and data-rich contexts while maintaining geographic diversity. To complement the quantitative GBD estimates, a targeted narrative literature review (1990–2025) was conducted using PubMed and Google Scholar with the terms (“tuberculosis” OR “TB”) AND (“Sub-Saharan Africa” OR “Africa”) AND (“burden” OR “prevalence” OR “mortality” OR “DALYs” OR “risk factors” OR “antibiotic resistance” OR “health policy”).

#### 2.2.2 Policy data

National TB strategic plans, guidelines, and reports (2015–present, aligned with the WHO End TB Strategy) were collected from Ministries of Health, national TB program websites, WHO regional offices, and Global Fund archives using keywords (“National TB Strategic Plan,” “TB control policy,” “drug-resistant TB guidelines”) combined with country names. Documents from the 22 SSA countries were analyzed using qualitative content analysis, guided by a health systems framework adapted from prior studies (Xue et al., 2022), to evaluate alignment with WHO End TB Strategy targets, drug-resistant TB management, and risk factor mitigation.

### 2.3 Data analysis

#### 2.3.1 Temporal and age- and sex-specific trends

Age-standardized prevalence (ASPR), incidence (ASIR), mortality (ASMR), and DALY rates were analyzed for TB, DS-TB, MDR-TB (1990–2021), and XDR-TB (2000–2021) across SSA and its subregions. Trends were visualized using R (version 4.2.3) with packages tidyverse, ggplot2, readr, ggplot2, cowplot, plotly. The plot utilized geom_line to depict trends from 1990 to 2021, line plots with 95% UIs, color-coded by subregion (Central: blue, Eastern: orange, Southern: green, Western: red). For 2021, age- and sex-specific MDR-TB burden was assessed using bar plots for absolute numbers (with UI error bars) and line plots for rates per 100,000, scaled on a secondary y-axis.

#### 2.3.2 Statistical analysis

Age-standardized rates and absolute counts for deaths, prevalence, incidence, and DALYs were calculated. Percentage changes were computed as ((Value_2021 − Value_1990)/Value_1990) × 100 for TB, DS-TB, MDR-TB, and HIV/AIDS-co-infected cases, and ((Value_2021 − Value_2000)/Value_2000) × 100 for XDR-TB, with 95% UIs. Results were tabulated by region and metric.

#### 2.3.3 Risk factor attribution

Population attributable fractions (PAFs) for TB-related deaths were quantified for six risk factors (tobacco, high alcohol use, high body-mass index, high fasting plasma glucose, low physical activity, dietary risks) in SSA and its subregions for 1990 and 2021. PAFs were visualized using bar plots to compare temporal and regional contributions.

#### 2.3.4 Projections and WHO target assessment

The analysis aimed to assess progress toward the WHO End TB Strategy targets, a 90% reduction in incidence and a 95% reduction in mortality by 2035, using 2015 as a baseline, and to generate country-specific and region-specific projections through 2035 and 2050. Data were processed and analyzed in R (version 4.3.1), using packages including dplyr, ggplot2, forecast, and readr. For each country, we estimated percentage reductions in incidence and mortality between 2015 and 2021 and compared them with interpolated WHO target trajectories. Time series forecasting was performed using automated ARIMA models (auto.arima()), fitted to historical trends (1990–2021) and used to predict annual values to 2050. WHO targets were overlaid for comparison. Outputs were visualized using faceted line graphs, with observed estimates (1990–2021), ARIMA forecasts (2022–2050), and WHO targets distinguished with a red dashed line. Projections below zero were suppressed. A shaded region marked the 2015–2050 target period, and x-axis breaks were fixed at 2000, 2010, 2020, 2030, 2040, and 2050 for consistent readability. Progress toward WHO End TB Strategy targets (90% incidence and 95% mortality reduction by 2035, using 2015 as baseline) was assessed. Time series forecasting used automated ARIMA models (R package: forecast) fitted to historical trends (1990–2021) to predict annual values to 2050.

#### 2.3.5 Overall SSA TB projections (2022–2050)

Projections were generated using a Bayesian age-period-cohort (BAPC) model in JD_GBDR V2.31 software (Jingding Medical Technology Co., Ltd.), employing integrated nested Laplace approximations (INLA) for Bayesian inference, with sensitivity analyses assessing variations in intervention coverage and transmission dynamics. The model used ASIR, ASMR, ASPR, and DALYs (1990–2021 for DS-TB and MDR-TB; 2000–2021 for XDR-TB), incorporating historical trends, age, and cohort effects, adjusted for demographic shifts and healthcare access.

### 2.4 Software

Quantitative analyses were conducted using R (version 4.4.1), Microsoft Excel 2016, and JD_GBDR V2.31. Visualizations were generated with R (ggplot2, tidyverse) and Excel.

### 2.5 Ethical considerations

This study used publicly available, de-identified GBD 2021 data and official policy documents, requiring no institutional review board approval. GBD data use guidelines were followed.

## 3 Results

### 3.1 Overall burden of *Mycobacterium tuberculosis* in Sub-Saharan Africa (1990–2021)

Between 1990 and 2021, the burden of MTB in Sub-Saharan Africa (SSA) increased in terms of absolute incidence and prevalence but declined in mortality and disability-adjusted life years (DALYs). Incident cases rose from 1,756,526 (95% UI: 1,581,279–1,949,642) in 1990 to 2,206,541 (95% UI: 1,954,542–2,467,218) in 2021, representing a 25.62% increase. Prevalence increased from 178,447,050 (95% UI: 159,832,028–198,614,426) to 257,313,631 (95% UI: 229,669,367–289,126,065), a 44.20% rise. By contrast, mortality declined from 433,689 (95% UI: 366,247–518,433) to 373,075 (95% UI: 312,734–441,700), a 13.98% decrease. DALYs similarly fell from 22,778,559 (95% UI: 19,434,725–26,793,114) to 17,135,993 (95% UI: 14,014,437–20,397,541), marking a 24.77% reduction (95% UI: −27.89 to −23.87). Detailed estimates for number of deaths, incidence, prevalence and DALYs counts are provided in the Supplementary Tables S1–S4.

Age-standardized rates (ASRs) per 100,000 population declined steadily over the three decades. ASIR dropped from 478.07 (95% UI: 434.72–529.19) to 257.14 (95% UI: 229.14–284.70), a 46.21% reduction. ASPR fell from 40,175.02 (95% UI: 36,833.79–43,800.53) to 26,028.36 (95% UI: 23,494.71–28,695.01), a 35.21% decrease. Similarly, ASMR declined from 150.88 (95% UI: 124.80–182.81) to 65.83 (95% UI: 56.02–76.95), a 56.37% reduction. Age-standardized DALY rates (ASDR) decreased from 5,496.64 (95% UI: 4,671.66–6,492.05) to 2,146.64 (95% UI: 1,816.86–2,516.99), representing a 60.95% decline (Tables 1, 2). Detailed estimates for ASPR and ASDR are provided in the Supplementary Tables S5, S6.

**Table 1:** Age-standardized tuberculosis death rates and percentage change in Sub-Saharan Africa and sub-regions between 1990 and 2021 based on GBD 2021 report.

Region	TB			DS-TB			MDR-TB			XDR-TB		
	1990 ASR (95% UI)	2021 ASR (95% UI)	ASMR % change (1990–2021)	1990 ASR (95% UI)	2021 ASR (95% UI)	ASMR % change (1990–2021)	1990 ASR (95% UI)	2021 ASR (95% UI)	ASMR % change (1990–2021)	2000 ASR (95% UI)	2021 ASR (95% UI)	ASMR % change (2000–2021)
Sub-Saharan Africa	150.88 (124.80, 182.81)	65.83 (56.02, 76.95)	−56.37 (−55.11, −57.91)	150.28 (124.51, 182.25)	61.22 (51.50, 72.39)	−59.26 (−58.64, −60.28)	0.60 (0.23, 1.32)	4.55 (1.96, 9.25)	659.58 (762.22, 601.46)	0.03 (0.01, 0.06)	0.06 (0.02, 0.13)	136.27 (130.66, 127.24)
Western Sub-Saharan Africa	106.06 (89.09, 129.95)	45.01 (36.28, 54.17)	−57.56 (−59.28, −58.31)	105.46 (88.27, 129.44)	41.90 (33.04, 51.36)	−60.27 (−62.57, −60.32)	0.60 (0.19, 1.51)	3.07 (1.07, 6.62)	409.41 (451.45, 339.64)	0.02 (0.01, 0.05)	0.04 (0.01, 0.09)	112.78 (113.28, 81.16)
Eastern Sub-Saharan Africa	219.15 (177.37, 270.33)	81.60 (67.28, 98.09)	−62.76 (−62.07, −63.72)	218.76 (177.14, 270.02)	75.31 (61.01, 91.46)	−65.58 (−65.56, −66.13)	0.39 (0.12, 1.16)	6.21 (2.40, 12.52)	1504.74 (1970.43, 978.27)	0.04 (0.01, 0.08)	0.09 (0.03, 0.18)	131.34 (115.82, 121.49)
Central Sub-Saharan Africa	190.86 (126.63, 252.52)	102.62 (73.09, 148.92)	−46.24 (−42.28, −41.03)	189.68 (126.01, 250.06)	97.45 (68.51, 142.04)	−48.62 (−45.64, −43.20)	1.18 (0.23, 4.33)	5.10 (1.24, 17.43)	331.13 (441.39, 302.22)	0.03 (0.01, 0.09)	0.07 (0.02, 0.24)	160.96 (189.41, 178.40)
Southern Sub-Saharan Africa	82.08 (69.58, 100.72)	60.44 (53.28, 69.99)	−26.37 (−23.43, −30.51)	81.37 (69.14, 99.82)	55.85 (46.97, 65.85)	−31.37 (−32.06, −34.03)	0.71 (0.12, 2.21)	4.53 (1.65, 10.15)	540.02 (1247.86, 359.95)	0.02 (0.01, 0.05)	0.06 (0.02, 0.15)	200.59 (205.19, 183.39)

TB, tuberculosis; DS-TB, drug-susceptible tuberculosis; MDR-TB, multidrug-resistant tuberculosis; XDR-TB, extensively drug-resistant tuberculosis; ASMR, age-standardized mortality rate; ASPR, age-standardized prevalence rate; ASIR, age-standardized incidence rate (per 100,000 population) (95% UI) for all measures. Values represent age-standardized rates and percentage change (95% Uncertainty Interval) from 1990 to 2021 for TB, DS-TB, and MDR-TB, and from 2000 to 2021 for XDR-TB. NA indicates data not available.

**Table 2:** Age-standardized tuberculosis incidence rates and percentage change in Sub-Saharan Africa and sub-regions between 1990 and 2021 based on GBD 2021 report.

Region	TB			DS-TB			MDR-TB			XDR-TB		
	1990 ASR (95% UI)	2021 ASR (95% UI)	ASIR % change (1990–2021)	1990 ASR (95% UI)	2021 ASR (95% UI)	ASIR % change (1990–2021)	1990 ASR (95% UI)	2021 ASR (95% UI)	ASIR % change (1990–2021)	2000 ASR (95% UI)	2021 ASR (95% UI)	ASIR % change (2000–2021)
Sub-Saharan Africa	478.07 (434.72, 529.19)	257.14 (229.14, 284.70)	−46.21 (−47.29, −46.20)	476.96 (433.65, 527.76)	247.67 (221.72, 275.00)	−48.07 (−48.87, −47.89)	1.11 (0.65, 1.87)	9.38 (6.31, 13.61)	743.18 (863.98, 626.46)	0.04 (0.03, 0.06)	0.09 (0.06, 0.14)	152.14 (139.52, 143.89)
Western Sub-Saharan Africa	363.60 (330.80, 399.00)	177.75 (155.88, 200.88)	−51.11 (−52.88, −49.65)	362.50 (329.56, 397.83)	171.17 (149.53, 193.21)	−52.78 (−54.63, −51.44)	1.10 (0.58, 2.02)	6.52 (3.06, 13.88)	491.46 (431.04, 585.35)	0.03 (0.01, 0.06)	0.06 (0.03, 0.13)	120.62 (110.78, 120.33)
Eastern Sub-Saharan Africa	574.71 (515.67, 636.78)	282.94 (250.78, 314.99)	−50.77 (−51.37, −50.53)	574.13 (515.17, 636.19)	271.21 (240.14, 302.70)	−52.76 (−53.39, −52.42)	0.58 (0.25, 1.45)	11.61 (6.94, 19.19)	1898.18 (2695.10, 1221.87)	0.05 (0.03, 0.08)	0.12 (0.07, 0.19)	146.29 (127.30, 147.52)
Central Sub-Saharan Africa	545.66 (491.34, 602.20)	392.31 (352.37, 437.48)	−28.10 (−28.28, −27.35)	543.82 (489.79, 599.92)	382.38 (339.78, 425.41)	−29.69 (−30.63, −29.09)	1.84 (0.52, 6.11)	9.83 (2.91, 25.87)	433.55 (461.67, 323.15)	0.04 (0.01, 0.10)	0.10 (0.03, 0.26)	174.39 (159.85, 165.33)
Southern Sub-Saharan Africa	544.24 (490.50, 608.71)	417.09 (370.35, 470.40)	−23.36 (−24.49, −22.72)	542.03 (488.88, 607.38)	401.31 (352.70, 454.55)	−25.96 (−27.86, −25.16)	2.22 (0.48, 6.44)	15.65 (7.14, 32.63)	606.45 (1372.82, 406.50)	0.04 (0.02, 0.08)	0.13 (0.06, 0.29)	275.04 (253.59, 270.99)

TB, tuberculosis; DS-TB, drug-susceptible tuberculosis; MDR-TB, multidrug-resistant tuberculosis; XDR-TB, extensively drug-resistant tuberculosis; ASMR, age-standardized mortality rate; ASPR, age-standardized prevalence rate; ASIR, age-standardized incidence rate (per 100,000 population) (95% UI) for all measures. Values represent age-standardized rates and percentage change (95% Uncertainty Interval) from 1990 to 2021 for TB, DS-TB, and MDR-TB, and from 2000 to 2021 for XDR-TB. NA indicates data not available.

### 3.2 Country-level burden and trends of MTB and resistant forms across 22 Sub-Saharan African countries (1990–2021)

Over the study period, most of the 22 SSA countries recorded declines in *M. tuberculosis* burden, though patterns varied by country and tuberculosis (TB) subtype. Ethiopia, Sudan, and Zambia achieved the most pronounced reductions in ASMR and ASDR, with Ethiopia recording declines of −82.2% and −84.6%, respectively. By contrast, the Central African Republic consistently bore the highest burden with minimal improvement, while Zimbabwe was the only country where TB-related mortality and DALYs increased. Drug-susceptible TB (DS-TB) followed similar trends, with sharp declines in Ethiopia (ASMR: -83.4%; ASDR: −85.6%) and persistently high burdens in the Central African Republic and South Sudan. In contrast, multidrug-resistant TB (MDR-TB) rose markedly in most countries, particularly Uganda (+3727.2% ASMR), South Sudan, and Kenya. Côte d’Ivoire recorded the smallest increase and even experienced slight reductions in mortality and DALYs. Extensively drug-resistant TB (XDR-TB), assessed from 2000 to 2021, showed dramatic increases in Zimbabwe (ASMR: +863.9%; ASDR: +868.2%) and South Sudan, while Sudan and Zambia reported declining trends. Ghana and the Central African Republic experienced rising burdens of both MDR-TB and XDR-TB, underscoring persistent challenges in West and Central Africa despite some national progress (Tables 3, 4). Detailed estimates for ASPR and ASDR at the country levels are provided in the Supplementary Tables S7, S8.

**Table 3:** Age-standardized tuberculosis death rates and percentage change in 22 Sub-Saharan African countries between 1990 and 2021 based on GBD 2021 report.

Country	TB			DS-TB			MDR-TB			XDR-TB		
	1990 ASR (95% UI)	2021 ASR (95% UI)	ASMR % change (1990–2021)	1990 ASR (95% UI)	2021 ASR (95% UI)	ASMR % change (1990–2021)	1990 ASR (95% UI)	2021 ASR (95% UI)	ASMR % change (1990–2021)	2000 ASR (95% UI)	2021 ASR (95% UI)	ASMR % change (2000–2021)
Angola	205.06 (129.40, 286.11)	77.00 (53.18, 103.31)	−62.45 (−58.90, −63.89)	203.82 (128.98, 284.59)	72.67 (48.81, 99.41)	−64.35 (−62.16, −65.07)	1.24 (0.11, 5.18)	4.27 (0.56, 13.28)	244.02 (424.54, 156.11)	0.03 (0.00, 0.10)	0.06 (0.01, 0.20)	114.42 (147.31, 95.50)
Cameroon	81.98 (60.60, 103.43)	33.54 (19.74, 51.55)	−59.08 (−67.42, −50.16)	81.27 (59.87, 102.74)	31.78 (18.10, 49.07)	−60.90 (−69.77, −52.24)	0.71 (0.05, 3.46)	1.74 (0.23, 5.71)	146.73 (385.89, 65.20)	0.01 (0.00, 0.05)	0.02 (0.00, 0.09)	81.56 (120.11, 84.58)
Central African Republic	350.14 (224.32, 448.49)	264.97 (173.66, 356.79)	−24.32 (−22.58, −20.45)	347.88 (223.07, 444.84)	256.59 (168.59, 347.82)	−26.24 (−24.42, −21.81)	2.25 (0.29, 8.16)	8.26 (1.01, 29.93)	266.72 (250.44, 266.83)	0.05 (0.01, 0.13)	0.11 (0.01, 0.42)	151.63 (20.09, 226.07)
Chad	127.88 (93.83, 171.31)	81.83 (57.29, 107.40)	−36.01 (−38.95, −37.31)	126.87 (93.27, 170.69)	77.22 (54.27, 103.73)	−39.13 (−41.82, −39.23)	1.01 (0.08, 4.97)	4.55 (0.71, 14.15)	348.67 (760.49, 184.94)	0.03 (0.00, 0.09)	0.06 (0.01, 0.20)	146.25 (138.26, 115.24)
Côte d’Ivoire	90.87 (72.69, 111.06)	36.36 (25.66, 52.38)	−59.98 (−64.70, −52.84)	88.27 (69.38, 108.45)	33.74 (22.86, 49.64)	−61.78 (−67.06, −54.23)	2.59 (0.41, 8.63)	2.59 (0.38, 8.28)	−0.13 (−7.50, −4.08)	0.03 (0.01, 0.08)	0.04 (0.00, 0.12)	8.30 (−54.18, 53.39)
Democratic Republic of the Congo	180.84 (119.24, 259.09)	107.53 (71.83, 170.86)	−40.54 (−39.76, −34.06)	179.72 (118.50, 258.73)	101.98 (66.66, 163.44)	−43.26 (−43.74, −36.83)	1.12 (0.10, 5.45)	5.48 (0.77, 21.18)	389.22 (635.48, 288.91)	0.03 (0.00, 0.10)	0.08 (0.01, 0.30)	186.01 (225.36, 187.40)
Ethiopia	348.25 (272.82, 413.46)	62.00 (52.02, 74.10)	−82.20 (−80.93, −82.08)	347.73 (272.52, 412.93)	57.60 (44.97, 70.53)	−83.44 (−83.50, −82.92)	0.52 (0.05, 2.16)	4.33 (0.66, 14.02)	729.25 (1220.29, 548.22)	0.05 (0.01, 0.14)	0.06 (0.01, 0.19)	32.98 (−30.34, 36.20)
Ghana	105.44 (82.82, 136.51)	48.70 (35.51, 66.20)	−53.81 (−57.13, −51.50)	104.48 (81.65, 135.86)	45.88 (32.12, 62.66)	−56.09 (−60.66, −53.88)	0.95 (0.06, 4.21)	2.78 (0.33, 9.74)	192.47 (423.04, 131.31)	0.01 (0.00, 0.06)	0.04 (0.00, 0.15)	181.62 (243.32, 154.99)
Guinea	89.96 (73.20, 115.25)	47.34 (34.25, 67.03)	−47.38 (−53.20, −41.84)	89.18 (72.65, 114.00)	45.00 (31.64, 65.72)	−49.54 (−56.46, −42.35)	0.79 (0.05, 3.52)	2.31 (0.30, 8.26)	193.23 (474.74, 134.25)	0.01 (0.00, 0.05)	0.03 (0.00, 0.12)	127.18 (191.23, 142.88)
Kenya	140.56 (81.59, 225.15)	96.18 (56.53, 134.56)	−31.57 (−30.71, −40.23)	140.45 (81.53, 224.89)	92.39 (53.25, 128.39)	−34.22 (−34.69, −42.91)	0.11 (0.01, 0.52)	3.74 (0.65, 10.80)	3199.89 (7209.18, 1977.10)	0.02 (0.00, 0.08)	0.05 (0.01, 0.15)	151.02 (259.90, 92.99)
Madagascar	162.89 (125.76, 201.91)	82.98 (59.39, 118.68)	−49.06 (−52.78, −41.22)	162.72 (125.63, 201.79)	78.06 (55.85, 110.44)	−52.03 (−55.54, −45.27)	0.16 (0.02, 0.63)	4.85 (0.66, 17.30)	2849.50 (3747.86, 2636.47)	0.02 (0.00, 0.06)	0.07 (0.01, 0.26)	279.36 (170.21, 340.15)
Malawi	179.67 (131.16, 246.42)	82.63 (54.93, 119.19)	−54.01 (−58.12, −51.63)	179.52 (131.04, 245.80)	78.70 (51.81, 114.93)	−56.16 (−60.46, −53.24)	0.15 (0.01, 0.60)	3.88 (0.67, 12.56)	2528.87 (4832.99, 1997.03)	0.02 (0.00, 0.09)	0.05 (0.01, 0.18)	126.17 (151.37, 95.01)
Mozambique	223.53 (167.56, 308.52)	138.10 (96.69, 180.22)	−38.22 (−42.30, −41.59)	222.16 (166.69, 305.98)	122.27 (81.44, 166.56)	−44.96 (−51.14, −45.56)	1.38 (0.17, 5.32)	15.61 (3.55, 41.42)	1034.65 (1954.45, 678.15)	0.07 (0.03, 0.17)	0.22 (0.05, 0.56)	209.18 (77.51, 235.97)
Niger	147.72 (107.33, 210.20)	62.52 (40.83, 100.23)	−57.68 (−61.96, −52.32)	146.46 (106.56, 207.94)	59.19 (38.46, 95.00)	−59.59 (−63.90, −54.31)	1.26 (0.08, 6.01)	3.28 (0.42, 11.28)	160.85 (397.24, 87.71)	0.02 (0.00, 0.08)	0.05 (0.01, 0.15)	107.91 (183.81, 88.94)
Nigeria	110.82 (85.11, 148.38)	43.62 (32.37, 55.17)	−60.64 (−61.96, −62.82)	110.60 (85.07, 148.18)	39.97 (28.26, 50.26)	−63.86 (−66.78, −66.08)	0.23 (0.02, 1.02)	3.60 (0.74, 9.96)	1489.56 (3791.07, 875.81)	0.02 (0.00, 0.07)	0.05 (0.01, 0.14)	115.24 (164.53, 93.27)
South Africa	73.33 (61.40, 91.83)	45.88 (41.06, 52.78)	−37.44 (−33.13, −42.52)	72.56 (61.08, 90.72)	43.09 (36.46, 50.54)	−40.62 (−40.30, −44.29)	0.77 (0.11, 2.70)	2.75 (0.56, 7.36)	257.60 (424.41, 172.73)	0.02 (0.01, 0.06)	0.04 (0.01, 0.11)	83.96 (27.44, 82.90)
South Sudan	157.50 (104.04, 248.14)	114.05 (77.68, 181.17)	−27.59 (−25.33, −26.99)	157.19 (103.77, 247.20)	103.60 (66.50, 167.10)	−34.09 (−35.92, −32.40)	0.31 (0.02, 1.49)	10.30 (1.56, 33.45)	3219.00 (7056.27, 2145.37)	0.03 (0.00, 0.12)	0.14 (0.02, 0.49)	346.91 (584.63, 310.11)
Sudan	26.26 (16.59, 39.31)	5.87 (3.64, 8.96)	−77.63 (−78.07, −77.20)	26.15 (16.55, 39.10)	5.60 (3.40, 8.69)	−78.59 (−79.44, −77.79)	0.12 (0.01, 0.54)	0.26 (0.03, 0.94)	117.50 (231.44, 74.65)	0.02 (0.00, 0.07)	0.02 (0.00, 0.08)	−16.30 (−34.11, 7.93)
Uganda	134.02 (91.55, 221.44)	65.93 (50.51, 90.10)	−50.80 (−44.82, −59.31)	133.89 (91.44, 221.37)	60.95 (43.97, 83.49)	−54.48 (−51.92, −62.29)	0.13 (0.01, 0.65)	4.91 (0.92, 15.21)	3727.21 (9591.20, 2229.88)	0.03 (0.00, 0.10)	0.07 (0.01, 0.23)	128.08 (171.09, 124.43)
United Republic of Tanzania	132.09 (96.61, 185.61)	51.02 (35.19, 70.27)	−61.37 (−63.57, −62.14)	131.95 (96.56, 185.41)	48.07 (31.73, 65.99)	−63.57 (−67.14, −64.41)	0.15 (0.01, 0.71)	2.92 (0.43, 10.53)	1884.53 (3708.76, 1378.32)	0.02 (0.00, 0.05)	0.04 (0.01, 0.16)	159.65 (120.88, 213.09)
Zambia	184.64 (137.60, 226.14)	38.97 (23.96, 57.00)	−78.89 (−82.58, −74.79)	183.96 (137.33, 225.70)	35.98 (21.67, 54.16)	−80.44 (−84.22, −76.00)	0.68 (0.07, 2.46)	2.95 (0.41, 9.69)	332.71 (450.43, 294.56)	0.05 (0.01, 0.11)	0.04 (0.01, 0.13)	−9.12 (−64.44, 21.90)
Zimbabwe	97.42 (78.61, 116.60)	131.74 (84.00, 171.14)	35.24 (6.87, 46.78)	96.82 (77.95, 115.82)	119.74 (71.96, 158.50)	23.68 (−7.68, 36.86)	0.59 (0.04, 2.69)	11.83 (1.73, 37.85)	1891.18 (4343.88, 1308.32)	0.02 (0.00, 0.06)	0.16 (0.02, 0.55)	863.91 (1127.05, 787.15)

TB, tuberculosis; DS-TB, drug-susceptible tuberculosis; MDR-TB, multidrug-resistant tuberculosis; XDR-TB, extensively drug-resistant tuberculosis; ASMR, age-standardized mortality rate; ASPR, age-standardized prevalence rate; ASIR, age-standardized incidence rate; ASDR, age-standardized DALY rate (per 100,000 population) (95% UI). Values represent age-standardized rates and percentage change (95% Uncertainty Interval) from 1990 to 2021 for TB, DS-TB, and MDR-TB, and from 2000 to 2021 for XDR-TB. NA indicates data not available.

**Table 4:** Age-standardized tuberculosis incidence rates and percentage change table in 22 Sub-Saharan African countries between 1990 and 2021 based on GBD 2021 report.

Country	TB			DS-TB			MDR-TB			XDR-TB		
	1990 ASR (95% UI)	2021 ASR (95% UI)	ASIR % change (1990–2021)	1990 ASR (95% UI)	2021 ASR (95% UI)	ASIR % change (1990–2021)	1990 ASR (95% UI)	2021 ASR (95% UI)	ASIR % change (1990–2021)	2000 ASR (95% UI)	2021 ASR (95% UI)	ASIR % change (2000–2021)
Angola	536.03 (480.57, 594.85)	319.98 (285.66, 359.26)	−40.31 (−40.56, −39.60)	534.13 (478.83, 593.56)	311.20 (276.69, 351.58)	−41.74 (−42.21, −40.77)	1.90 (0.19, 7.22)	8.70 (1.35, 25.22)	356.82 (608.62, 249.43)	0.04 (0.00, 0.12)	0.08 (0.01, 0.24)	129.48 (159.40, 105.70)
Cameroon	229.75 (208.59, 253.48)	144.21 (124.72, 165.71)	−37.23 (−40.21, −34.63)	228.66 (206.22, 252.20)	140.59 (120.85, 161.84)	−38.51 (−41.40, −35.83)	1.10 (0.10, 4.85)	3.58 (0.62, 10.80)	227.03 (542.73, 122.62)	0.02 (0.00, 0.07)	0.03 (0.01, 0.10)	90.67 (159.90, 50.55)
Central African Republic	732.55 (661.75, 803.67)	595.99 (543.91, 649.65)	−18.64 (−17.81, −19.16)	729.46 (659.04, 801.48)	583.74 (528.65, 634.31)	−19.98 (−19.78, −20.86)	3.09 (0.58, 10.86)	12.10 (2.03, 40.09)	291.99 (247.20, 269.02)	0.06 (0.02, 0.13)	0.15 (0.02, 0.50)	155.65 (7.32, 299.26)
Chad	390.83 (354.17, 433.39)	237.88 (209.41, 269.42)	−39.13 (−40.87, −37.83)	389.22 (352.43, 432.03)	230.51 (199.85, 261.65)	−40.78 (−43.29, −39.44)	1.61 (0.16, 6.83)	7.29 (1.26, 20.71)	353.15 (686.20, 203.17)	0.03 (0.01, 0.11)	0.08 (0.01, 0.24)	152.93 (170.50, 107.97)
Côte d’Ivoire	356.05 (320.65, 395.58)	162.35 (141.14, 185.71)	−54.40 (−55.98, −53.05)	351.12 (314.60, 389.07)	156.73 (136.86, 180.57)	−55.36 (−56.50, −53.59)	4.93 (1.23, 14.56)	5.57 (1.06, 17.05)	12.99 (−13.95, 17.05)	0.04 (0.02, 0.09)	0.05 (0.01, 0.16)	14.55 (−52.92, 74.19)
Democratic Republic of the Congo	549.01 (494.13, 607.15)	422.62 (377.90, 470.73)	−23.02 (−23.52, −22.47)	547.22 (492.07, 605.86)	411.95 (363.21, 460.59)	−24.72 (−26.19, −23.98)	1.78 (0.22, 8.01)	10.56 (1.83, 32.78)	491.53 (720.33, 309.34)	0.04 (0.00, 0.13)	0.11 (0.02, 0.33)	199.24 (284.75, 160.71)
Ethiopia	750.77 (664.76, 848.07)	269.99 (241.85, 297.31)	−64.04 (−63.62, −64.94)	750.05 (664.37, 844.98)	260.29 (227.09, 290.16)	−65.30 (−65.82, −65.66)	0.71 (0.08, 2.97)	9.61 (1.44, 30.48)	1245.30 (1657.39, 927.63)	0.06 (0.02, 0.15)	0.09 (0.01, 0.28)	51.42 (−30.64, 81.88)
Ghana	364.94 (331.07, 399.04)	196.31 (170.76, 222.63)	−46.21 (−48.42, −44.21)	363.28 (330.77, 397.47)	190.67 (163.08, 218.24)	−47.51 (−50.70, −45.09)	1.67 (0.13, 6.85)	5.58 (0.86, 18.87)	234.93 (571.19, 175.51)	0.02 (0.00, 0.07)	0.05 (0.01, 0.18)	192.15 (266.60, 156.99)
Guinea	241.36 (221.01, 266.03)	167.30 (146.99, 190.39)	−30.68 (−33.49, −28.43)	240.19 (219.96, 264.47)	163.19 (141.79, 186.31)	−32.06 (−35.54, −29.55)	1.17 (0.10, 5.09)	4.07 (0.73, 12.04)	248.25 (620.90, 136.38)	0.02 (0.00, 0.07)	0.04 (0.01, 0.13)	132.55 (184.81, 96.38)
Kenya	394.22 (345.57, 445.23)	240.14 (210.34, 268.13)	−39.09 (−39.13, −39.78)	394.03 (345.53, 445.18)	234.06 (206.41, 262.62)	−40.60 (−40.26, −41.01)	0.19 (0.02, 0.76)	6.01 (1.43, 13.96)	3035.98 (6188.42, 1728.62)	0.03 (0.00, 0.10)	0.07 (0.02, 0.16)	136.52 (275.66, 63.47)
Madagascar	619.39 (563.09, 683.37)	302.77 (265.23, 346.41)	−51.12 (−52.90, −49.31)	619.08 (562.71, 683.07)	293.63 (255.81, 338.11)	−52.57 (−54.54, −50.50)	0.31 (0.04, 1.10)	9.04 (1.53, 29.32)	2837.12 (3989.61, 2559.10)	0.02 (0.01, 0.08)	0.09 (0.02, 0.30)	284.24 (164.35, 293.05)
Malawi	412.16 (358.65, 472.71)	243.44 (210.00, 279.76)	−40.94 (−41.45, −40.82)	411.96 (358.47, 472.29)	236.38 (201.59, 273.72)	−42.62 (−43.76, −42.04)	0.21 (0.03, 0.75)	6.98 (1.45, 20.66)	3255.35 (5408.53, 2657.94)	0.03 (0.01, 0.10)	0.08 (0.02, 0.22)	140.41 (181.94, 113.53)
Mozambique	639.81 (577.20, 717.92)	408.15 (357.97, 466.17)	−36.21 (−37.98, −35.07)	637.63 (575.42, 713.39)	381.94 (325.91, 434.51)	−40.10 (−43.36, −39.09)	2.18 (0.37, 7.21)	25.91 (6.93, 60.75)	1089.39 (1775.60, 742.70)	0.09 (0.05, 0.15)	0.30 (0.08, 0.71)	228.83 (52.49, 382.63)
Niger	432.23 (392.60, 474.21)	226.69 (198.70, 255.80)	−47.55 (−49.39, −46.06)	430.23 (391.83, 473.08)	220.60 (192.60, 250.39)	−48.73 (−50.84, −47.07)	2.00 (0.17, 9.10)	6.04 (1.00, 17.00)	202.02 (476.51, 86.76)	0.03 (0.00, 0.11)	0.06 (0.01, 0.17)	111.10 (167.59, 55.61)
Nigeria	389.24 (350.16, 429.86)	178.16 (156.26, 201.19)	−54.23 (−55.37, −53.20)	388.82 (349.03, 429.21)	169.84 (147.37, 191.85)	−56.32 (−57.78, −55.30)	0.42 (0.04, 1.67)	8.24 (2.22, 23.87)	1851.76 (5159.40, 1325.17)	0.03 (0.01, 0.09)	0.08 (0.02, 0.22)	125.87 (180.03, 136.39)
South Africa	561.56 (500.16, 625.10)	458.05 (403.15, 517.17)	−18.43 (−19.40, −17.27)	558.98 (496.36, 623.32)	444.05 (389.73, 503.79)	−20.56 (−21.48, −19.18)	2.58 (0.45, 8.29)	13.90 (3.88, 35.30)	438.95 (756.91, 325.84)	0.04 (0.02, 0.09)	0.11 (0.03, 0.27)	186.61 (90.01, 183.21)
South Sudan	593.10 (533.65, 660.77)	433.45 (381.25, 493.80)	−26.92 (−28.56, −25.27)	592.52 (533.33, 660.18)	412.45 (351.93, 476.88)	−30.39 (−34.01, −27.77)	0.58 (0.06, 2.59)	20.80 (4.07, 61.69)	3515.75 (7139.95, 2278.53)	0.04 (0.01, 0.18)	0.20 (0.04, 0.59)	355.99 (583.75, 237.10)
Sudan	122.82 (111.00, 136.40)	47.70 (41.70, 54.47)	−61.16 (−62.43, −60.07)	122.57 (110.81, 136.35)	46.66 (40.08, 53.48)	−61.93 (−63.83, −60.78)	0.25 (0.03, 1.09)	1.00 (0.13, 3.90)	305.05 (391.98, 258.88)	0.04 (0.01, 0.09)	0.04 (0.01, 0.15)	6.87 (−17.05, 68.24)
Uganda	385.96 (351.74, 420.12)	236.11 (214.63, 258.33)	−38.83 (−38.98, −38.51)	385.76 (351.64, 419.92)	226.76 (202.52, 250.89)	−41.22 (−42.41, −40.25)	0.20 (0.02, 0.87)	9.25 (2.29, 24.02)	4483.50 (12005.35, 2670.85)	0.04 (0.01, 0.11)	0.10 (0.02, 0.26)	150.57 (232.64, 130.40)
United Republic of Tanzania	458.80 (400.32, 522.39)	188.73 (163.23, 215.76)	−58.87 (−59.23, −58.70)	458.55 (400.15, 521.68)	183.20 (155.93, 209.73)	−60.05 (−61.03, −59.80)	0.25 (0.03, 1.06)	5.47 (0.94, 19.37)	2064.22 (3252.49, 1734.14)	0.02 (0.01, 0.06)	0.06 (0.01, 0.21)	156.81 (95.85, 236.32)
Zambia	399.04 (356.43, 441.21)	236.89 (202.27, 271.77)	−40.64 (−43.25, −38.40)	398.10 (356.06, 439.95)	227.53 (189.83, 262.26)	−42.85 (−46.69, −40.39)	0.94 (0.13, 3.06)	9.28 (1.58, 28.40)	885.42 (1070.34, 826.89)	0.06 (0.03, 0.12)	0.08 (0.01, 0.24)	30.22 (−54.22, 104.46)
Zimbabwe	481.38 (410.42, 568.37)	322.85 (277.20, 373.60)	−32.93 (−32.46, −34.27)	479.95 (409.42, 566.88)	301.81 (250.04, 353.58)	−37.12 (−38.93, −37.63)	1.44 (0.13, 6.59)	20.80 (3.23, 69.82)	1347.63 (2435.92, 959.58)	0.03 (0.00, 0.09)	0.23 (0.04, 0.80)	796.61 (808.89, 780.85)

TB, tuberculosis; DS-TB, drug-susceptible tuberculosis; MDR-TB, multidrug-resistant tuberculosis; XDR-TB, extensively drug-resistant tuberculosis; ASMR, age-standardized mortality rate; ASPR, age-standardized prevalence rate; ASIR, age-standardized incidence rate; ASDR, age-standardized DALY rate (per 100,000 population) (95% UI). Values represent age-standardized rates and percentage change (95% Uncertainty Interval) from 1990 to 2021 for TB, DS-TB, and MDR-TB, and from 2000 to 2021 for XDR-TB. NA indicates data not available.

### 3.3 Rising burden of drug-resistant tuberculosis and subregional patterns in SSA (1990–2021)

Between 1990 and 2021, age-standardized DS-TB remained the most prevalent form of TB in SSA; however, MDR-TB and XDR-TB exhibited alarming growth. While DS-TB ASIR fell by 48.1% and DS-TB ASMR by 59.3%, MDR-TB ASIR surged by 743.2% and MDR-TB ASMR by 659.6%, with the sharpest rise in Eastern SSA (ASIR: +1898.2%). Although less common, XDR-TB increased markedly between 2000 and 2021 in SSA, with a 136.3% rise in ASMR and a 118.9% increase in ASDRs, particularly in Central and Southern SSA (Tables 1, 2). Detailed estimates for ASPR and ASDR are provided in the Supplementary Tables S5, S6.

Subregional trends revealed divergent trajectories in absolute numbers. Western and Eastern SSA reduced TB ASMR by −13.2% and −27.5%, respectively, while Central and Southern SSA recorded increases of +9.6% and +32.8%. TB DALYs followed similar patterns, declining in Western (−20.1%) and Eastern (−37.7%) SSA but rising in Southern SSA (+11.5%) (Table 1). Although age-standardized DS-TB rates declined consistently across all subregions, absolute DS-TB incidence increased by up to +68.6%, particularly in Central SSA (Table 1). Conversely, age-standardized MDR-TB and XDR-TB burdens increased across all indicators and regions, with MDR-TB ASDR increasing by as much as +1344.4% in Eastern SSA and XDR-TB ASIR by up to +275.0% in Southern SSA, underscoring the escalating threat of antibiotic resistance (Tables 1, 2).

### 3.4 HIV–tuberculosis co-epidemic in Sub-Saharan Africa

Between 1990 and 2021, the HIV–TB co-epidemic exhibited divergent trends. The ASMR for HIV/AIDS–DS-TB decreased by 25.44%, whereas HIV/AIDS–MDR-TB mortality increased sharply by 1391.66%. Southern SSA was the only subregion to record rising mortality for HIV/AIDS–DS-TB (+205.35%) alongside the most dramatic increase in HIV/AIDS–MDR-TB mortality (+3630.77%). Eastern SSA also recorded a marked increase in HIV/AIDS–MDR-TB mortality (+2719.63%). While HIV/AIDS–DS-TB incidence declined across SSA by 17.90%, HIV/AIDS–MDR-TB incidence increased by 1247.21%. From 2000 to 2021, the burden of HIV/AIDS–XDR-TB also rose, with ASMR increasing by 13.11% across SSA and by 61.50% in Southern SSA. These findings highlight a worsening shift towards drug-resistant TB among HIV co-infected populations (Table 5). Detailed estimates for ASMR, ASIR, ASPR, ASDR are provided in the Supplementary Tables S9–S11.

**Table 5:** Age-standardized HIV/AIDS-tuberculosis death rates and percentage change between 1990 and 2021 in SSA and subregions.

Region	TB			HIV/AIDS-DS-TB			HIV/AIDS-MDR-TB			HIV/AIDS- XDR-TB		
	1990 ASR (95% UI)	2021 ASR (95% UI)	ASMR % change (1990–2021)	1990 ASR (95% UI)	2021 ASR (95% UI)	ASMR % change (1990–2021)	1990 ASR (95% UI)	2021 ASR (95% UI)	ASMR % change (1990–2021)	2000 ASR (95% UI)	2021 ASR (95% UI)	ASMR % change (2000–2021)
Sub-Saharan Africa	150.88 (124.80, 182.81)	65.83 (56.02, 76.95)	−56.37 (−55.11, −57.91)	24.20 (16.49, 34.19)	18.05 (14.19, 21.75)	−25.44 (−13.95, −36.37)	0.11 (0.03, 0.26)	1.61 (0.69, 3.08)	1391.66 (1961.84, 1098.73)	0.02 (0.01, 0.04)	0.02 (0.01, 0.04)	13.11 (11.89, 6.21)
Western Sub-Saharan Africa	106.06 (89.09, 129.95)	45.01 (36.28, 54.17)	−57.56 (−59.28, −58.31)	8.73 (6.36, 12.04)	7.84 (5.18, 11.29)	−10.21 (−18.57, −6.23)	0.12 (0.03, 0.34)	0.67 (0.21, 1.58)	445.13 (617.95, 367.47)	0.01 (0.00, 0.02)	0.01 (0.00, 0.02)	5.08 (−0.50, 3.71)
Eastern Sub-Saharan Africa	219.15 (177.37, 270.33)	81.60 (67.28, 98.09)	−62.76 (−62.07, −63.72)	43.32 (29.00, 61.92)	19.59 (14.85, 24.34)	−54.77 (−48.80, −60.68)	0.06 (0.01, 0.19)	1.81 (0.74, 3.72)	2719.63 (4910.02, 1845.06)	0.02 (0.01, 0.05)	0.03 (0.01, 0.05)	1.36 (8.30, 0.67)
Central Sub-Saharan Africa	190.86 (126.63, 252.52)	102.62 (73.09, 148.92)	−46.24 (−42.28, −41.03)	19.22 (12.94, 28.43)	8.92 (6.62, 11.85)	−53.59 (−48.87, −58.34)	0.14 (0.02, 0.54)	0.53 (0.16, 1.27)	277.92 (693.34, 133.40)	0.01 (0.00, 0.03)	0.01 (0.00, 0.02)	−16.65 (−6.57, −23.83)
Southern Sub-Saharan Africa	82.08 (69.58, 100.72)	60.44 (53.28, 69.99)	−26.37 (−23.43, −30.51)	21.77 (15.07, 32.23)	66.48 (55.76, 73.54)	205.35 (270.02, 128.19)	0.16 (0.02, 0.62)	6.13 (2.27, 13.47)	3630.77 (10347.53, 2065.60)	0.05 (0.02, 0.14)	0.09 (0.03, 0.21)	61.50 (70.48, 47.51)

TB, tuberculosis; HIV/AIDS-DS-TB, HIV/AIDS - drug-susceptible tuberculosis; HIV/AIDS-MDR-TB, HIV/AIDS - multidrug-resistant tuberculosis; HIV/AIDS-XDR-TB, HIV/AIDS - extensively drug-resistant tuberculosis; ASMR, age-standardized mortality rate; ASPR, age-standardized prevalence rate; ASIR, age-standardized incidence rate (per 100,000 population) (95% UI) for all measures. Values represent age-standardized rates and percentage change (95% Uncertainty Interval) from 1990 to 2021 for TB, DS-TB, and MDR-TB, and from 2000 to 2021 for XDR-TB. NA indicates data not available.

### 3.5 Attributable risk factors for *Mycobacterium tuberculosis*, DS-TB, and MDR/XDR-TB mortality

**Figure 1:**
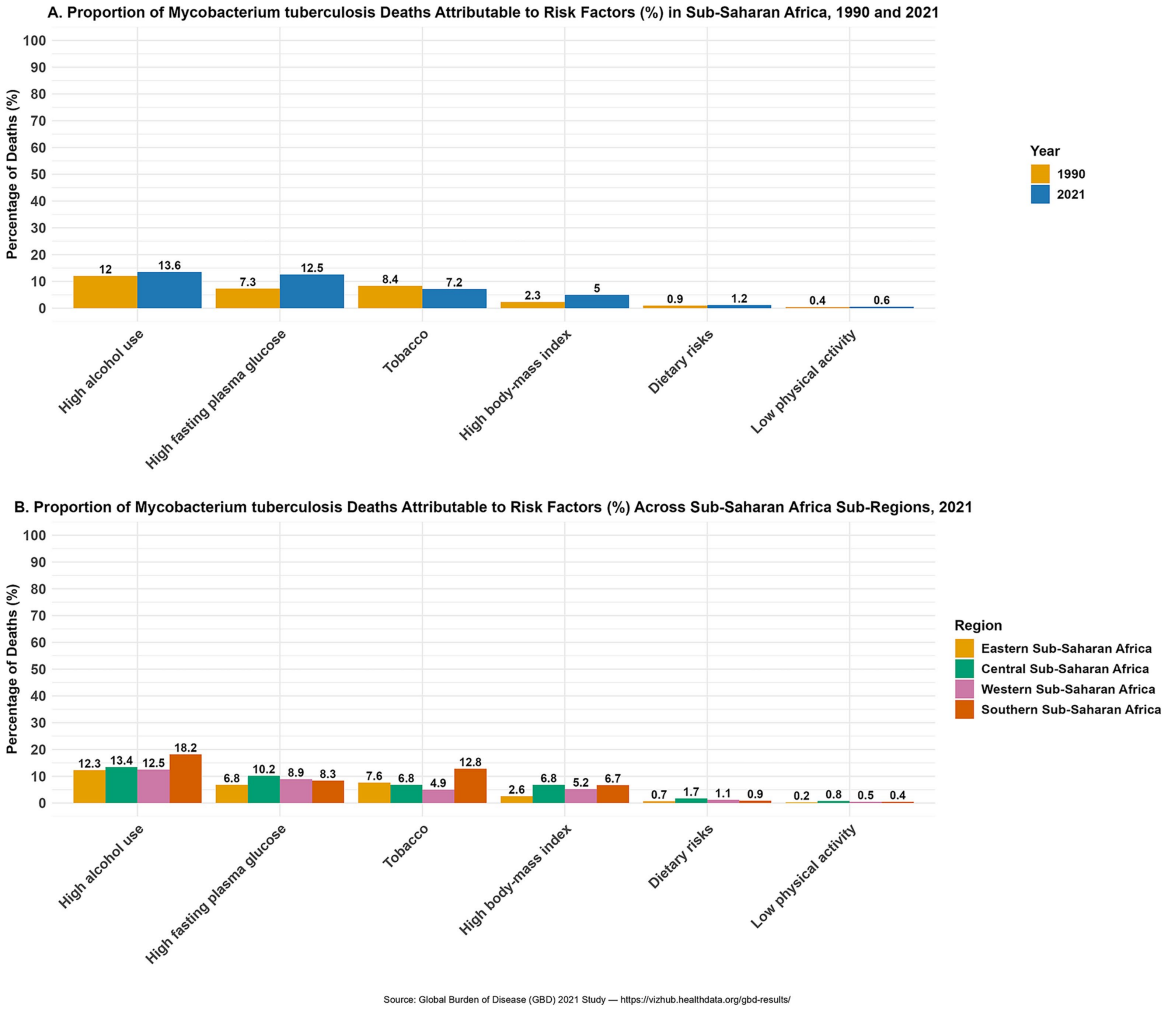
Proportion of *Mycobacterium tuberculosis* deaths attributable to risk factors in Sub-Saharan Africa, 1990 and 2021. The percentage of tuberculosis deaths attributable to risk factors, including high alcohol use, high fasting plasma glucose, tobacco use, high body-mass index, dietary risks, and low physical activity. **(A)** Comparison of attributable proportions for the entire region in 1990 (yellow) and 2021 (blue) using stacked bar charts. **(B)** Distribution of attributable risk factors across four sub-regions in 2021: Eastern (yellow), Central (green), Western (pink), and Southern (red) Sub-Saharan Africa. Source: Global Burden of Disease (GBD) 2021 Study.

Population attributable fraction (PAF) analysis revealed notable shifts in the contribution of risk factors to TB-related deaths and DALYs between 1990 and 2021. In 2021, the leading risk factors were high alcohol use (13.55% of deaths; 13.23% of DALYs), high fasting plasma glucose (12.56%; 8.23%), tobacco use (7.20%; 7.43%), high body mass index (4.95%; 4.56%), dietary risks (1.21%; 1.02%), and low physical activity (0.56%; 0.42%). High alcohol use remained the leading contributor to TB mortality and DALYs over the three decades, with a slight proportional increase by 2021. In 1990, tobacco was the second-leading contributor, but by 2021 it was overtaken by high fasting plasma glucose. High body mass index consistently ranked fourth but nearly doubled its attributable proportion over the period. Dietary risks and low physical activity remained the least influential risk factors (Figure 1). DALYs proportions are shown in Supplementary Figures S1a,b.

In 2021, Southern SSA recorded the highest proportion of TB-related deaths attributable to high alcohol use (19.00%), tobacco use (14.07%), and high body mass index (7.74%). Similar patterns were observed for DALYs, although Southern SSA ranked second in DALYs attributable to high body mass index (6.69%). Central Africa had the highest proportion of TB deaths attributable to high fasting plasma glucose (14.54%) and the largest DALY burden due to high body mass index (6.79%), as well as the highest contributions from dietary risks (1.90%) and low physical activity (1.01%). Western SSA reported the lowest contributions from tobacco (4.64% for deaths; 4.94% for DALYs) but notable contributions from high fasting plasma glucose (13.74%; 8.86%) and dietary risks (1.39%; 1.14%). Eastern SSA consistently recorded the lowest burden for most risk factors, except tobacco, for which it ranked second (7.40% of deaths; 7.62% of DALYs) (Figure 1). DALYs proportions are shown in Supplementary Figures S1a,b.

### 3.6 Age- and sex-specific burden of tuberculosis and multidrug-resistant tuberculosis in Sub-Saharan Africa, 2021 (absolute counts and rates)

**Figure 2:**
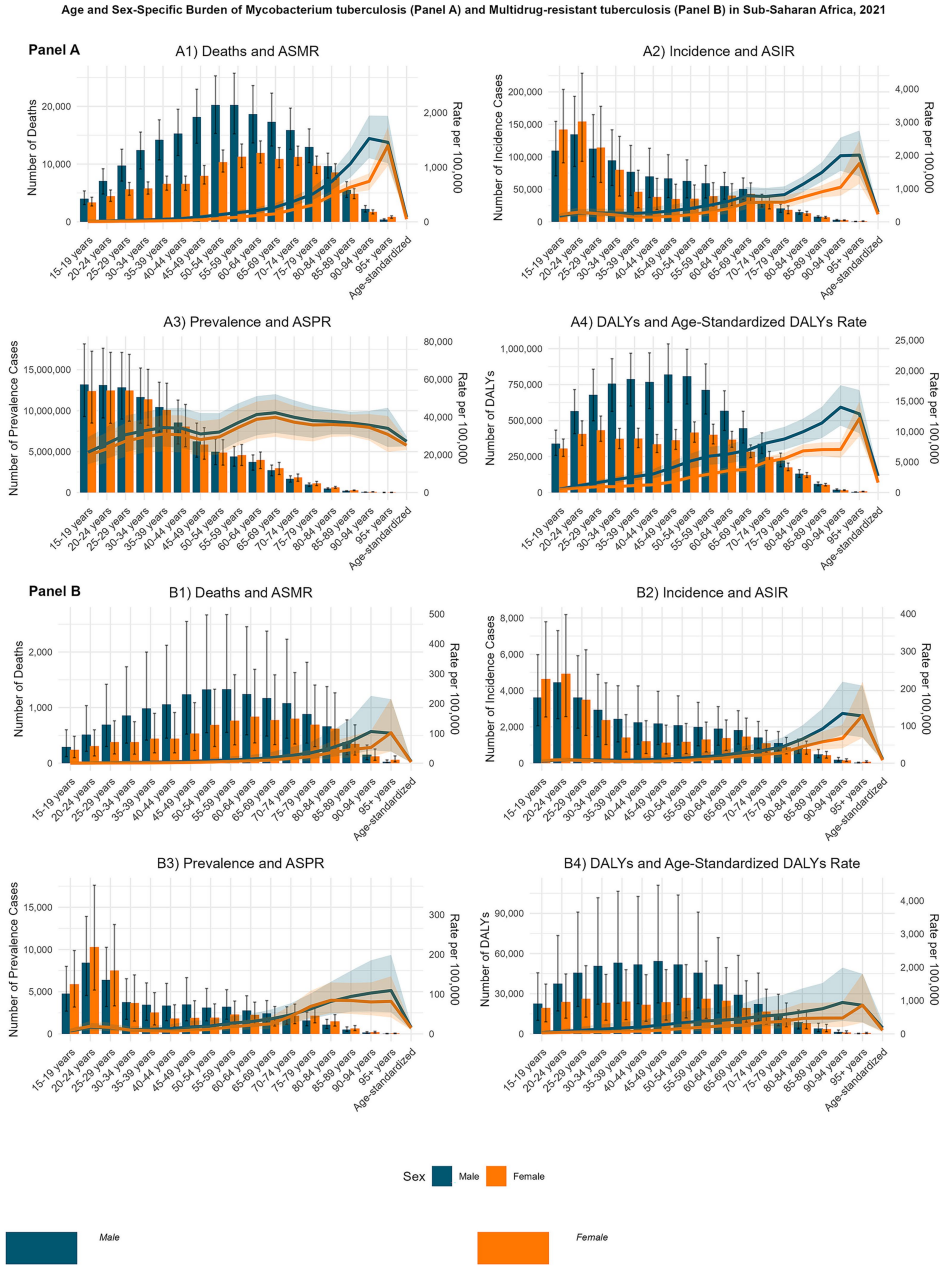
Age- and sex-specific burden of *Mycobacterium tuberculosis* (MTB) and multidrug-resistant tuberculosis (MDR-TB) in Sub-Saharan Africa, 2021. Bar plots represent absolute numbers (with uncertainty interval (UI) error bars), and line plots represent rates per 100,000 population (scaled on a secondary y-axis). Panel **(A)**: MTB; **(A1)** Age-standardized mortality rate (ASMR) of MTB; **(A2)** Age-standardized incidence rate (ASIR) of MTB; **(A3)** Age-standardized prevalence rate (ASPR) of MTB; **(A4)** Age-standardized disability-adjusted life years (DALYs) rate of MTB. Panel **(B)**: MDR-TB: **(B1)**: Age-standardized mortality rate (ASMR) of MDR-TB; **(B2)** Age-standardized incidence rate (ASIR) of MDR-TB; **(B3)** Age-standardized prevalence rate (ASPR) of MDR-TB; **(B4)** Age-standardized disability-adjusted life years (DALYs) rate of MDR-TB.

In 2021, *M. tuberculosis* (MTB)-related mortality in SSA demonstrated distinct age- and sex-specific patterns. Among males, the highest absolute number of deaths occurred in the 55–59-year age group (approximately 20,240.50 deaths), whereas among females, mortality peaked in the 60–64-year age group (approximately 11,944.50 deaths). Mortality rates per 100,000 population increased markedly with age, with the highest recorded in males aged 90–94 years (approximately 1,526.77) and females aged ≥95 years (approximately 1,394.71), indicating a strong age-related gradient (Figure 2A1). For multidrug-resistant tuberculosis (MDR-TB), male mortality also peaked in the 55–59-year age group, while female mortality peaked in the 60–64-year group. Mortality rates rose with age, reaching approximately 106.19 per 100,000 among males aged 90–94 years and 102.20 among females aged ≥95 years (Figure 2B1).

MTB incidence showed a pronounced early-adulthood peak, particularly among females. The highest absolute number of new cases was observed in the 20–24-year age group, with approximately 154,526.21 cases in females and 134,680.46 in males. However, incidence rates per 100,000 population were highest in the oldest age groups, peaking at approximately 2,007.65 among males aged ≥95 years and 1,755.02 among females aged ≥95 years (Figure 2A2). MDR-TB incidence peaked in early adulthood but, similar to mortality, rates escalated with age, reaching 133.27 per 100,000 among males aged 90–94 years and 128.16 among females aged ≥95 years (Figure 2B2).

Prevalence estimates in 2021 revealed a narrow gender gap across most age groups. The highest absolute MTB prevalence occurred in the 15–19-year age group, with approximately 13,209,864.33 cases in males and 12,476,488.54 in females. The highest prevalence rates were recorded in the 65–69-year age group for both sexes, 42,303.21 per 100,000 in males and 39,857.74 in females, suggesting a balanced distribution (Figure 2A3). MDR-TB prevalence exhibited a gender shift across the lifespan, with younger females experiencing higher burdens and older males predominating in later years. Prevalence rates increased with age, peaking at 109.18 per 100,000 in males aged 90–94 years and 84.66 per 100,000 in females aged 80–84 years (Figure 2B3).

DALY patterns indicated a male predominance in MTB-related disability and premature mortality. Peak absolute DALYs occurred among males aged 45–49 years (approximately 820,263.77) and females aged 25–29 years (approximately 433,851.94). DALY rates per 100,000 were highest in males aged 90–94 years (14,022.09) and females aged ≥95 years (12,138.72) (Figure 2A4). For MDR-TB, DALY patterns revealed substantial age- and sex-specific disparities. Peak absolute DALYs occurred in males aged 45–49 years and females aged 50–54 years. DALY rates per 100,000 increased with age, reaching 943.12 in males aged 90–94 years and 862.81 in females aged ≥95 years (Figure 2B4).

### 3.7 Tuberculosis mortality and incidence temporal trends in SSA and subregions, 1990–2021

**Figure 3:**
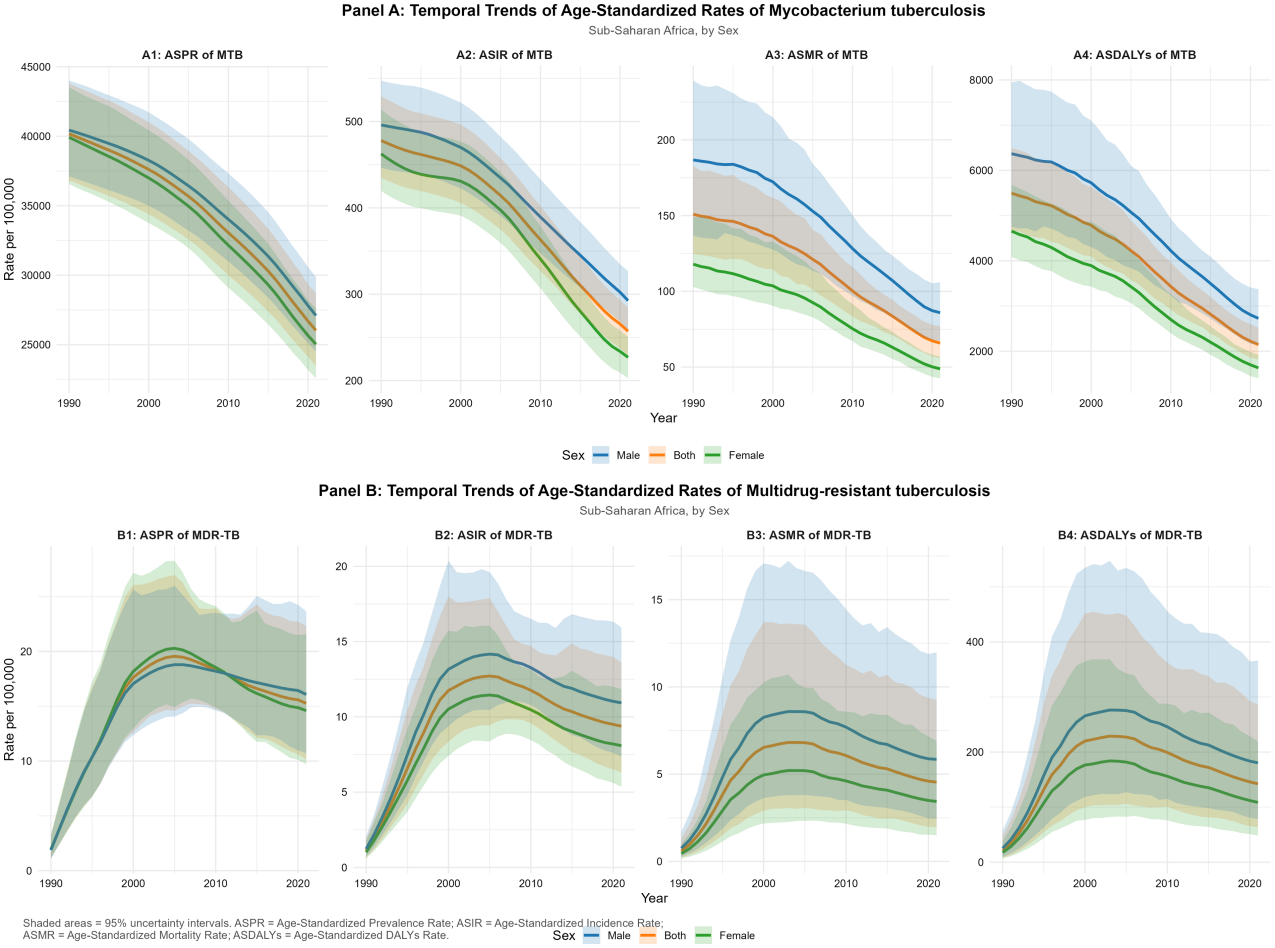
Temporal trends in the burden of *Mycobacterium tuberculosis* (MTB) and multidrug-resistant tuberculosis (MDR-TB) in Sub-Saharan Africa (SSA), 1990–2021. Line plots show trends with 95% uncertainty intervals (UIs) for males (blue), females (green), and the total SSA population (orange).Panel **(A)**: Temporal Trends of Age−Standardized Rates of Mycobacterium tuberculosis: **(A1)** ASPR of MTB; **(A2)** ASIR of MTB; **(A3)** ASMR of MTB; **(A4)** ASDALYs of MTB. Panel **(B)**: Temporal Trends of Age−Standardized Rates of Multidrug−resistant tuberculosis: **(B1)** ASPR of MDR−TB; **(B2)** ASIR of MDR−TB; **(B3)** ASMR of MDR−TB; **(B4)** ASDALYs of MDR−TB.

From 1990 to 2021, SSA experienced sustained declines in ASIR and ASMR for *M. tuberculosis*. However, post-2020 trends reveal a slight uptick in ASMR and ASPR (Figure 3A). For MDR-TB and XDR-TB ASPR, ASIR, ASMR, and ASDR in SSA increased steadily across the study period for both sexes, with males consistently showing the highest burden (Figure 3B; Supplementary Figure S3d). MDR-TB rates peaked between 2000 and 2010 before entering a gradual decline, whereas XDR-TB rates rose until the mid-2000s and have since plateaued or shown slight decreases by 2021 (Supplementary Figure S3d).

**Figure 4:**
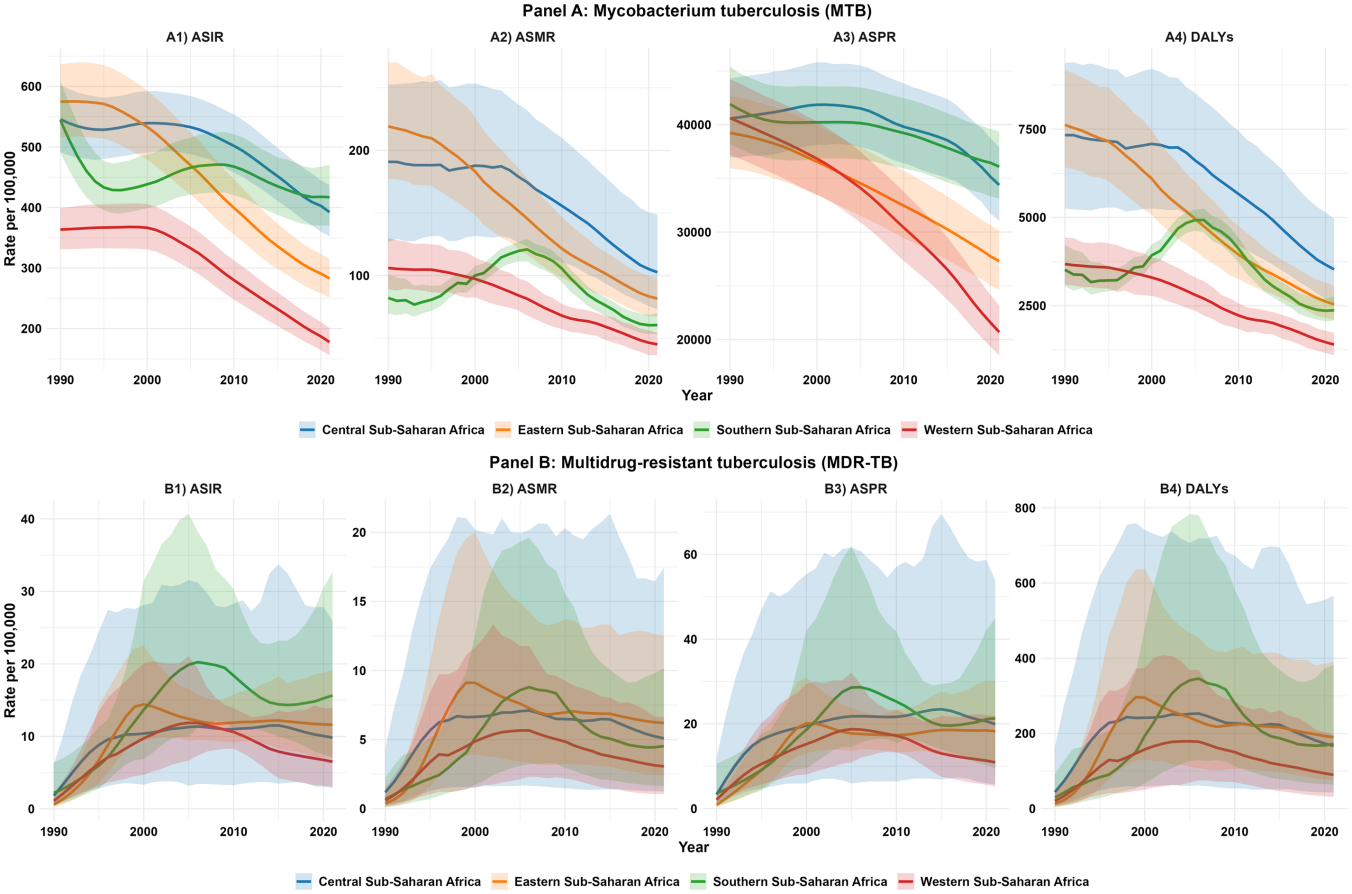
Sub-regional temporal trends in the burden of *Mycobacterium tuberculosis* (MTB) and multidrug-resistant tuberculosis (MDR-TB) in Sub-Saharan Africa, 1990–2021. Line plots show trends with 95% uncertainty intervals (UIs), color-coded by sub-region: Central (blue), Eastern (orange), Southern (green), and Western (red) Sub-Saharan Africa. Panel **(A)**: Mycobacterium tuberculosis (MTB): **(A1)** Age-standardized incidence rate (ASIR) of MTB; **(A2)** Age-standardized mortality rate (ASMR) of MTB; **(A3)** Age-standardized prevalence rate (ASPR) of MTB; **(A4)** Age-standardized disability-adjusted life years (DALYs) rate of MTB. Panel **(B)**: Multidrug−resistant tuberculosis (MDR−TB): **(B1)** Age-standardized incidence rate (ASIR) of MDR−TB; **(B2)** Age-standardized mortality rate (ASMR) of MDR−TB; **(B3)** Age-standardized prevalence rate (ASPR) of MDR−TB; **(B4)** Age-standardized disability-adjusted life years (DALYs) rate of MDR−TB.

Analysis of the ASMR and ASIR for MTB across SSA sub-regions revealed overall declines from 1990 to 2021, though patterns varied (Figure 4A) Southern SSA’s ASMR declined from 82.1 per 100,000 in 1990 to 60.4 in 2021, with a pronounced peak at 120.9 in 2005, coinciding with the height of the HIV/AIDS epidemic. Eastern SSA’s ASMR decreased from 219.2 to 81.6 per 100,000, reflecting substantial public health gains. Central SSA recorded a reduction from 190.9 to 102.6 per 100,000, though trends were relatively stable compared with other regions. Western SSA achieved the most consistent decline, from 106.1 to 45.0 per 100,000. For incidence, Southern SSA declined from 544.2 per 100,000 in 1990 to 417.1 in 2021, with a peak at 468.7 in 2006. Eastern SSA fell from 574.7 to 282.9 per 100,000, and Central SSA from 545.7 to 392.3 per 100,000. Western SSA achieved the steepest decline, from 363.6 to 177.8 per 100,000 (Figure 4A). Overall, SSA demonstrated sustained reductions in both mortality and incidence, with Western SSA leading improvements, while Southern SSA’s historical peaks reflected the co-epidemic impact of HIV/AIDS (Figures 4A2, A4).

### 3.8 Projections of tuberculosis incidence and mortality in Sub-Saharan Africa, sub-regions, and 22 selected countries from 2015 to 2035 compared with WHO End TB 2035 strategy targets

**Figure 5:**
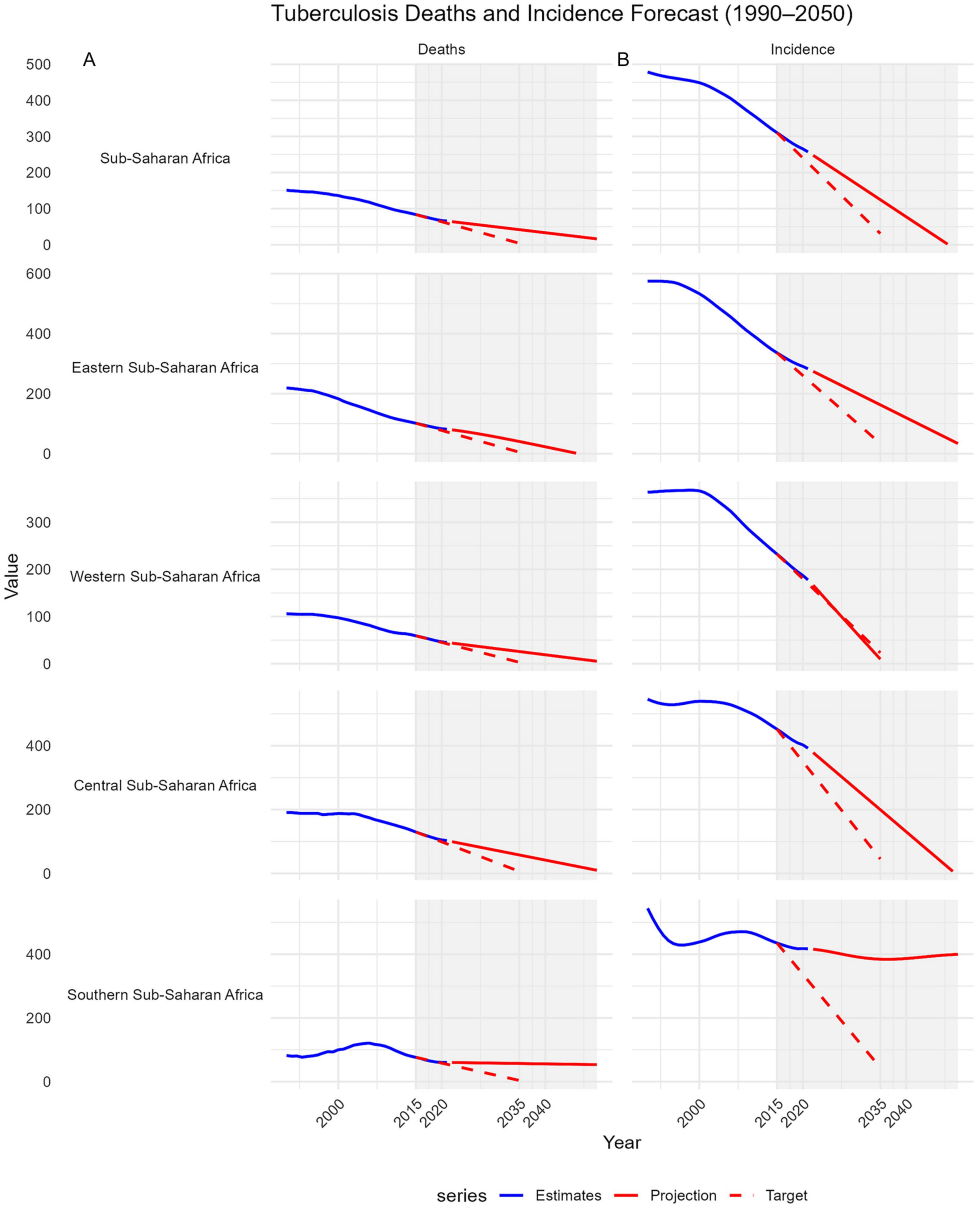
Projections of tuberculosis mortality and incidence in Sub-Saharan Africa and its sub-regions from 2015 to 2035 compared with the WHO End TB Strategy 2035 targets. Trends from 1990 to 2050 are shown. Each panel includes GBD 2021 estimates (blue line), ARIMA-based projections to 2050 (solid red line), and the WHO End TB 2035 targets (dashed red line), which aim for a 95% reduction in deaths and a 90% reduction in incidence from 2015 levels. A shaded area highlights the 2015–2050 target period. The x-axis ticks are fixed (2000, 2010, 2020, 2030, 2040, 2050), and negative projections were truncated. Faceted plots with free y-axis scales allow for comparison of sub-region-specific trajectories toward End TB milestones. Panel **(A)**: Deaths trends. Panel **(B)**: Incidence trends.

Using ARIMA modeling of GBD 2021 data, we projected MTB incidence and mortality in SSA and its subregions from 1990 to 2050, benchmarking against the WHO End TB Strategy targets of a 90% incidence reduction and 95% mortality reduction by 2035 from 2015 levels. SSA as a whole is projected to achieve a 49.8% reduction in mortality by 2035 (from 83.66 to 41.95 per 100,000), falling short of the 95% target (4.18 per 100,000) (Figure 5A).

Western SSA leads with a projected 56.4% reduction (59.17 to 25.79), followed by Eastern SSA (60.0%, 101.59 to 40.68), Central SSA (55.4%, 129.70 to 57.83), and Southern SSA (25.3%, 76.25 to 56.95). No subregion is projected to meet the mortality target. For incidence, SSA is projected to achieve a 59.8% reduction (from 310.35 to 124.68 per 100,000) by 2035, below the 90% target (31.04 per 100,000). Only Western SSA is on track to meet or exceed the target, with a projected 95.9% reduction (232.48 to 9.54). Eastern SSA (51.6%, 336.24 to 162.67) and Central SSA (55.9%, 451.98 to 199.38) fall short, while Southern SSA is projected to achieve only an 11.7% reduction (435.18 to 384.45), far from the target (Figure 5B).

**Figure 6:**
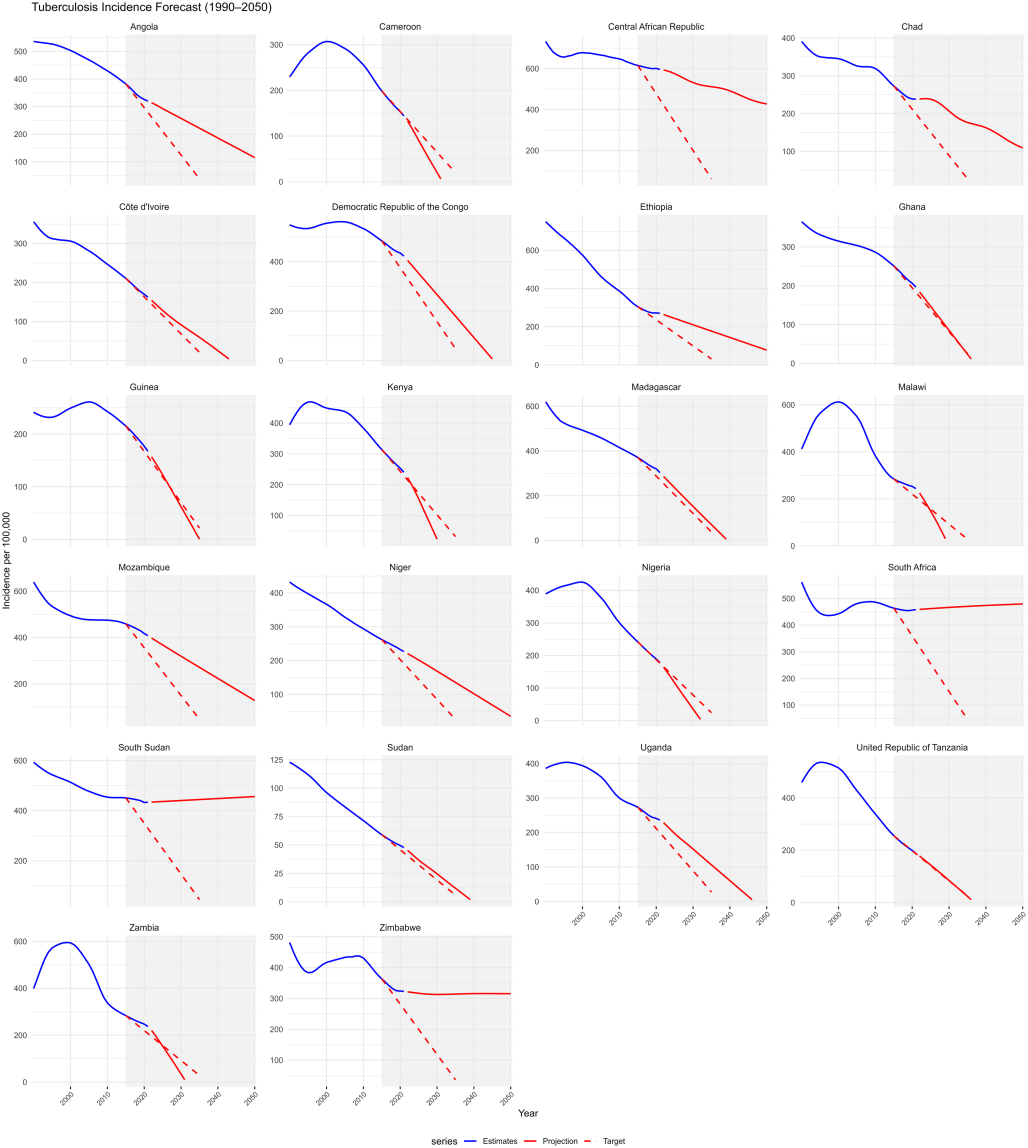
Projections of tuberculosis incidence for 22 selected countries in Sub-Saharan Africa from 2015 to 2035 compared with the WHO End TB Strategy 2035 targets. Trends from 1990 to 2050 are shown. Each panel includes GBD 2021 estimates (blue line), ARIMA-based projections to 2050 (solid red line), and the WHO End TB 2035 targets (dashed red line), which aim for a 90% reduction in incidence from 2015 levels. A shaded area highlights the 2015–2050 target period. The x-axis ticks are fixed (2000, 2010, 2020, 2030, 2040, 2050), and negative projections were truncated. Faceted plots with free y-axis scales allow for comparison of country-specific trajectories toward End TB milestones.

At the country level, none of the 22 included countries is projected to achieve the 95% mortality reduction target by 2035. However, Angola, Tanzania, Côte d’Ivoire, Niger, and Guinea demonstrated the largest mortality declines (64–78%) but still fell short (Supplementary Figure S6). Progress towards the 90% incidence reduction target was uneven; Guinea, Tanzania, and Ghana are projected to meet or surpass the target, with reductions exceeding 90%, whereas most countries including Angola, Côte d’Ivoire, and Niger, show moderate declines (40–72%), indicating insufficient progress (Figure 6).

### 3.9 Projections of the burden of tuberculosis and drug-resistant forms in Sub-Saharan Africa from 2021 to 2050

**Figure 7:**
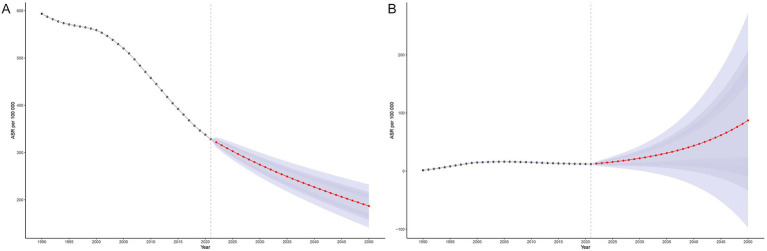
Projected age-standardized incidence rates for tuberculosis and multidrug-resistant tuberculosis in Sub-Saharan Africa, 1990–2050, using the Bayesian Age-Period-Cohort (BAPC) model. Panel **(A)**: Age-standardized incidence rate (ASIR) of *Mycobacterium tuberculosis* (MTB). Panel **(B)**: Age-standardized incidence rate (ASIR) of multidrug-resistant tuberculosis (MDR-TB).

**Figure 8:**
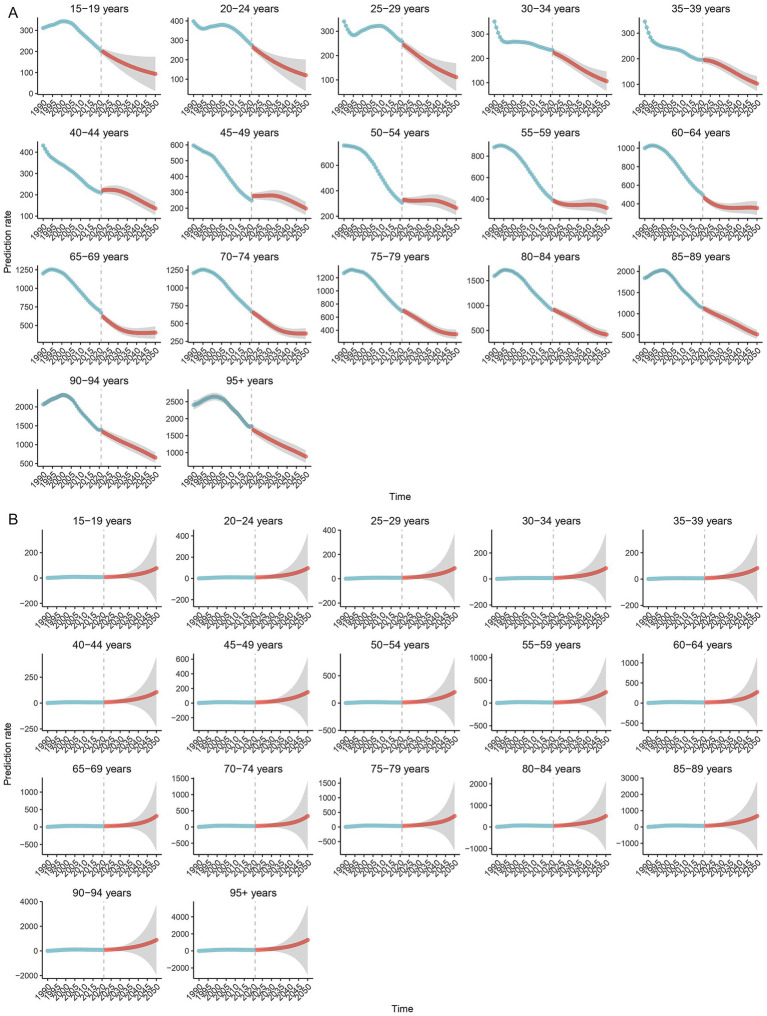
Projected age-specific incidence of tuberculosis and multidrug-resistant tuberculosis in Sub-Saharan Africa, 1990–2050, using the Bayesian Age-Period-Cohort (BAPC) model. Panel **(A)**: Incidence of *Mycobacterium tuberculosis* (MTB). Panel **(B)**: Incidence of multidrug-resistant tuberculosis (MDR-TB).

**Figure 9:**
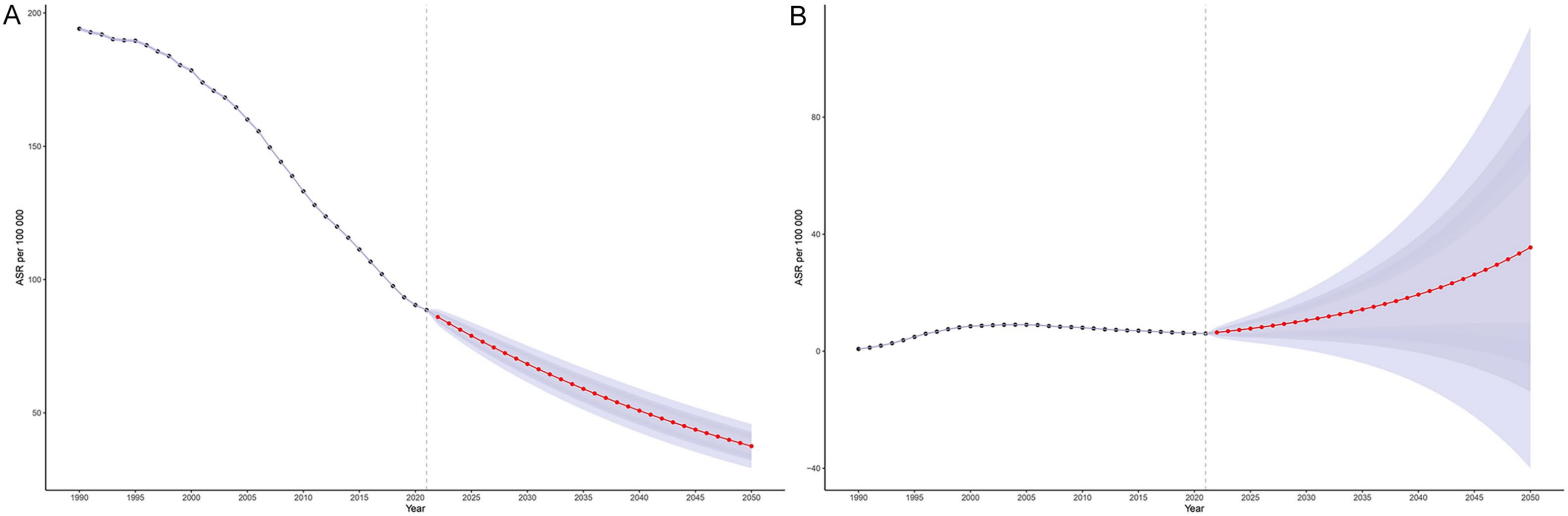
Projected age-standardized mortality rates (ASMR) for tuberculosis and multidrug-resistant tuberculosis in Sub-Saharan Africa, 1990–2050, using the Bayesian Age-Period-Cohort (BAPC) model. Panel **(A)**: ASMR of *Mycobacterium tuberculosis* (MTB). Panel **(B)**: ASMR of multidrug-resistant tuberculosis (MDR-TB).

Bayesian age–period–cohort (BAPC) modeling based on historical data (1990–2021) indicates a continued overall decline in MTB burden in SSA through 2050. The ASIR of MTB is projected to decrease steadily across all age groups, although the pace of decline slows after 2020 (Figure 7A). In contrast, ASIR of MDR-TB is projected to follow a markedly different trajectory. After plateauing at approximately 12 cases before 2020, the ASIR of MDR-TB is projected to rise sharply, reaching approximately 90 cases per 100,000 population by 2050 (Figure 7B). This increase is expected to be concentrated among older adults (≥85 years), with incidence rates projected to reach approximately 670 to 1284 per 100,000 in these age groups (Figure 8B). While the ASMR for MTB is projected to continue declining across all ages from approximately 90 to 37 per 100,000 from 2020 to 2050 (Figure 9A), this progress will be offset by a substantial rise in ASMR of MDR-TB, from approximately 6 deaths in 2020 to 35 deaths per 100,000 by 2050 (Figure 9B). These projections highlight a threat from drug-resistant TB strains that could erode decades of gains in reducing MTB mortality (Figure 8A).

## 4 Discussion

Our systematic analysis of the tuberculosis (TB) burden in 22 Sub-Saharan African (SSA) countries from 1990 to 2021, based on the Global Burden of Disease (GBD) 2021 study, highlights substantial progress alongside enduring challenges. We observed notable declines in age-standardized incidence rates (ASIR) for TB (46.2%), age-standardized mortality rates (ASMR) (56.4%), and disability-adjusted life-years (DALYs) (61.0%). These improvements stem from concerted efforts in case detection, treatment rollout, and international support, yet they are threatened by rising multidrug-resistant TB (MDR-TB) and extensively drug-resistant TB (XDR-TB), high HIV coinfection, and systemic health deficiencies. This epidemiological landscape reveals subregional heterogeneity and varying policy effectiveness, complicating the path to the WHO End TB Strategy targets. By synthesizing these trends and evaluating health system responses, we aim to inform future TB control strategies in SSA, emphasizing the need for integrated, equitable interventions to sustain momentum.

### 4.1 Epidemiological shifts in tuberculosis burden (1990–2021)

The TB profile in SSA has transformed significantly, influenced by urbanization, demographic changes, and enhanced case management practices that have improved detection and adherence. These reductions in incidence and mortality mirror broader trends in the WHO African Region, which achieved a 24% incidence decline from 2015 to 2023, exceeding the first WHO End TB milestone (Salam et al., 2023). Countries like South Africa, Kenya, and Zambia have seen incidence reductions exceeding 50%, with Western SSA showing particular success through targeted programs. Similarly, TB mortality dropped by 42% in the region between 2015 and 2023, the largest global decline, led by Kenya, Mozambique, Nigeria, Uganda, and Zambia. This reflects intensified case finding, better treatment adherence, and HIV-TB integration, which have collectively reduced the overall burden. Nevertheless, progress is uneven, creating pockets of vulnerability. High-burden countries such as the Democratic Republic of Congo (DRC), Central African Republic (CAR), Somalia, Ethiopia, and Sudan lag due to political instability, inadequate infrastructure, and funding shortages. These factors disrupt surveillance and service delivery, allowing TB to persist at high levels. Such issues highlight how weak surveillance and resource constraints can stall advancements, emphasizing the need for tailored interventions to mitigate subregional disparities and advance toward 2030 targets (Xue et al., 2022; Gele and Bjune, 2010; Marou et al., 2024). Addressing these requires not only increased funding but also adaptive strategies that account for local contexts, such as mobile clinics in unstable areas.

### 4.2 Subregional and national variations in drug-susceptible and resistant TB

Variations in DS-TB and DR-TB underscore diverse challenges across SSA, reflecting differences in health infrastructure and socioeconomic conditions. Eastern SSA, spearheaded by Ethiopia, has reduced DS-TB mortality and DALYs through consistent program implementation, yet faces rising MDR-TB and XDR-TB in Uganda, Kenya, and South Sudan, where resistance surveillance is limited. Central SSA, especially CAR, shows the highest per capita TB mortality and DALYs, with Angola making modest DS-TB gains but grappling with increasing drug resistance amid economic strains. Southern SSA is polarized: Zambia demonstrates comprehensive reductions across DS-, MDR-, and XDR-TB via effective National TB Programs (NTPs) that emphasize community involvement, while Zimbabwe experiences rising mortality due to service disruptions from economic and political turmoil. South Africa, despite high HIV co-infection, has achieved modest improvements through robust public health responses. Western SSA demonstrates moderate DS-TB control, with Côte d’Ivoire effectively containing MDR-TB. However, Nigeria and Ghana face escalating DR-TB burdens. These patterns stem from shared systemic gaps, including limited drug-resistance surveillance and access to second-line treatments, particularly in Uganda, Kenya, and South Sudan (Lukoye et al., 2015). Service disruptions and deteriorating health infrastructure, as seen in Zimbabwe and Angola, contribute to rising TB mortality and prevent continuity of care (Zhong et al., 2025). South Africa’s integrated systems offer some resilience against HIV co-infection by linking TB and HIV services effectively (Engelbrecht et al., 2018). Antibiotic misuse in under-regulated private sectors in South Africa, Nigeria, Kenya, and Tanzania accelerates MDR-TB, often through empirical prescribing without bacteriological confirmation, which promotes selective pressure for resistance (Salomon et al., 2022; Fadare et al., 2019; Gulumbe and Faggo, 2019). This compounded by weak regulatory oversight and fragmented systems compromise adherence and foster resistance to first-line drugs like isoniazid and rifampicin. Without substantial reforms, such as enhanced regulatory enforcement and public-private partnerships, MDR-TB incidence could rise by 2050, necessitating stronger diagnostic, regulatory, and treatment frameworks to prevent this from happening.

### 4.3 The HIV–TB syndemic in SSA

The HIV–TB syndemic amplifies SSA’s disease burden via complex biological interactions, where HIV weakens immunity, increasing TB susceptibility, and social factors that hinder care access. Expanded antiretroviral therapy (ART) and TB treatment have reduced HIV-associated DS-TB by improving immune recovery and early detection, but MDR- and XDR-TB prevalence among HIV-positive individuals is increasing, especially in Southern and Eastern SSA, due to diagnostic delays and resistance management challenges that allow resistant strains to proliferate. South Africa exemplifies this, with 61% HIV–TB coinfection and 18.8% HIV prevalence among adults aged 15–49, straining systems through high caseloads and resource demands. Similar rates appear in Western Kenya (42%) and Malawi, where stigma, often linking TB to HIV, delays care-seeking and perpetuates transmission cycles (Mbuthia et al., 2020; Hayward et al., 2024). Cultural reliance on traditional healers, seen in 51.3% of Zambia’s Nubi community and widely in Ghana, postpones biomedical intervention, aiding community transmission by allowing untreated cases to spread (Msoka et al., 2021; Gyimah and Dako-Gyeke, 2019). Stigma, with 41% self-stigmatization in Kenya, deters seeking help due to fears of social isolation, further exacerbating delays (Otieno et al., 2024). Mental health issues like anxiety impair adherence, underscoring the need for integrated psychosocial support to address emotional barriers. Rising diabetes, linked to high fasting plasma glucose, complicates the syndemic by adding metabolic vulnerabilities, requiring TB integration into non-communicable disease (NCD) frameworks to manage multiple comorbidities holistically (Obels et al., 2022).

### 4.4 Evolving risk factors – behavioral and metabolic drivers

TB risk factors in SSA are shifting from behavioral to metabolic drivers, aligning with epidemiological transitions driven by lifestyle changes and urbanization. Alcohol and tobacco remain prominent, especially in Southern and Eastern SSA. Alcohol misuse increases DR-TB risk via immunosuppression, malnutrition, and non-adherence, with 18.7–21.9% alcohol use disorder prevalence among MDR-TB patients in Nigeria and Uganda, illustrating how behavioral factors compound treatment challenges in SSA (Lasebikan and Ige, 2020; John and Oleka, 2022; Naidoo et al., 2013; Bayigga et al., 2025; Peltzer and Louw, 2014), while Tobacco is the leading risk factor in Eastern SSA through respiratory damage (Lamuka et al., 2018). Notably, high fasting plasma glucose surpassed tobacco as a leading factor in 2021, reflecting the growing burden of metabolic co-morbidities like diabetes, which impair immune responses. Elevated BMI, tied to diabetes and obesity, dominates in Central and Southern SSA, signaling a need for nutrition-focused interventions. Western SSA’s lower tobacco risk suggests effective regulations, but dietary risks and diabetes demand greater attention to prevent further escalation. Environmental factors add complexity, including zoonotic transmission via raw camel milk in Kenya (3.1% MTBC prevalence), which introduces resistant strains from animal reservoirs, and antibiotic resistance genes in Nigerian wastewater, highlighting pollution’s role in DR-TB spread (Lamuka et al., 2018; Mtetwa et al., 2023). Poverty and overcrowding fuel transmission in low SDI areas like Central SSA by facilitating close-contact spread (Chen et al., 2024). The absence of robust NCD surveillance and integrated care models underscores the urgent need for coordinated policy responses. Drawing lessons from successful HIV service expansion, a comprehensive “One Health” approach is essential to address the interconnected human, animal, and environmental dimensions of TB risk, promoting holistic prevention (Gouda et al., 2019).

### 4.5 Age and sex disparities in TB incidence and mortality

Age- and sex-specific patterns reveal MTB and MDR-TB incidence peaking in youth, with females bearing a greater burden, likely due to gender-specific vulnerabilities like malnutrition or higher HIV co-infection rates, as seen among young South African women (Perumal et al., 2018). Beyond biological vulnerabilities, socio-cultural and environmental factors likely contribute to the disproportionate TB and MDR-TB burden among young women in Sub-Saharan Africa (Hargreaves et al., 2011). In many settings, adolescent girls and young women spend prolonged hours indoors due to domestic and care-giving responsibilities, often in poorly ventilated household conditions that increases exposure risk. The widespread use of biomass fuels for cooking; a predominantly female role, further increases exposure to household air pollution (HAP) that compromises respiratory health and is associated with increased TB risk (Jagger et al., 2024). Multinational data from CLEAN-Air (Africa) reported mean kitchen concentrations of 119 μg/m^3^ and female cook exposures of 64 μg/m^3^ across Ghana, Kenya, and Cameroon (Williams et al., 2025), both substantially exceeding the WHO 2021 guideline of 15 μg/m^3^ (World Health Organization, 2021). In Uganda and Ethiopia, adult women experienced the highest exposures (177–205 μg/m^3^ respectively), while young women had intermediate exposures, about two-thirds of adult women’s levels. Adult men, young children, and the elderly experienced lower exposures, reflecting their limited involvement in cooking (Okello et al., 2018). Similar patterns were observed using carbon monoxide (CO) measurements, underscoring the disproportionate HAP burden borne by women and girls in these settings (Okello et al., 2018). Collectively, these socio-cultural and environmental exposures plausibly explain the higher MDR-TB burden observed among younger females and underscore the need for gender-responsive TB interventions such as household infection control education, improved ventilation, and clean energy cooking transitions to accelerate progress toward the WHO End TB targets.

Incidence rises in older adults, indicating reactivation of latent infections, a trend that could intensify with aging populations in the region by 2050, placing additional pressure on geriatric care. Mortality and DALYs peak in mid-life, with males more affected, linked to occupational exposures, smoking, alcohol misuse, socioeconomic factors, or HIV, which disproportionately impact men’s health-seeking behaviors (World Health Organization African Region, 2025; Ledesma et al., 2022). In Zambia, male DR-TB predominance calls for gender-sensitive interventions like workplace screenings to target high-risk groups (Ombura et al., 2016; Tiruneh et al., 2023). Such disparities emphasize the need to address male-specific risks to reduce premature mortality in productive ages. MTB prevalence is gender-balanced, highest in youth by absolute cases and in the elderly by rates, suggesting chronic undiagnosed infections persisting over time, often due to delayed diagnosis in resource-poor settings (John and Oleka, 2022). MDR-TB shows a shift: younger females predominate, possibly from biological susceptibility or care access issues, while older males do so later, linked to adherence problems or cumulative exposures. Low-SDI regions see rising MDR/XDR-TB from socioeconomic challenges that limit treatment access (Zhang et al., 2023). This interplay signals antibiotic resistance burdening aging males by 2050, straining systems with increased chronic care needs. Projections indicate declining overall TB incidence and mortality but slowing post-2020, suggesting current strategies may be reaching efficacy limits amid emerging challenges like resistance. MDR-TB’s explosive rise reflects poor antibiotic stewardship, diagnostic gaps, and potentially more transmissible variants, threatening reversals. Older adults’ vulnerability, from cumulative exposure, comorbidities, or immunosenescence, underscores urgent needs for enhanced surveillance, MDR-TB treatment optimization, and robust antimicrobial stewardship programs to meet WHO targets.

### 4.6 Health policy evaluation and progress toward targets

We evaluated TB control policies in 22 SSA countries, analyzing structural, operational, and financial aspects and their influence on outcomes, using GBD 2021, WHO, and national data to identify alignment with End TB goals and barriers. NTPs, under Ministries of Health, manage plans, procurement, and surveillance via hierarchical models: tertiary hospitals (e.g., St. Peter’s in Ethiopia, Parirenyatwa in Zimbabwe) handle complex MDR-TB cases, primary centers deliver DOTS for routine care, and Community health workers (CHWs) boost rural detection through grassroots outreach (Muluneh et al., 2021; Mugauri Dumisani et al., 2022). Understaffing and logistical bottlenecks hinder coordination, with international aid from the Global Fund and USAID proving crucial in conflict zones like DRC’s North Kivu, where disruptions often compromise data integrity (Murray et al., 2022).

Across SSA, Policies align with WHO’s End TB Strategy, promoting DOTS and Xpert MTB/RIF for rapid diagnosis, but implementation gaps persist: utilization at 27% in Nigeria (2019), 6% in DRC, 40% in South Africa (2016), limiting early detection (Williams et al., 2022; Cazabon et al., 2018). DS-TB DOTS success varies (66% Angola to 90% Zambia); MDR-TB management, using regimens like BPaLM/BPaL, is urban-centralized in Ethiopia, South Africa, and Zambia, restricting rural access and yielding success rates from 50% in CAR to 70% in Cameroon (Pradipta et al., 2021). TB-HIV integration varies (12% DRC to 60% Zimbabwe), affecting co-management. Nigeria’s FAST strategy reduced diagnostic delays (2.9 to 1.9 days) and boosted notifications (14–56%), offering a replicable model for active case finding (Useni et al., 2016). The COVID-19 pandemic reduced notifications in 2020–2021, especially in conflict areas, highlighting vulnerability in potential pandemic preparedness (Ambia et al., 2017; Hogan et al., 2020). Scaling diagnostics, as in Zambia (32–50% increase) and Botswana (27–80%), is key to improving case finding and control (Emerson et al., 2016).

Health systems face weaknesses such as paper-based registers in Angola, Uganda, Malawi, Niger, and Zimbabwe lower detection rates and data quality; Uganda’s system is accurate for overall success but not good enough for distinguishing cure from treatment failure, risking mis-classification and informed decision-making (Robbiati et al., 2022; Ogbuabor and Onwujekwe, 2019; Izudi et al., 2022). Transition to digital case-based surveillance is essential for accuracy and retrieval. Stockouts, severe worker shortages, poor NCD integration, and widespread malnutrition exacerbate challenges, reducing service capacity (Tukamuhebwa et al., 2024; Kadia et al., 2021; Osman et al., 2021). Digital tools like Zambia’s SmartCare promise better management but face connectivity barriers in remote areas (Kaumba, 2023). Lessons from HIV guideline adoption advocate decentralization and improved logistics to enhance TB control efficiency (Ambia et al., 2017).

Funding relies heavily on donors (Global Fund approximately 70% in Mozambique, Zambia, Niger) (The Global Fund to Fight AIDS, Tuberculosis and Malaria, 2024); South Africa funded 54% domestically since 2015, setting a benchmark (Amendah, 2019). Domestic funding (46%) covers basics like salaries and first-line drugs, leaving gaps for diagnostics and second-line regimens; impending USAID 2025 cuts threaten these services (World Health Organization Regional Office for Africa, 2024). Patients face catastrophic costs: 71.8% in Benin, 80% in Zimbabwe, making the WHO African Region bearing the highest catastrophic burden (68%) globally, driven by poverty and non-adherence (Laokri et al., 2014; Timire et al., 2021; World Health Organization, 2023). A study in Ghana showed that Ghana’s NHIS shows no significant cost reduction, with 64.1% still facing catastrophic burdens even if the NHIS is hypothetically expanded to all TB patients, highlighting high catastrophic burden in vulnerable groups (Pedrazzoli et al., 2021). Corruption in Mali and Zambia diminishes fund effectiveness. NHIS in Ghana and Ethiopia offer partial protection but are limited for DR-TB; the absence of NHIS entirely in CAR, Chad, and South Sudan exacerbates the burden of TB in such countries (Njagi et al., 2018; Vellekoop et al., 2022; Eze et al., 2022). We advocate for social protection mechanisms, like exemptions for older patients, to alleviate these financial hardships.

The ARIMA projections showed a 49.8% mortality reduction by 2035 using 2015 as a baseline (83.66 to 41.95 per 100,000) and a 59.8% incidence drop (310.35 to 124.68). None of the selected countries is currently on track to meet the WHO’s 2035 milestones of a 95% reduction in TB mortality, indicating an insufficient pace. However, Guinea, Tanzania, and Ghana show progress toward the WHO milestones of a 90% reduction in TB incidence; sub-regionally, Western SSA showed notable progress in meeting the WHO incidence targets, aligning with WHO 2024 reports (with 8 of 15 countries that met the 2015–2020 milestone) and our trends indicating significant progress sub-regionally (Ledesma et al., 2024). Southern SSA’s 2006 rise in TB burden may be tied to the HIV epidemic; we recommend that health policymakers pay more attention to the HIV/TB burden (Lawn and Churchyard, 2009). Wastewater epidemiology in Cameroon reveals unauthorized drug access, undermining protocols and fueling resistance (Mtetwa et al., 2023). Without intensified policy commitment and sustained investments, most countries in SSA will remain off track to achieve the WHO End TB goals by 2035.

### 4.7 Strategic priorities, policy implications, limitations, and future research

For SSA to meet WHO End TB and SDG 3.4 targets, we believe the following recommendations would help address the MDR/XDR-TB rise, HIV-TB syndemic, and evolving risks.

First, to reduce reliance on donors, Sub-Saharan African (SSA) countries must increase domestic TB financing, aiming for at least 50% by 2030, as demonstrated in Ghana and South Africa (Amu et al., 2023). Harmonizing national health insurance schemes, like in Ghana and Kenya, is also critical to reduce catastrophic health expenditures, especially in countries such as the Central African Republic, Chad, and South Sudan where they are absent (Amu et al., 2023; Charity et al., 2020). Adopting a non-welfarist framework can ensure equitable resource allocation and financial protection, guaranteeing access to healthcare without causing poverty (Otim et al., 2020; MacPherson et al., 2019; Long et al., 2021).

Second, expanding Xpert MTB/RIF coverage to 80%, especially in rural areas, is critical due to its significant impact on improving detection rates, as evidenced in Zambia and Botswana (Emerson et al., 2016; Nyanda Elias et al., 2021). There is an urgent need to decentralize this technology, as usage is much lower at district-level (56%) than at tertiary hospitals (95–97%) in countries like Uganda, Tanzania, and Kenya (Nyanda Elias et al., 2021; Dheda et al., 2022). Furthermore, conducting DR-TB surveys in data-deficient nations (e.g., Sudan, Guinea) and scaling up molecular diagnostics like the Line Probe Assay (currently in only 4–5% of facilities) are essential to enhance surveillance and curb the projected 30% rise in resistance by 2050 (Kapata et al., 2025; Murray et al., 2022). National policies should also support decentralizing ART services to primary healthcare levels to improve access and reduce TB prevalence and mortality among TB/HIV coinfected patients.

Third, addressing healthcare workforce shortages requires a multi-faceted strategy. This involves task-shifting to community and lay health workers (as in Eswatini) and adopting rapid training models like Ghana’s two-year nursing diploma to build capacity and improve local retention in underserved areas (Naidoo et al., 2013; Bayigga et al., 2025; Peresu et al., 2020). Furthermore, implementing digital adherence technologies, such as South Africa’s “Wisepill evriMED” allows for real-time treatment monitoring to reduce DR-TB risk (Mukora et al., 2023). Effective coordination, including regular adherence checks, comprehensive training in TB infection prevention and control (TB-IPC), and measures like respirator use and patient isolation, is essential to reduce transmission and prevent the progression from drug-sensitive to multidrug-resistant TB (MDR-TB) (Kallon et al., 2022; Muhamed Awolu et al., 2024).

Fourth, a comprehensive integration of TB, HIV, non-communicable diseases (e.g., diabetes), and mental health services is vital to address this syndemic and reduce stigma-driven care delays. Community-based interventions, such as peer support and TB literacy campaigns, can significantly improve treatment adherence and curb transmission (Zegeye et al., 2023). Providing dedicated mental health support to address issues like anxiety and loneliness has been shown to enhance treatment outcomes, as seen in Kenya (Otieno et al., 2024; Nyasulu et al., 2018). Furthermore, adopting family-centered approaches can strengthen treatment adherence (Nyasulu et al., 2018; Lorent et al., 2015).

Fifth, prioritizing TB control in correctional facilities is crucial due to extremely high prevalence (up to 16.3%), driven by overcrowding, malnutrition, and HIV coinfection. This requires implementing proven strategies such as entry screening (as in Zambia) and pre-trial detention reforms (as in South Africa) (Dolan et al., 2016; Telisinghe et al., 2016; Kuupiel et al., 2020). Additionally, gender-sensitive interventions are essential to address disparities; these include workplace screenings targeting men (e.g., Zambia) and integrated TB-HIV programs for young women.

Sixth, strengthening TB laboratory networks and DR-TB surveillance is vital. These efforts, along with addressing zoonotic TB transmission routes (e.g., through raw camel milk in Kenya), requires a comprehensive “One Health” framework that explicitly integrates human, animal, and environmental health.

Seventh, a proactive approach to clinical TB research, facilitated by partnerships like the European & Developing Countries Clinical Trials Partnership (EDCTP), is essential for building sustainable research ecosystems within Sub-Saharan Africa (SSA). This requires investments in local infrastructure, training, and collaborative networks to reduce reliance on external expertise and improve preparedness for future health challenges (Nyirenda et al., 2021). Such capacity is crucial for conducting region-specific surveillance, especially given the diverse TB strain lineages found in countries like Nigeria, Cameroon, and Ghana (Chala and Usmael, 2020; Sahal et al., 2023).

### 4.8 Limitations and future research

This study has several limitations. First, it relies on Global Burden of Disease (GBD) 2021 modeled estimates, which may introduce bias, especially in conflict-affected countries such as South Sudan, where weak surveillance likely underestimates the TB burden. Second, because GBD estimates are largely derived from national TB notifications and passive surveillance, incomplete reporting and differences in diagnostic capacity may introduce selection bias and affect the accuracy of drug-resistant TB (DR-TB) estimates. Third, country-level differences in laboratory confirmation, diagnostic infrastructure, and data quality may have introduced heterogeneity in the estimates, warranting cautious interpretation. Fourth, the inclusion of 22 Sub-Saharan African countries, guided by disease burden, data availability, and regional representation, may have introduced selection bias; findings are therefore limited to these countries and may not be generalizable to all settings within Sub-Saharan Africa or beyond the region. Fifth, this study employed a targeted narrative literature review to contextualize GBD estimates. Future large-scale research may benefit from incorporating a systematic review to strengthen qualitative synthesis and enhance methodological rigor in country selection. Sixth, significant data gaps exist for pediatric TB, adolescent TB, and strain lineage, limiting the precision of targeted interventions. Seventh, under-notification in countries such as Ghana, the Central African Republic, and South Sudan, worsened by political instability and weak infrastructure (Kwabla et al., 2025).

Future studies should prioritize strengthening local surveillance and expanding standardized drug-resistance surveys, particularly for children and adolescents in high-burden countries like Nigeria, Cameroon, and Ghana. Key areas include assessing catastrophic costs in nations such as the Central African Republic and Chad (Pedrazzoli et al., 2021), integrating mental health and non-communicable disease care into TB services, and applying a “One Health” approach to address zoonotic TB and environmental DR-TB drivers. Building sustainable local research capacity, through initiatives like the European & Developing Countries Clinical Trials Partnership, is critical for generating context-specific evidence.

## 5 Conclusion

Tuberculosis remains a significant public health challenge in Sub-Saharan Africa, where progress is threatened by the rise of drug-resistant TB, high HIV coinfection, and systemic barriers like weak surveillance and funding gaps. With none of the 22 countries currently on track to meet the WHO’s 2035 mortality reduction target, however, Tanzania, Guinea, and Ghana are projected to meet the incidence targets, urgent action is needed through scaled-up diagnostics, strengthened health systems, integrated care for co-morbidities, and robust regional collaboration. Achieving the End TB goals will require intensified, equity-focused policies, sustainable financing, and comprehensive strategies tailored to the region’s specific challenges, including the looming MDR-TB crisis projected by 2050.

## Data Availability

Publicly available datasets were analyzed in this study. This data can be found at: https://vizhub.healthdata.org/gbd-results/.

## Footnotes

^1^ https://vizhub.healthdata.org/gbd-results/
