# Supplementary material for: The burden of tuberculosis and drug resistance in 22 Sub-Saharan African countries, 1990–2021: a GBD 2021 analysis and progress towards WHO 2035 targets with projections to 2050
Source: Front Microbiol. 2025 Nov 17;16:1695592. doi: 10.3389/fmicb.2025.1695592 (PMC12665714; doi:10.3389/fmicb.2025.1695592)

**Figure S1a**: **Proportion of Mycobacterium tuberculosis DALYs Attributable to Risk Factors (%) in Sub-Saharan Africa, 1990 and 2021.**
Percentage of tuberculosis DALYs in Sub-Saharan Africa attributable to risk factors like high alcohol use, high fasting plasma glucose, tobacco, high body-mass index, dietary risks, and low physical activity. Panel A compares proportions between 1990 (yellow) and 2021(blue) for the entire region using stacked bar charts. Panel B details the 2021 data across four sub-regions (Eastern - yellow, Central – Green, Western- Pink, and Southern Sub-Saharan Africa-red). Source: Global Burden of Disease (GBD) 2021 Study.


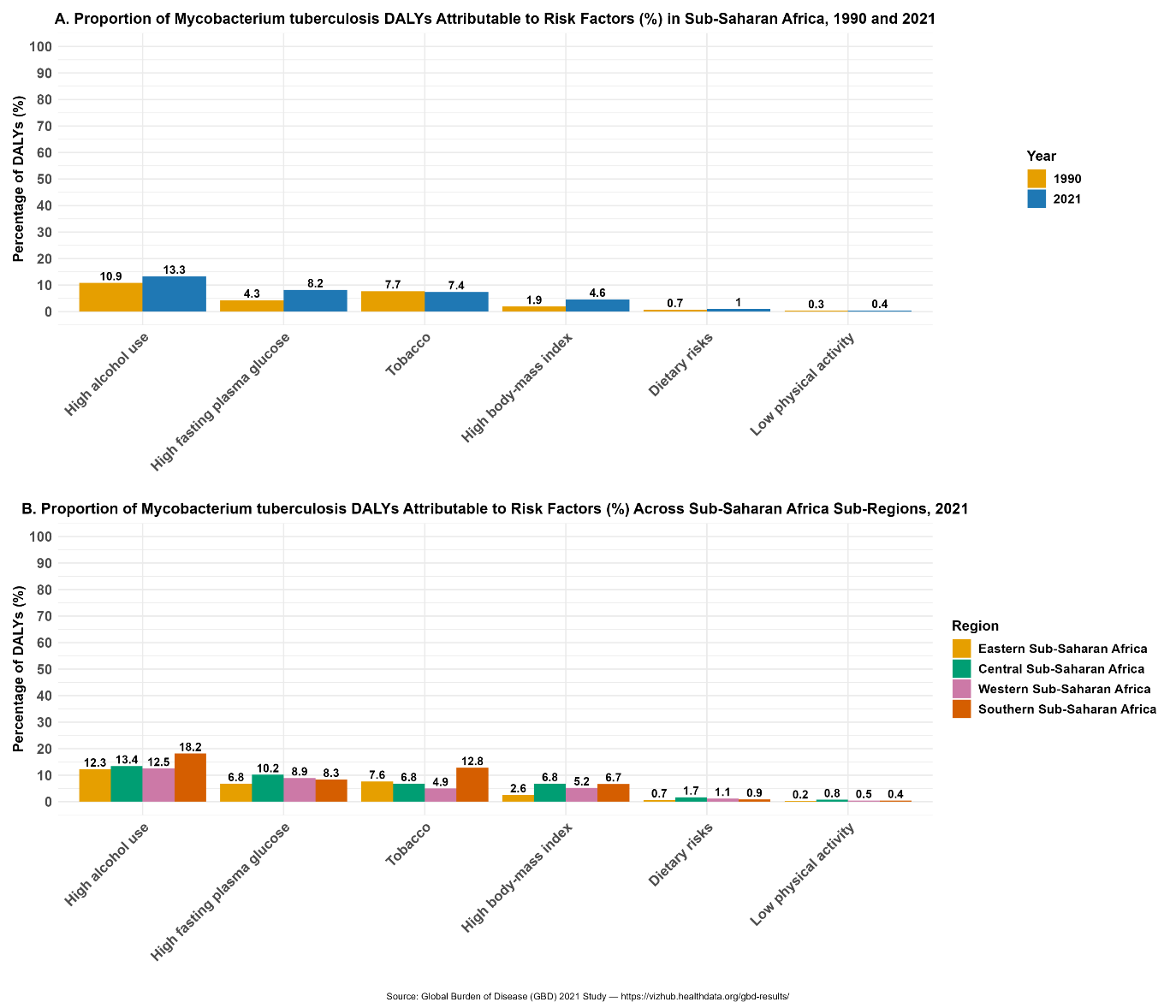


**Figure S1b: Proportion of Mycobacterium tuberculosis Deaths Attributable to Risk Factors (%) in Sub-Saharan Africa and Subregions, 1990 and 2021.**


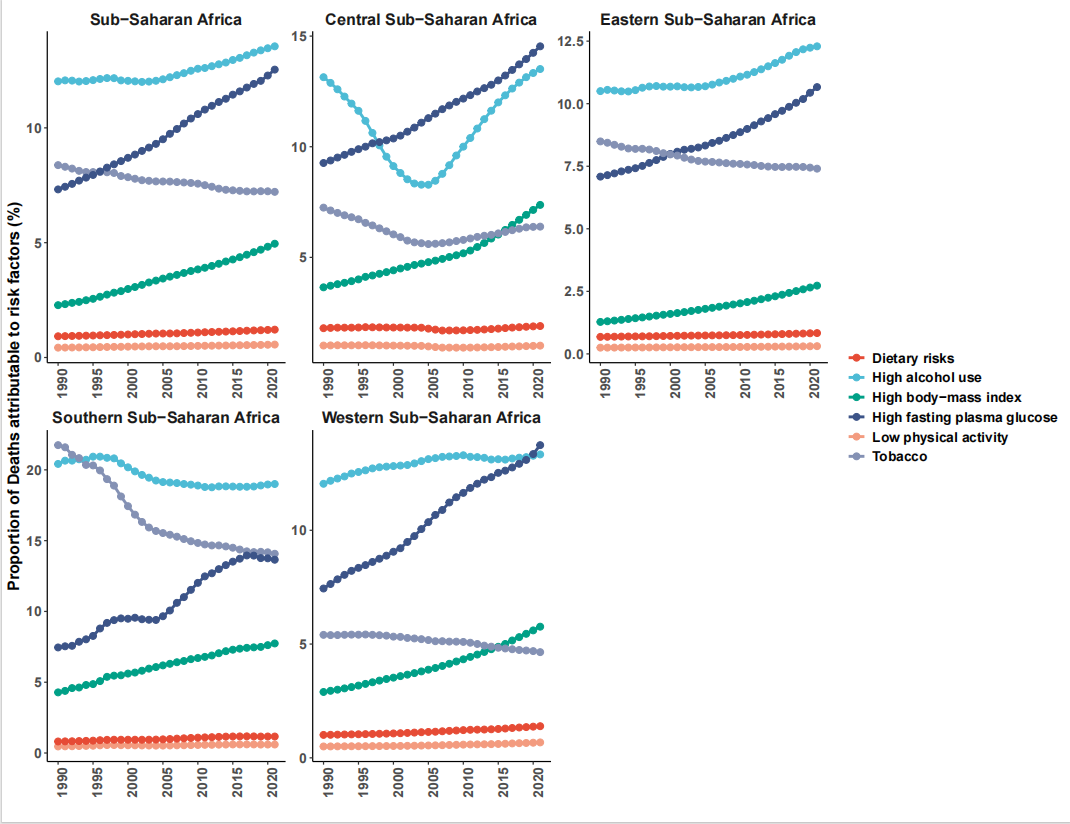


**Figure S2a: Age and Sex−Specific Burden of Drug-susceptible TB (number and rates) in Sub−Saharan Africa, 2021.**
Bar plots represent absolute numbers (with UI error bars) and line plots for rates per 100,000, scaled on a secondary y-axis. 3A: Age-standardized mortality (ASMR); 3B: Age-standardized incidence (ASIR), 3C: Age-standardized Prevalence (ASPR), 3D: Age-standardized disability-adjusted life years (DALYs).


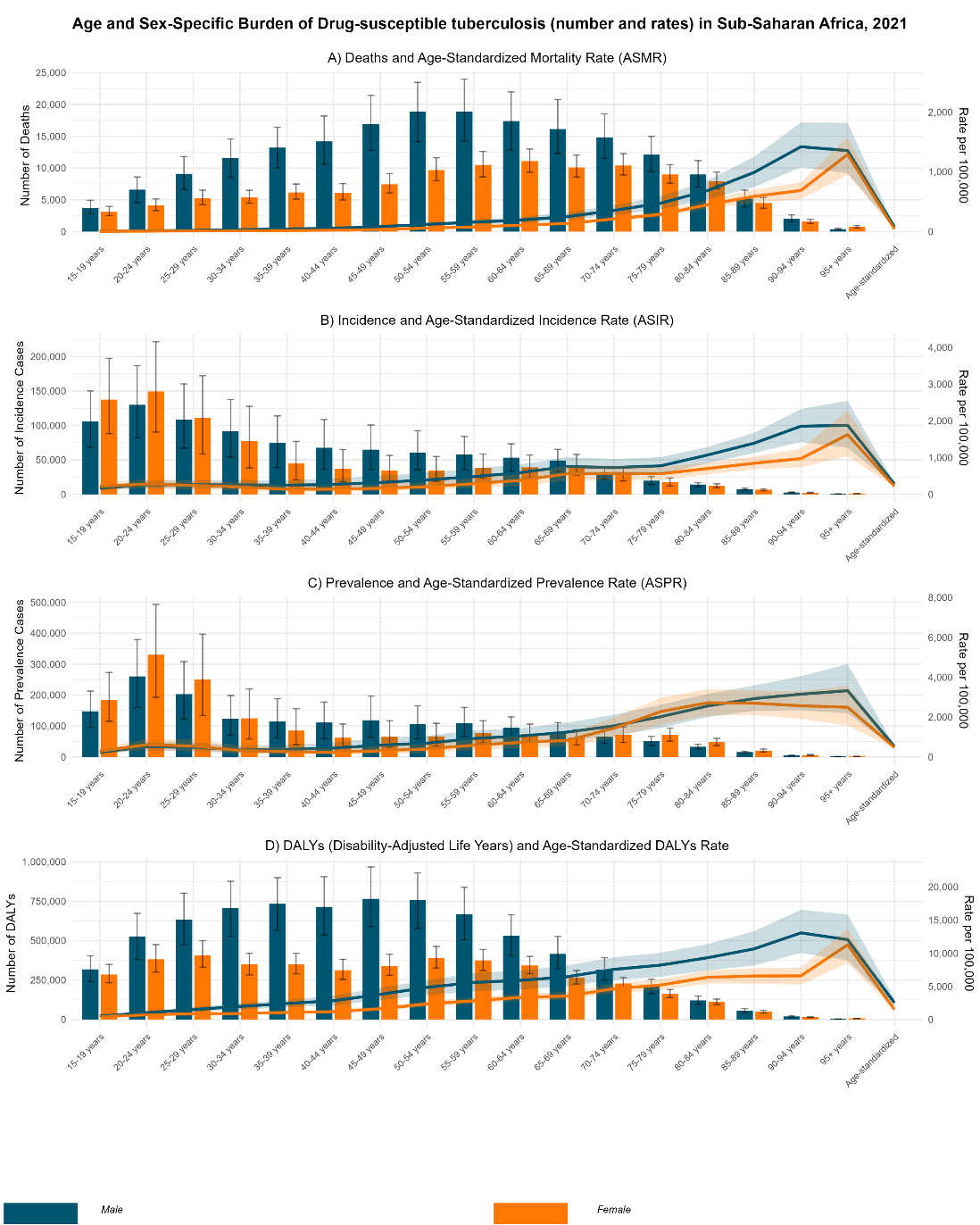


**Figure S2b: Age and Sex−Specific Burden of Extensively drug-resistant TB (number and rates) in Sub−Saharan Africa, 2021.**Bar plots represent absolute numbers (with UI error bars) and line plots for rates per 100,000, scaled on a secondary y-axis. 3A: Age-standardized mortality (ASMR); 3B: Age-standardized incidence (ASIR), 3C: Age-standardized Prevalence (ASPR), 3D: Age-standardized disability-adjusted life years (DALYs).


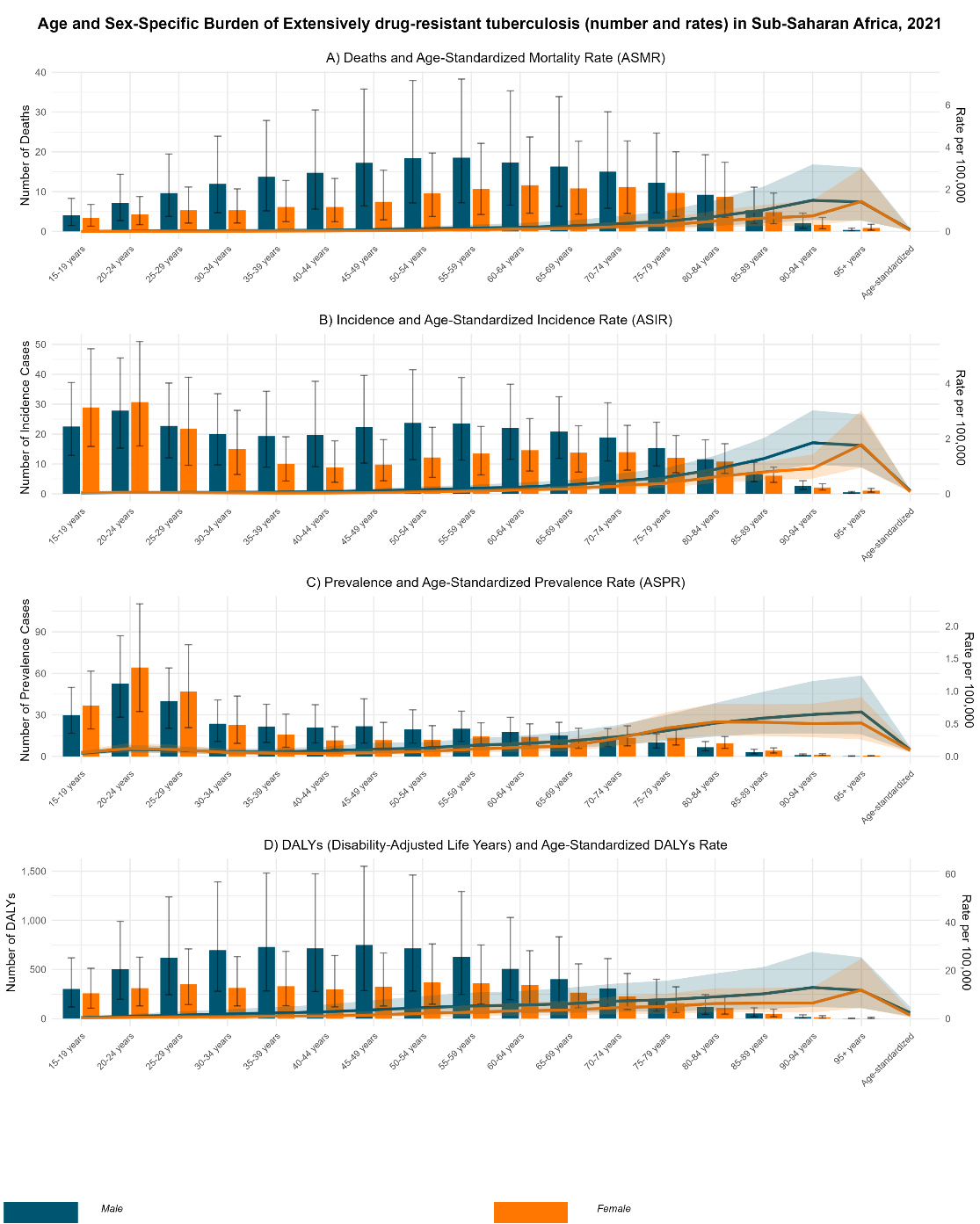


**Figure S3c. Drug Susceptible TB Temporal Trends in SSA 1990–2021**


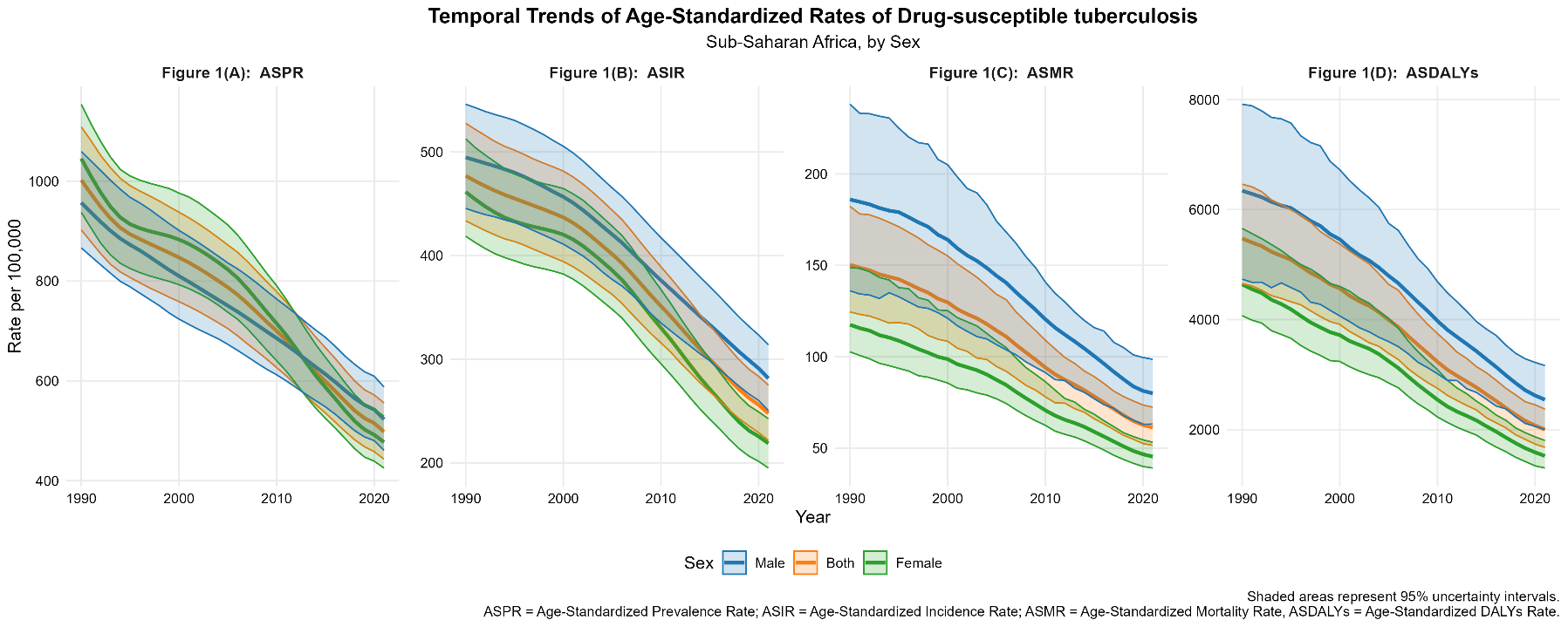


**Figure S3d. XDR-TB Temporal Trends in SSA 1990–2021**


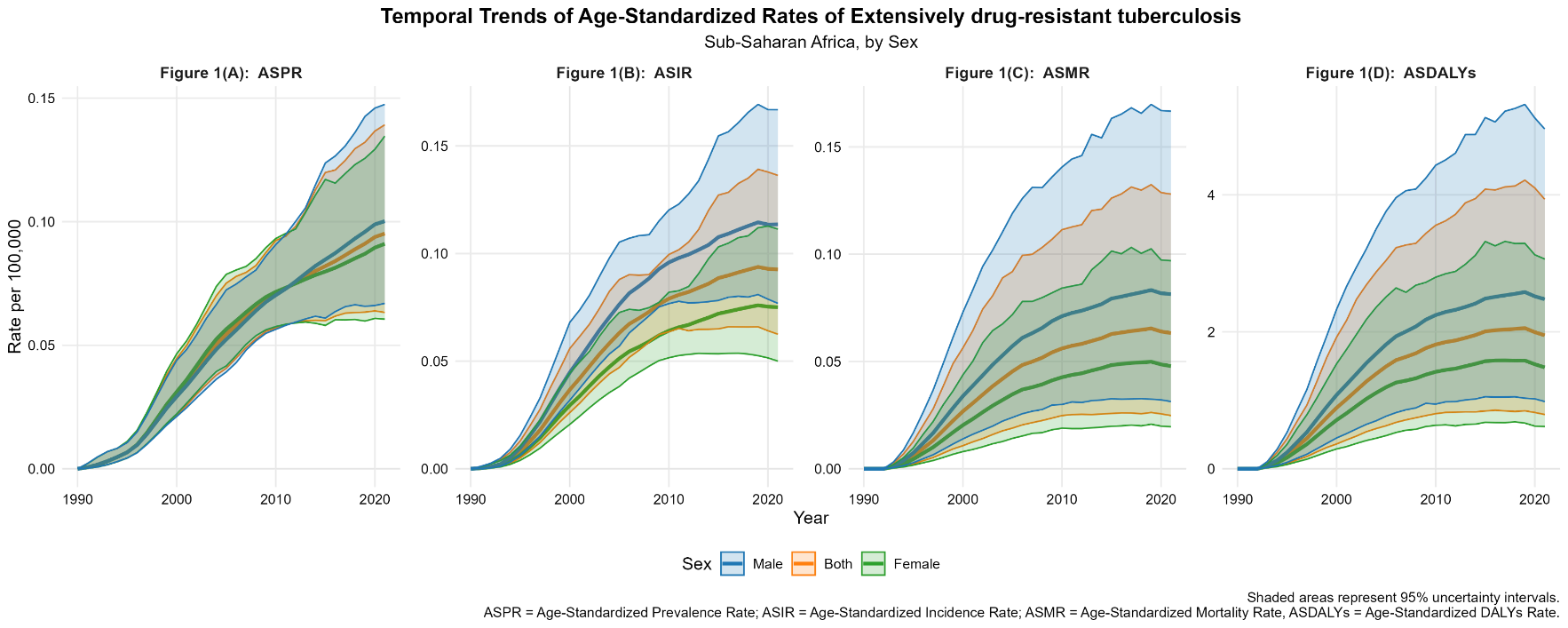


**Figure S4c. Subregional Temporal Trend of Drug-Susceptible TB Mortality, Incidence, Prevalence and DALYs, 1990–2021**
The plot represents trends from 1990 to 2021, line plots with 95% UIs, color-coded by subregion (Central: blue, Eastern: orange, Southern: green, Western: red). 6A: Age-standardized prevalence (ASPR), 6B: Age-standardized incidence (ASIR), 6C: Age-standardized mortality (ASMR); 6D: Age-standardized disability-adjusted life years (DALYs).


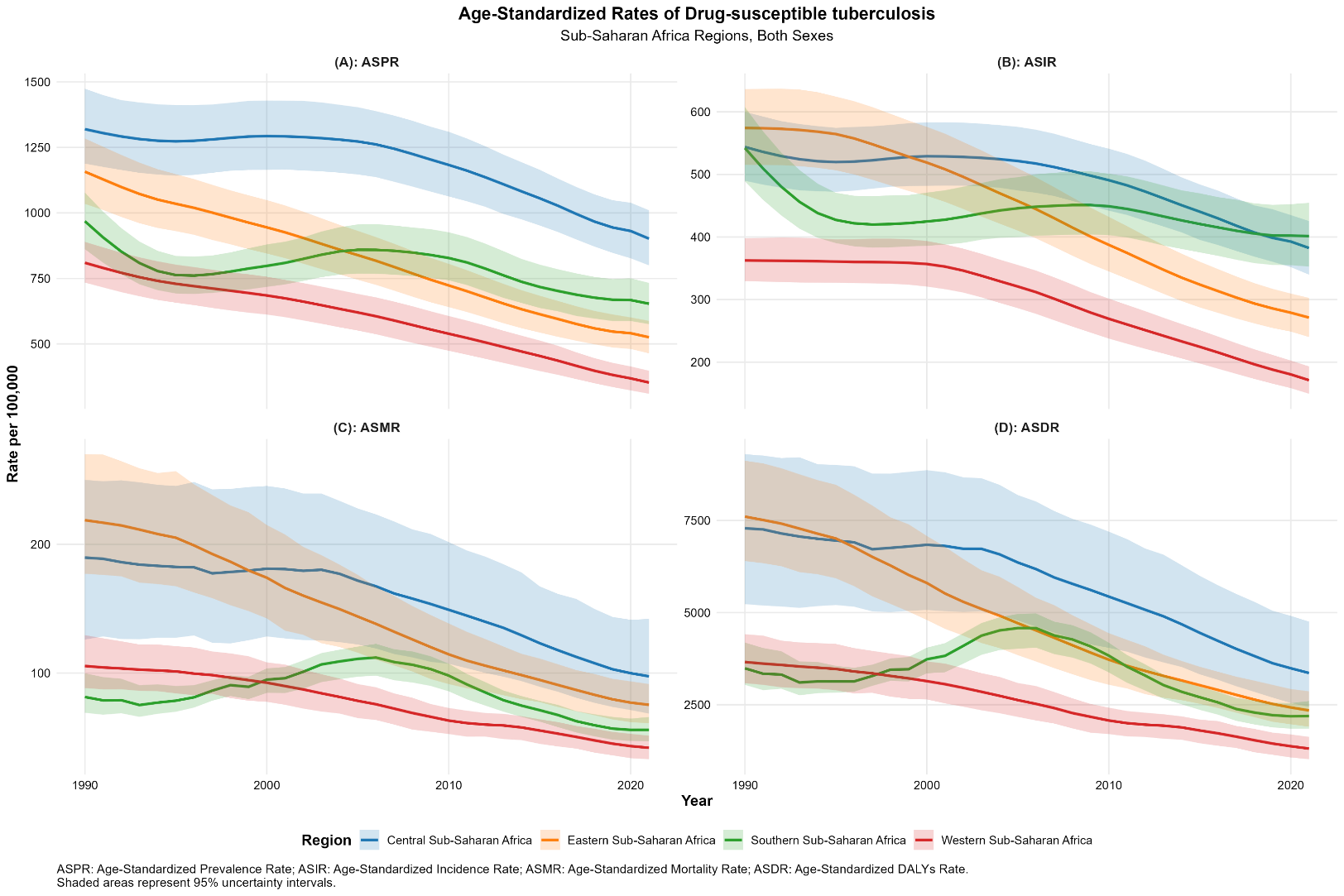


**Figure S4d. Subregional Temporal Trend of Extensively drug-resistant TB Mortality, Incidence, Prevalence and DALYs, 1990–2021**


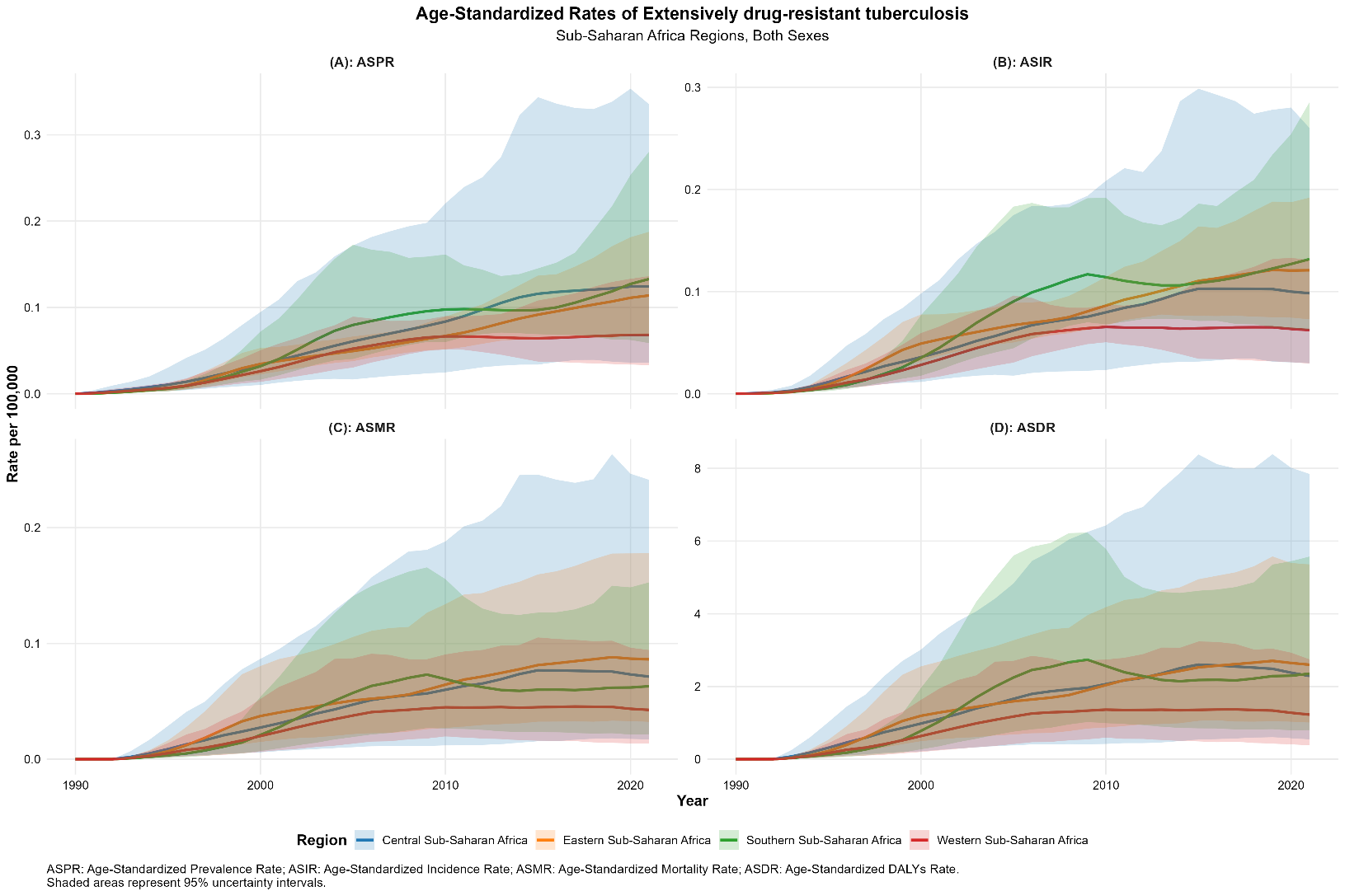


**Figure S6 shows Projections of Tuberculosis Mortality of 22 Selected Countries From 2015 to 2035 Compared with WHO End TB 2035 Strategy Targets**


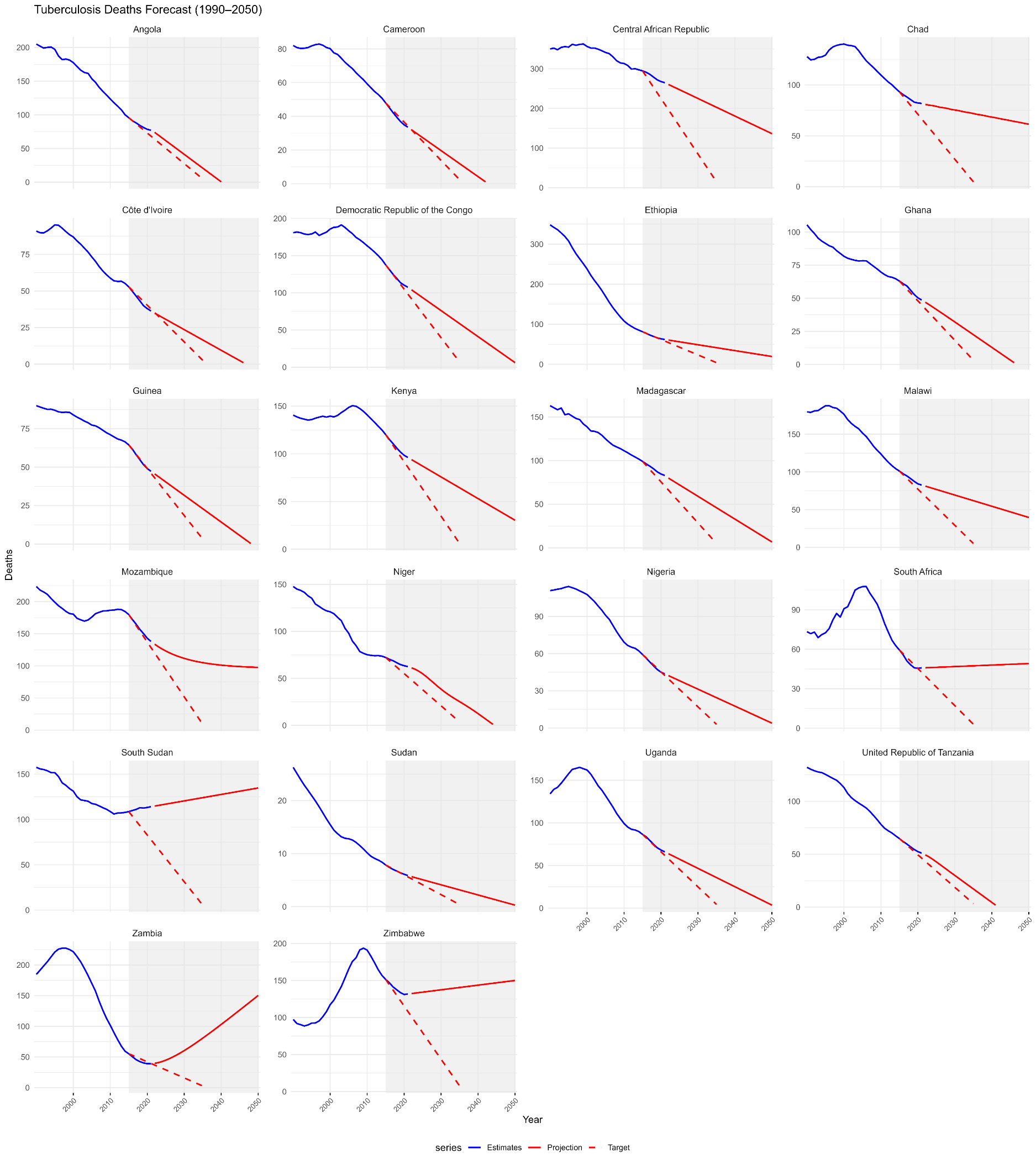


**Figure S7.** Projected age-specific mortality for tuberculosis and multidrug-resistant tuberculosis in Sub-Saharan Africa, 1990–2050, using the Bayesian Age-Period-Cohort (BAPC) model. **Panel A**: Mortality due to Mycobacterium tuberculosis (MTB). **Panel B:** Mortality due to multidrug-resistant tuberculosis (MDR-TB).


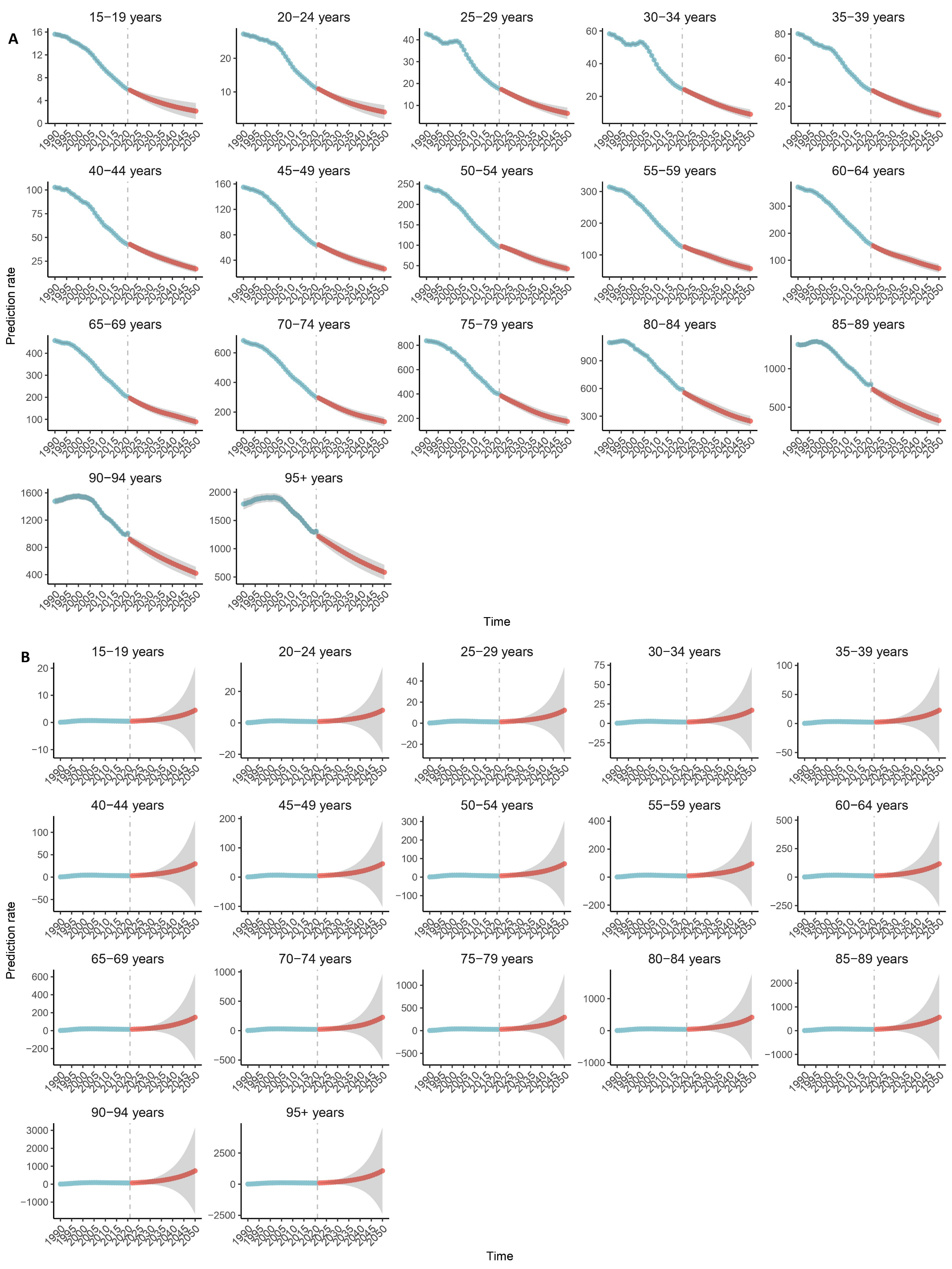

Supplement: Supplementary file 1 [file Table_1.docx]
