# Supplementary material for: The burden of tuberculosis and drug resistance in 22 Sub-Saharan African countries, 1990–2021: a GBD 2021 analysis and progress towards WHO 2035 targets with projections to 2050
Source: Front Microbiol. 2025 Nov 17;16:1695592. doi: 10.3389/fmicb.2025.1695592 (PMC12665714; doi:10.3389/fmicb.2025.1695592)
Supplement: Supplementary file 2 [file Table_2.docx]

**Table S1: Tuberculosis Death Number and Percentage Change in Sub-Saharan Africa and Sub-Regions between 1990 and 2021 based on GBD 2021 Report**

| Region | TB | | | DS-TB | | | MDR-TB | | | XDR-TB | | |
| --- | --- | --- | --- | --- | --- | --- | --- | --- | --- | --- | --- | --- |
|  | 1990 Number (95% UI) | 2021 Number (95% UI) | Deaths % Change (1990–2021) | 1990 Number (95% UI) | 2021 Number (95% UI) | Deaths % Change (1990–2021) | 1990 Number (95% UI) | 2021 Number (95% UI) | Deaths % Change (1990–2021) | 2000 Number (95% UI) | 2021 Number (95% UI) | Deaths % Change (2000–2021) |
| Sub-Saharan Africa | 433689 (366247, 518433) | 373075 (312734, 441700) | -13.98 (-14.61, -14.80) | 431960 (365120, 516031) | 346895 (287395, 414752) | -19.69 (-21.29, -19.63) | 1729 (645, 3853) | 25821 (11007, 52680) | 1393.12 (1606.81, 1267.41) | 94 (38, 203) | 359 (142, 731) | 283.97 (275.88, 260.13) |
| Western Sub-Saharan Africa | 119452 (99774, 145546) | 103684 (79411, 130735) | -13.20 (-20.41, -10.18) | 118771 (99067, 144862) | 96609 (74605, 121712) | -18.66 (-24.69, -15.98) | 681 (224, 1724) | 6978 (2365, 15585) | 925.38 (954.95, 804.25) | 28 (9, 73) | 97 (30, 220) | 243.84 (239.22, 202.53) |
| Eastern Sub-Saharan Africa | 220492 (186975, 264775) | 159769 (129447, 195009) | -27.54 (-30.77, -26.35) | 220097 (186459, 264174) | 147187 (119478, 181540) | -33.13 (-35.92, -31.28) | 394 (120, 1172) | 12409 (4808, 25856) | 3046.91 (3914.84, 2105.55) | 45 (18, 95) | 173 (66, 358) | 287.51 (260.43, 275.15) |
| Central Sub-Saharan Africa | 64115 (45303, 82265) | 70259 (49298, 101229) | 9.58 (8.82, 23.05) | 63719 (45004, 81733) | 66744 (46907, 96231) | 4.75 (4.23, 17.74) | 395 (73, 1498) | 3467 (865, 11863) | 777.32 (1087.22, 691.69) | 12 (3, 36) | 49 (11, 168) | 315.89 (341.96, 367.56) |
| Southern Sub-Saharan Africa | 29631 (25480, 36233) | 39363 (34352, 45831) | 32.84 (34.82, 26.49) | 29372 (25287, 35854) | 36355 (30307, 43281) | 23.78 (19.85, 20.71) | 259 (44, 812) | 2967 (1071, 6630) | 1044.17 (2336.08, 716.80) | 9 (3, 23) | 41 (14, 100) | 349.35 (351.59, 327.47) |
| TB: Tuberculosis; DS-TB: Drug-susceptible tuberculosis; MDR-TB: Multidrug-resistant tuberculosis; | | | | | | | | | | | | |
| XDR-TB: Extensively drug-resistant tuberculosis. | | | | | | | | | | | | |
| Values represent number and percentage change (95% Uncertainty Interval) from 1990 to 2021 for TB, DS-TB, and MDR-TB, and from 2000 to 2021 for XDR-TB. NA indicates data not available. | | | | | | | | | | | | |

**Table S2: Tuberculosis Incidence Number and Percentage Change in Sub-Saharan Africa and Sub-Regions between 1990 and 2021 based on GBD 2021 Report**

| Region | TB | | | DS-TB | | | MDR-TB | | | XDR-TB | | |
| --- | --- | --- | --- | --- | --- | --- | --- | --- | --- | --- | --- | --- |
|  | 1990 Number (95% UI) | 2021 Number (95% UI) | Incidence % Change (1990–2021) | 1990 Number (95% UI) | 2021 Number (95% UI) | Incidence % Change (1990–2021) | 1990 Number (95% UI) | 2021 Number (95% UI) | Incidence % Change (1990–2021) | 2000 Number (95% UI) | 2021 Number (95% UI) | Incidence % Change (2000–2021) |
| Sub-Saharan Africa | 1756526 (1581279, 1949642) | 2206541 (1954542, 2467218) | 25.62 (23.61, 26.55) | 1752530 (1577386, 1945649) | 2131367 (1886340, 2387208) | 21.62 (19.59, 22.69) | 3996 (2344, 6875) | 74549 (50106, 107234) | 1765.61 (2037.95, 1459.69) | 141 (100, 213) | 625 (421, 909) | 342.80 (320.27, 326.52) |
| Western Sub-Saharan Africa | 524357 (476277, 579519) | 601757 (533192, 676842) | 14.76 (11.95, 16.79) | 522817 (475034, 578161) | 581222 (513586, 656586) | 11.17 (8.12, 13.56) | 1540 (794, 2788) | 20368 (9571, 42196) | 1222.78 (1104.76, 1413.39) | 44 (21, 92) | 167 (79, 360) | 282.50 (269.39, 291.92) |
| Eastern Sub-Saharan Africa | 731640 (657335, 816915) | 860550 (755465, 974844) | 17.62 (14.93, 19.33) | 730966 (656224, 816040) | 827721 (723576, 940628) | 13.24 (10.26, 15.27) | 674 (289, 1645) | 32545 (19409, 53305) | 4730.22 (6615.81, 3141.33) | 63 (41, 97) | 283 (172, 451) | 350.89 (324.00, 362.39) |
| Central Sub-Saharan Africa | 232481 (206330, 257994) | 400096 (356013, 447996) | 72.10 (72.55, 73.65) | 231772 (205935, 256929) | 390858 (345839, 439819) | 68.64 (67.94, 71.18) | 708 (189, 2352) | 9162 (2772, 24002) | 1193.40 (1366.75, 920.69) | 16 (5, 44) | 76 (24, 199) | 373.03 (352.18, 355.95) |
| Southern Sub-Saharan Africa | 268049 (237160, 302367) | 344138 (300917, 393275) | 28.39 (26.88, 30.07) | 266975 (236318, 301630) | 331566 (289443, 381154) | 24.19 (22.48, 26.36) | 1074 (234, 3156) | 12473 (5635, 26528) | 1061.40 (2303.52, 740.58) | 18 (9, 41) | 98 (46, 206) | 431.85 (384.71, 396.57) |
| TB: Tuberculosis; DS-TB: Drug-susceptible tuberculosis; MDR-TB: Multidrug-resistant tuberculosis; | | | | | | | | | | | | |
| XDR-TB: Extensively drug-resistant tuberculosis. | | | | | | | | | | | | |
| Values represent number and percentage change (95% Uncertainty Interval) from 1990 to 2021 for TB, DS-TB, and MDR-TB, and from 2000 to 2021 for XDR-TB. NA indicates data not available. | | | | | | | | | | | | |

**Table S3: Tuberculosis Prevalence Number and Percentage Change in Sub-Saharan Africa and Sub-Regions between 1990 and 2021 based on GBD 2021 Report**

| Region | TB | | | DS-TB | | | MDR-TB | | | XDR-TB | | |
| --- | --- | --- | --- | --- | --- | --- | --- | --- | --- | --- | --- | --- |
|  | 1990 Number (95% UI) | 2021 Number (95% UI) | Prevalence % Change (1990–2021) | 1990 Number (95% UI) | 2021 Number (95% UI) | Prevalence % Change (1990–2021) | 1990 Number (95% UI) | 2021 Number (95% UI) | Prevalence % Change (1990–2021) | 2000 Number (95% UI) | 2021 Number (95% UI) | Prevalence % Change (2000–2021) |
| Sub-Saharan Africa | 178447050 (159832028, 198614426) | 257313631 (229669367, 289126065) | 44.20 (43.69, 45.57) | 3774699 (3379300, 4204942) | 4185953 (3673731, 4702706) | 10.90 (8.71, 11.84) | 7516 (4455, 12701) | 129234 (86814, 189169) | 1619.55 (1848.54, 1389.42) | 144 (102, 217) | 806 (541, 1183) | 459.23 (430.39, 445.88) |
| Western Sub-Saharan Africa | 71204482 (64028755, 79217042) | 85468308 (76100617, 96715000) | 20.03 (18.85, 22.09) | 1190451 (1065240, 1327147) | 1205994 (1059365, 1356705) | 1.31 (-0.55, 2.23) | 3228 (1688, 5842) | 37131 (18591, 74598) | 1050.31 (1001.42, 1176.99) | 49 (26, 93) | 232 (116, 465) | 374.23 (351.31, 400.94) |
| Eastern Sub-Saharan Africa | 66079629 (59119633, 73580551) | 102369772 (90904688, 115616669) | 54.92 (53.76, 57.13) | 1545359 (1373681, 1726499) | 1589184 (1386920, 1806833) | 2.84 (0.96, 4.65) | 1065 (474, 2418) | 55820 (33102, 89517) | 5140.13 (6881.96, 3602.40) | 58 (37, 90) | 348 (206, 558) | 500.11 (452.88, 517.21) |
| Central Sub-Saharan Africa | 20090142 (17880369, 22478854) | 40940521 (36256169, 46249255) | 103.78 (102.77, 105.75) | 547985 (485811, 614208) | 872971 (771333, 985171) | 59.31 (58.77, 60.40) | 1377 (349, 4845) | 19281 (5744, 51167) | 1300.31 (1543.84, 956.11) | 18 (6, 50) | 120 (36, 319) | 564.59 (535.44, 532.65) |
| Southern Sub-Saharan Africa | 21072797 (18867381, 23118634) | 28535030 (26083006, 31291828) | 35.41 (38.24, 35.35) | 490904 (429007, 552470) | 517803 (442419, 592599) | 5.48 (3.13, 7.26) | 1845 (388, 5433) | 17001 (7492, 35462) | 821.26 (1832.64, 552.73) | 19 (10, 43) | 106 (47, 221) | 452.58 (381.11, 415.46) |
| TB: Tuberculosis; DS-TB: Drug-susceptible tuberculosis; MDR-TB: Multidrug-resistant tuberculosis; | | | | | | | | | | | | |
| XDR-TB: Extensively drug-resistant tuberculosis. | | | | | | | | | | | | |
| Values represent number and percentage change (95% Uncertainty Interval) from 1990 to 2021 for TB, DS-TB, and MDR-TB, and from 2000 to 2021 for XDR-TB. NA indicates data not available. | | | | | | | | | | | | |

**Table S4: Tuberculosis DALYs (Disability-Adjusted Life Years) Number and Percentage Change in Sub-Saharan Africa and Sub-Regions between 1990 and 2021 based on GBD 2021 Report**

| Region | TB | | | DS-TB | | | MDR-TB | | | XDR-TB | | |
| --- | --- | --- | --- | --- | --- | --- | --- | --- | --- | --- | --- | --- |
|  | 1990 Number (95% UI) | 2021 Number (95% UI) | DALYs % Change (1990–2021) | 1990 Number (95% UI) | 2021 Number (95% UI) | DALYs % Change (1990–2021) | 1990 Number (95% UI) | 2021 Number (95% UI) | DALYs % Change (1990–2021) | 2000 Number (95% UI) | 2021 Number (95% UI) | DALYs % Change (2000–2021) |
| Sub-Saharan Africa | 22778559 (19434725, 26793114) | 17135993 (14014437, 20397541) | -24.77 (-27.89, -23.87) | 22689093 (19370535, 26671270) | 15975604 (13124185, 19216449) | -29.59 (-32.25, -27.95) | 89465 (33733, 195361) | 1144754 (506655, 2310353) | 1179.55 (1401.94, 1082.61) | 4529 (1847, 9642) | 15636 (6378, 31734) | 245.22 (245.23, 229.14) |
| Western Sub-Saharan Africa | 6133277 (4981552, 7398402) | 4898305 (3664376, 6300381) | -20.14 (-26.44, -14.84) | 6099569 (4946120, 7361672) | 4578733 (3413713, 5846721) | -24.93 (-30.98, -20.58) | 33708 (11336, 83401) | 315280 (110106, 722712) | 835.32 (871.30, 766.55) | 1360 (409, 3472) | 4292 (1327, 9882) | 215.63 (224.60, 184.60) |
| Eastern Sub-Saharan Africa | 11380778 (9648924, 13360358) | 7096061 (5775925, 8662344) | -37.65 (-40.14, -35.16) | 11360490 (9636735, 13309129) | 6546849 (5355243, 8073644) | -42.37 (-44.43, -39.34) | 20288 (6202, 60894) | 541805 (213875, 1115146) | 2570.62 (3348.43, 1731.30) | 2125 (887, 4520) | 7407 (2792, 15206) | 248.61 (214.69, 236.38) |
| Central Sub-Saharan Africa | 3679323 (2739289, 4590476) | 3374106 (2490284, 4750750) | -8.30 (-9.09, 3.49) | 3657273 (2722486, 4556481) | 3212971 (2368397, 4622318) | -12.15 (-13.01, 1.44) | 22050 (4045, 86035) | 158952 (41334, 539484) | 620.88 (921.79, 527.05) | 632 (142, 1923) | 2183 (509, 7665) | 245.35 (258.26, 298.70) |
| Southern Sub-Saharan Africa | 1585181 (1381146, 1903929) | 1767521 (1539616, 2072433) | 11.50 (11.47, 8.85) | 1571761 (1371899, 1885968) | 1637051 (1377570, 1944372) | 4.15 (0.41, 3.10) | 13420 (2327, 41971) | 128716 (47514, 287591) | 859.16 (1941.80, 585.22) | 413 (144, 1031) | 1754 (594, 4156) | 325.07 (313.81, 302.90) |
| TB: Tuberculosis; DS-TB: Drug-susceptible tuberculosis; MDR-TB: Multidrug-resistant tuberculosis; | | | | | | | | | | | | |
| XDR-TB: Extensively drug-resistant tuberculosis. | | | | | | | | | | | | |
| Values represent number and percentage change (95% Uncertainty Interval) from 1990 to 2021 for TB, DS-TB, and MDR-TB, and from 2000 to 2021 for XDR-TB. NA indicates data not available. | | | | | | | | | | | | |

**Table S5: Age-Standardized Tuberculosis Prevalence Rates and Percentage Change in Sub-Saharan Africa and Sub-Regions between 1990 and 2021 based on GBD 2021 Report**

| Region | TB | | | DS-TB | | | MDR-TB | | | XDR-TB | | |
| --- | --- | --- | --- | --- | --- | --- | --- | --- | --- | --- | --- | --- |
|  | 1990 ASR (95% UI) | 2021 ASR (95% UI) | ASPR % Change (1990–2021) | 1990 ASR (95% UI) | 2021 ASR (95% UI) | ASPR % Change (1990–2021) | 1990 ASR (95% UI) | 2021 ASR (95% UI) | ASPR % Change (1990–2021) | 2000 ASR (95% UI) | 2021 ASR (95% UI) | ASPR % Change (2000–2021) |
| Sub-Saharan Africa | 40175.02 (36833.79, 43800.53) | 26028.36 (23494.71, 28695.01) | -35.21 (-36.21, -34.49) | 1002.41 (902.60, 1108.88) | 498.09 (443.00, 555.88) | -50.31 (-50.92, -49.87) | 1.92 (1.13, 3.23) | 15.26 (10.15, 22.33) | 693.70 (794.15, 592.07) | 0.03 (0.02, 0.04) | 0.10 (0.06, 0.14) | 215.27 (191.80, 211.79) |
| Western Sub-Saharan Africa | 40618.93 (37176.86, 44211.89) | 20691.61 (18526.29, 23170.75) | -49.06 (-50.17, -47.59) | 809.67 (734.16, 889.49) | 352.86 (310.05, 397.87) | -56.42 (-57.77, -55.27) | 2.10 (1.12, 3.73) | 10.93 (5.28, 21.85) | 419.70 (371.97, 485.01) | 0.03 (0.01, 0.05) | 0.07 (0.03, 0.14) | 161.48 (139.18, 170.39) |
| Eastern Sub-Saharan Africa | 39224.45 (35950.54, 42617.07) | 27288.65 (24653.06, 30148.77) | -30.43 (-31.43, -29.26) | 1157.42 (1034.56, 1284.09) | 525.69 (464.09, 588.07) | -54.58 (-55.14, -54.20) | 0.81 (0.36, 1.87) | 18.26 (11.03, 30.10) | 2161.81 (2991.12, 1512.79) | 0.03 (0.02, 0.05) | 0.11 (0.07, 0.19) | 229.95 (207.88, 253.36) |
| Central Sub-Saharan Africa | 40574.80 (36933.84, 44302.99) | 34387.56 (31064.40, 37869.66) | -15.25 (-15.89, -14.52) | 1319.66 (1188.19, 1473.92) | 901.88 (799.93, 1010.18) | -31.66 (-32.68, -31.46) | 3.32 (0.87, 11.83) | 19.93 (5.76, 53.80) | 500.19 (564.89, 354.91) | 0.03 (0.01, 0.09) | 0.12 (0.04, 0.34) | 270.57 (253.58, 254.40) |
| Southern Sub-Saharan Africa | 41911.23 (38186.88, 45398.47) | 36089.62 (33130.82, 39353.28) | -13.89 (-13.24, -13.32) | 968.26 (861.63, 1077.82) | 653.57 (575.66, 732.97) | -32.50 (-33.19, -32.00) | 3.63 (0.77, 10.64) | 21.31 (9.41, 44.94) | 486.57 (1116.79, 322.55) | 0.03 (0.02, 0.07) | 0.13 (0.06, 0.28) | 316.40 (260.27, 289.55) |
| TB: Tuberculosis; DS-TB: Drug-susceptible tuberculosis; MDR-TB: Multidrug-resistant tuberculosis; | | | | | | | | | | | | |
| XDR-TB: Extensively drug-resistant tuberculosis. | | | | | | | | | | | | |
| ASMR: Age-standardized mortality rate; ASPR: Age-standardized prevalence rate; ASIR: Age-standardized incidence rate (per 100,000 population) (95% UI) for all measures. | | | | | | | | | | | | |
| Values represent age-standardized rates and percentage change (95% Uncertainty Interval) from 1990 to 2021 for TB, DS-TB, and MDR-TB, and from 2000 to 2021 for XDR-TB. NA indicates data not available. | | | | | | | | | | | | |

**Table S6: Age-Standardized Tuberculosis DALYs (Disability-Adjusted Life Years) Rates and Percentage Change in Sub-Saharan Africa and Sub-Regions between 1990 and 2021 based on GBD 2021 Report**

| Region | TB | | | DS-TB | | | MDR-TB | | | XDR-TB | | |
| --- | --- | --- | --- | --- | --- | --- | --- | --- | --- | --- | --- | --- |
|  | 1990 ASR (95% UI) | 2021 ASR (95% UI) | ASDR % Change (1990–2021) | 1990 ASR (95% UI) | 2021 ASR (95% UI) | ASDR % Change (1990–2021) | 1990 ASR (95% UI) | 2021 ASR (95% UI) | ASDR % Change (1990–2021) | 2000 ASR (95% UI) | 2021 ASR (95% UI) | ASDR % Change (2000–2021) |
| Sub-Saharan Africa | 5496.64 (4671.66, 6492.05) | 2146.64 (1816.86, 2516.99) | -60.95 (-61.11, -61.23) | 5475.26 (4656.21, 6462.65) | 2002.35 (1677.31, 2373.04) | -63.43 (-63.98, -63.28) | 21.37 (8.23, 47.28) | 142.34 (63.50, 286.63) | 565.95 (671.90, 506.20) | 0.89 (0.36, 1.92) | 1.95 (0.79, 3.94) | 118.86 (118.87, 104.74) |
| Western Sub-Saharan Africa | 3679.71 (3099.01, 4430.16) | 1401.60 (1096.23, 1729.10) | -61.91 (-64.63, -60.97) | 3659.31 (3085.52, 4413.14) | 1310.34 (1022.21, 1628.52) | -64.19 (-66.87, -63.10) | 20.41 (6.83, 50.66) | 90.03 (31.43, 197.30) | 341.21 (360.33, 289.43) | 0.64 (0.20, 1.63) | 1.23 (0.39, 2.75) | 92.68 (94.18, 68.18) |
| Eastern Sub-Saharan Africa | 7618.19 (6423.06, 9177.08) | 2538.08 (2096.61, 3066.18) | -66.68 (-67.36, -66.59) | 7605.05 (6399.50, 9120.52) | 2345.73 (1904.02, 2864.53) | -69.16 (-70.25, -68.59) | 13.14 (4.01, 38.68) | 189.74 (75.50, 390.11) | 1344.35 (1781.60, 908.43) | 1.19 (0.50, 2.55) | 2.60 (1.01, 5.36) | 117.71 (104.01, 109.80) |
| Central Sub-Saharan Africa | 7331.50 (5252.37, 9365.87) | 3530.20 (2569.58, 4965.79) | -51.85 (-51.08, -46.98) | 7287.68 (5230.19, 9297.31) | 3361.35 (2444.44, 4756.21) | -53.88 (-53.26, -48.84) | 43.82 (8.41, 163.91) | 166.56 (42.73, 566.43) | 280.09 (408.34, 245.57) | 0.98 (0.22, 3.01) | 2.29 (0.55, 7.85) | 133.37 (151.63, 160.28) |
| Southern Sub-Saharan Africa | 3517.78 (3053.72, 4217.58) | 2369.82 (2080.46, 2768.09) | -32.63 (-31.87, -34.37) | 3488.22 (3040.24, 4187.08) | 2195.17 (1851.87, 2601.57) | -37.07 (-39.09, -37.87) | 29.57 (5.23, 92.01) | 172.30 (63.88, 382.47) | 482.74 (1120.77, 315.67) | 0.78 (0.27, 1.96) | 2.35 (0.80, 5.57) | 202.10 (193.61, 184.37) |
| TB: Tuberculosis; DS-TB: Drug-susceptible tuberculosis; MDR-TB: Multidrug-resistant tuberculosis; | | | | | | | | | | | | |
| XDR-TB: Extensively drug-resistant tuberculosis. | | | | | | | | | | | | |
| ASMR: Age-standardized mortality rate; ASPR: Age-standardized prevalence rate; ASIR: Age-standardized incidence rate (per 100,000 population) (95% UI) for all measures. | | | | | | | | | | | | |
| Values represent age-standardized rates and percentage change (95% Uncertainty Interval) from 1990 to 2021 for TB, DS-TB, and MDR-TB, and from 2000 to 2021 for XDR-TB. NA indicates data not available. | | | | | | | | | | | | |

**Table S7: Age-Standardized Tuberculosis Prevalence Rates and Percentage Change in 22 Sub-Saharan African Countries between 1990 and 2021 based on GBD 2021 Report**

| Country | TB | | | DS-TB | | | MDR-TB | | | XDR-TB | | |
| --- | --- | --- | --- | --- | --- | --- | --- | --- | --- | --- | --- | --- |
|  | 1990 ASR (95% UI) | 2021 ASR (95% UI) | ASPR % Change (1990–2021) | 1990 ASR (95% UI) | 2021 ASR (95% UI) | ASPR % Change (1990–2021) | 1990 ASR (95% UI) | 2021 ASR (95% UI) | ASPR % Change (1990–2021) | 2000 ASR (95% UI) | 2021 ASR (95% UI) | ASPR % Change (2000–2021) |
| Angola | 41813.60 (37966.12, 45701.93) | 33560.49 (30113.56, 37367.88) | -19.74 (-20.68, -18.24) | 1246.06 (1120.89, 1384.73) | 752.17 (662.31, 851.49) | -39.64 (-40.91, -38.51) | 3.06 (0.31, 11.36) | 18.49 (2.87, 53.31) | 503.41 (832.66, 369.43) | 0.03 (0.00, 0.11) | 0.12 (0.02, 0.33) | 244.16 (301.89, 203.44) |
| Cameroon | 40306.61 (36510.70, 44218.20) | 19982.26 (17770.57, 22388.12) | -50.42 (-51.33, -49.37) | 749.45 (673.10, 829.45) | 311.57 (269.44, 357.08) | -58.43 (-59.97, -56.95) | 2.59 (0.22, 11.73) | 7.08 (1.19, 21.63) | 173.12 (449.69, 84.45) | 0.02 (0.00, 0.07) | 0.04 (0.01, 0.13) | 141.96 (231.55, 102.24) |
| Central African Republic | 43857.49 (40113.10, 47720.69) | 41421.80 (37676.24, 45369.04) | -5.55 (-6.07, -4.93) | 1552.61 (1402.07, 1712.61) | 1216.45 (1096.64, 1338.69) | -21.65 (-21.78, -21.83) | 4.00 (0.73, 14.26) | 16.38 (2.68, 54.30) | 309.41 (269.63, 280.83) | 0.03 (0.01, 0.07) | 0.10 (0.02, 0.34) | 230.44 (39.25, 400.92) |
| Chad | 43121.65 (39176.94, 46808.94) | 24500.54 (21873.79, 27662.14) | -43.18 (-44.17, -40.90) | 1017.10 (905.78, 1149.08) | 540.78 (470.99, 617.35) | -46.83 (-48.00, -46.27) | 3.28 (0.31, 14.87) | 13.54 (2.37, 38.72) | 313.39 (653.29, 160.38) | 0.03 (0.00, 0.09) | 0.08 (0.01, 0.24) | 199.36 (235.53, 156.97) |
| Côte d'Ivoire | 40655.62 (36857.76, 44588.82) | 19755.27 (17523.00, 22259.65) | -51.41 (-52.46, -50.08) | 858.11 (770.78, 956.06) | 351.32 (304.56, 404.08) | -59.06 (-60.49, -57.74) | 9.97 (2.47, 29.84) | 11.10 (2.12, 34.34) | 11.34 (-13.98, 15.06) | 0.05 (0.02, 0.10) | 0.07 (0.01, 0.21) | 39.74 (-41.20, 116.68) |
| Democratic Republic of the Congo | 40172.44 (36535.67, 43979.83) | 34691.00 (31217.69, 38350.69) | -13.64 (-14.56, -12.80) | 1358.52 (1221.15, 1520.46) | 975.75 (860.66, 1093.13) | -28.18 (-29.52, -28.11) | 3.43 (0.42, 15.22) | 21.60 (3.67, 66.92) | 530.03 (776.09, 339.77) | 0.03 (0.00, 0.13) | 0.13 (0.02, 0.42) | 288.19 (383.98, 232.90) |
| Ethiopia | 41360.57 (37468.51, 45459.47) | 35639.62 (32303.70, 39519.65) | -13.83 (-13.78, -13.07) | 1393.08 (1227.51, 1574.35) | 476.07 (413.22, 531.61) | -65.83 (-66.34, -66.23) | 0.83 (0.10, 3.47) | 15.64 (2.35, 50.47) | 1787.16 (2367.39, 1355.21) | 0.04 (0.01, 0.10) | 0.10 (0.01, 0.31) | 160.39 (22.46, 219.77) |
| Ghana | 43854.06 (39745.15, 47831.63) | 22525.78 (19921.52, 25336.68) | -48.63 (-49.88, -47.03) | 803.27 (727.66, 875.71) | 382.66 (328.76, 436.94) | -52.36 (-54.82, -50.10) | 2.92 (0.22, 11.98) | 9.42 (1.46, 31.29) | 222.49 (558.08, 161.08) | 0.02 (0.00, 0.08) | 0.06 (0.01, 0.20) | 200.57 (281.20, 154.02) |
| Guinea | 42781.86 (39075.58, 46744.57) | 22968.87 (20347.53, 25771.67) | -46.31 (-47.93, -44.87) | 773.38 (692.29, 866.42) | 414.32 (358.20, 473.98) | -46.43 (-48.26, -45.29) | 2.73 (0.23, 12.05) | 8.69 (1.61, 25.85) | 218.47 (604.35, 114.54) | 0.02 (0.00, 0.07) | 0.05 (0.01, 0.16) | 184.65 (269.97, 131.71) |
| Kenya | 31348.07 (28072.90, 34865.57) | 28523.82 (25413.84, 31653.18) | -9.01 (-9.47, -9.21) | 708.54 (611.36, 795.75) | 409.77 (357.65, 460.14) | -42.17 (-41.50, -42.18) | 0.22 (0.03, 0.89) | 6.95 (1.64, 15.71) | 3011.87 (6076.17, 1671.66) | 0.02 (0.00, 0.06) | 0.04 (0.01, 0.10) | 150.45 (289.78, 68.44) |
| Madagascar | 40968.43 (37172.27, 45097.85) | 24745.20 (21830.95, 27928.89) | -39.60 (-41.27, -38.07) | 1294.58 (1168.06, 1431.56) | 683.82 (592.93, 786.25) | -47.18 (-49.24, -45.08) | 0.52 (0.06, 1.84) | 17.59 (3.04, 56.82) | 3265.21 (4597.86, 2982.11) | 0.03 (0.01, 0.08) | 0.11 (0.02, 0.35) | 334.94 (206.17, 333.26) |
| Malawi | 32888.05 (29495.54, 36674.17) | 14191.59 (12656.34, 16181.68) | -56.85 (-57.09, -55.88) | 1250.53 (1087.83, 1462.37) | 508.18 (433.85, 591.33) | -59.36 (-60.12, -59.56) | 0.40 (0.05, 1.44) | 10.45 (2.12, 29.98) | 2538.17 (4212.12, 1987.48) | 0.02 (0.00, 0.08) | 0.07 (0.01, 0.19) | 171.01 (205.43, 140.90) |
| Mozambique | 42112.06 (38427.00, 46314.70) | 26177.92 (23370.51, 29380.95) | -37.84 (-39.18, -36.56) | 1213.00 (1073.15, 1392.85) | 739.71 (621.69, 849.24) | -39.02 (-42.07, -39.03) | 2.96 (0.50, 9.70) | 39.22 (10.65, 95.14) | 1222.88 (2013.33, 880.59) | 0.07 (0.04, 0.11) | 0.24 (0.07, 0.59) | 272.42 (71.54, 453.58) |
| Niger | 43786.36 (40062.22, 47684.88) | 23355.21 (20591.80, 26319.51) | -46.66 (-48.60, -44.81) | 1075.08 (975.30, 1184.93) | 504.82 (437.77, 575.88) | -53.04 (-55.11, -51.40) | 3.73 (0.33, 16.41) | 11.57 (1.94, 33.02) | 210.41 (495.06, 101.28) | 0.03 (0.00, 0.11) | 0.07 (0.01, 0.21) | 147.31 (231.02, 93.10) |
| Nigeria | 39375.16 (35916.60, 43040.27) | 19940.86 (17860.79, 22302.78) | -49.36 (-50.27, -48.18) | 750.62 (675.55, 829.29) | 307.40 (265.82, 347.91) | -59.05 (-60.65, -58.05) | 0.62 (0.06, 2.49) | 12.41 (3.28, 35.21) | 1915.06 (5135.68, 1312.44) | 0.03 (0.01, 0.07) | 0.08 (0.02, 0.22) | 181.79 (235.50, 194.74) |
| South Africa | 46979.30 (42773.02, 50732.69) | 44165.90 (40517.48, 48272.80) | -5.99 (-5.27, -4.85) | 1010.44 (896.06, 1125.69) | 723.64 (631.88, 812.82) | -28.38 (-29.48, -27.79) | 4.37 (0.74, 14.19) | 20.36 (5.71, 49.46) | 365.64 (667.48, 248.52) | 0.04 (0.02, 0.09) | 0.13 (0.04, 0.31) | 247.55 (110.40, 239.21) |
| South Sudan | 40650.84 (36876.93, 44537.58) | 24846.49 (22111.08, 27892.28) | -38.88 (-40.04, -37.37) | 1147.89 (1031.48, 1272.01) | 750.17 (643.61, 863.42) | -34.65 (-37.60, -32.12) | 0.93 (0.09, 4.21) | 30.79 (5.88, 90.96) | 3219.90 (6354.58, 2058.56) | 0.04 (0.01, 0.18) | 0.19 (0.04, 0.57) | 329.55 (519.54, 217.90) |
| Sudan | 34990.81 (32068.35, 38106.48) | 18911.72 (16594.74, 21759.30) | -45.95 (-48.25, -42.90) | 122.73 (109.52, 138.70) | 42.04 (36.22, 48.48) | -65.74 (-66.93, -65.04) | 0.22 (0.02, 1.02) | 0.86 (0.12, 3.43) | 285.40 (379.41, 237.08) | 0.03 (0.00, 0.06) | 0.03 (0.00, 0.12) | 13.77 (-14.44, 83.27) |
| Uganda | 51080.01 (47939.52, 53864.09) | 38469.98 (34968.35, 42339.48) | -24.69 (-27.06, -21.40) | 702.52 (638.81, 764.25) | 434.02 (385.46, 481.70) | -38.22 (-39.66, -36.97) | 0.27 (0.02, 1.13) | 14.55 (3.65, 39.21) | 5383.55 (14941.13, 3382.30) | 0.02 (0.00, 0.07) | 0.09 (0.02, 0.24) | 274.75 (423.21, 255.42) |
| United Republic of Tanzania | 31974.07 (28683.70, 35572.06) | 14544.06 (12932.58, 16475.91) | -54.51 (-54.91, -53.68) | 1012.74 (883.35, 1151.01) | 371.78 (317.33, 425.86) | -63.29 (-64.08, -63.00) | 0.44 (0.05, 1.85) | 9.43 (1.61, 33.71) | 2023.97 (3092.36, 1726.47) | 0.02 (0.00, 0.06) | 0.06 (0.01, 0.21) | 200.59 (124.85, 278.96) |
| Zambia | 41802.48 (37804.73, 45580.15) | 21571.77 (19032.44, 24150.18) | -48.40 (-49.66, -47.02) | 1098.98 (984.54, 1232.37) | 477.11 (396.89, 555.23) | -56.59 (-59.69, -54.95) | 1.65 (0.24, 5.54) | 16.78 (2.80, 51.71) | 917.01 (1075.14, 832.97) | 0.03 (0.02, 0.07) | 0.10 (0.02, 0.32) | 200.78 (1.09, 378.35) |
| Zimbabwe | 26161.61 (22905.30, 29643.23) | 13004.06 (11620.57, 14633.12) | -50.29 (-49.27, -50.64) | 766.68 (642.46, 896.93) | 432.24 (355.79, 518.52) | -43.62 (-44.62, -42.19) | 1.86 (0.16, 8.66) | 19.12 (2.97, 62.44) | 925.29 (1702.22, 621.34) | 0.02 (0.00, 0.06) | 0.12 (0.02, 0.39) | 587.80 (591.54, 574.17) |
| TB: Tuberculosis; DS-TB: Drug-susceptible tuberculosis; MDR-TB: Multidrug-resistant tuberculosis; | | | | | | | | | | | | |
| XDR-TB: Extensively drug-resistant tuberculosis. | | | | | | | | | | | | |
| ASMR: Age-standardized mortality rate; ASPR: Age-standardized prevalence rate; ASIR: Age-standardized incidence rate; ASDR: Age-standardized DALY rate (per 100,000 population) (95% UI). | | | | | | | | | | | | |
| Values represent age-standardized rates and percentage change (95% Uncertainty Interval) from 1990 to 2021 for TB, DS-TB, and MDR-TB, and from 2000 to 2021 for XDR-TB. NA indicates data not available. | | | | | | | | | | | | |

**Table S8: Age-Standardized Tuberculosis DALY Rates and Percentage Change in 22 Sub-Saharan African Countries between 1990 and 2021 based on GBD 2021 Report**

| Country | TB | | | DS-TB | | | MDR-TB | | | XDR-TB | | |
| --- | --- | --- | --- | --- | --- | --- | --- | --- | --- | --- | --- | --- |
|  | 1990 ASR (95% UI) | 2021 ASR (95% UI) | ASDR % Change (1990–2021) | 1990 ASR (95% UI) | 2021 ASR (95% UI) | ASDR % Change (1990–2021) | 1990 ASR (95% UI) | 2021 ASR (95% UI) | ASDR % Change (1990–2021) | 2000 ASR (95% UI) | 2021 ASR (95% UI) | ASDR % Change (2000–2021) |
| Angola | 8223.20 (5605.54, 10730.27) | 2708.31 (1908.37, 3593.80) | -67.06 (-65.96, -66.51) | 8174.76 (5574.15, 10696.56) | 2564.06 (1791.47, 3379.46) | -68.63 (-67.86, -68.41) | 48.45 (4.34, 197.98) | 142.31 (18.80, 444.07) | 193.73 (333.21, 124.30) | 1.05 (0.11, 3.75) | 1.95 (0.22, 6.44) | 86.00 (104.29, 72.02) |
| Cameroon | 2674.18 (2013.12, 3399.30) | 1065.65 (655.06, 1629.44) | -60.15 (-67.46, -52.07) | 2652.40 (1974.50, 3379.92) | 1013.12 (600.65, 1550.26) | -61.80 (-69.58, -54.13) | 21.78 (1.51, 110.96) | 51.83 (6.95, 167.70) | 137.93 (361.49, 51.13) | 0.40 (0.04, 1.47) | 0.70 (0.09, 2.52) | 73.10 (120.48, 70.93) |
| Central African Republic | 13041.90 (8838.75, 16401.12) | 9243.75 (6246.04, 12335.08) | -29.12 (-29.33, -24.79) | 12960.24 (8806.28, 16288.44) | 8957.36 (6012.69, 12141.22) | -30.89 (-31.72, -25.46) | 81.65 (10.78, 301.89) | 282.50 (34.15, 995.86) | 245.98 (216.75, 229.87) | 1.61 (0.39, 4.68) | 3.88 (0.44, 14.46) | 141.88 (14.93, 208.75) |
| Chad | 4332.25 (3272.23, 5698.93) | 2623.52 (1894.01, 3477.67) | -39.44 (-42.12, -38.98) | 4299.43 (3241.04, 5679.14) | 2482.02 (1766.76, 3267.31) | -42.27 (-45.49, -42.47) | 32.81 (2.76, 158.17) | 139.59 (22.22, 437.00) | 325.40 (705.18, 176.28) | 0.81 (0.12, 2.92) | 1.91 (0.26, 5.93) | 136.16 (125.19, 103.08) |
| Côte d'Ivoire | 3000.70 (2452.15, 3642.86) | 1161.73 (820.32, 1674.00) | -61.28 (-66.55, -54.05) | 2920.15 (2352.94, 3571.36) | 1082.73 (749.37, 1588.84) | -62.92 (-68.15, -55.51) | 80.55 (12.72, 265.87) | 77.95 (11.81, 256.88) | -3.23 (-7.23, -3.38) | 1.01 (0.33, 2.39) | 1.05 (0.15, 3.39) | 4.35 (-56.10, 41.89) |
| Democratic Republic of the Congo | 6870.05 (4863.59, 9376.13) | 3652.23 (2569.16, 5712.67) | -46.84 (-47.18, -39.07) | 6829.12 (4839.23, 9317.74) | 3472.76 (2423.36, 5440.75) | -49.15 (-49.92, -41.61) | 40.93 (3.85, 196.33) | 177.03 (25.77, 709.65) | 332.55 (569.54, 261.45) | 0.95 (0.11, 3.65) | 2.44 (0.33, 9.51) | 156.43 (203.43, 160.66) |
| Ethiopia | 11895.38 (9639.72, 13811.94) | 1833.15 (1559.79, 2167.08) | -84.59 (-83.82, -84.31) | 11877.82 (9624.49, 13805.82) | 1709.49 (1358.12, 2077.55) | -85.61 (-85.89, -84.95) | 17.56 (1.63, 71.80) | 122.00 (18.21, 396.12) | 594.85 (1019.00, 451.68) | 1.41 (0.34, 4.11) | 1.66 (0.22, 5.02) | 17.83 (-36.96, 22.10) |
| Ghana | 3392.64 (2709.85, 4378.28) | 1402.41 (1033.89, 1887.09) | -58.66 (-61.85, -56.90) | 3363.47 (2666.06, 4333.67) | 1325.59 (956.23, 1803.36) | -60.59 (-64.13, -58.39) | 29.18 (1.93, 130.77) | 75.79 (9.20, 267.31) | 159.77 (376.21, 104.42) | 0.40 (0.04, 1.72) | 1.03 (0.12, 4.07) | 156.57 (210.87, 136.59) |
| Guinea | 3237.33 (2708.95, 4055.38) | 1485.96 (1078.09, 2120.57) | -54.10 (-60.20, -47.71) | 3210.23 (2686.54, 3991.54) | 1416.92 (998.06, 2042.48) | -55.86 (-62.85, -48.83) | 27.09 (1.84, 119.86) | 68.11 (9.14, 251.93) | 151.40 (397.96, 110.18) | 0.44 (0.05, 1.57) | 0.93 (0.12, 3.55) | 109.68 (161.85, 126.66) |
| Kenya | 4298.82 (2578.32, 6594.73) | 2795.46 (1694.56, 3743.51) | -34.97 (-34.28, -43.23) | 4295.48 (2577.02, 6576.95) | 2688.64 (1619.20, 3595.03) | -37.41 (-37.17, -45.34) | 3.34 (0.27, 14.98) | 105.38 (18.24, 307.04) | 3056.32 (6586.08, 1950.08) | 0.60 (0.08, 2.21) | 1.44 (0.27, 4.15) | 138.68 (249.19, 87.98) |
| Madagascar | 5989.89 (4827.64, 7309.15) | 2634.97 (1926.85, 3619.46) | -56.01 (-60.09, -50.48) | 5984.06 (4824.39, 7304.27) | 2485.87 (1804.83, 3428.50) | -58.46 (-62.59, -53.06) | 5.83 (0.59, 22.72) | 147.08 (20.03, 517.80) | 2422.35 (3300.05, 2178.82) | 0.57 (0.10, 1.86) | 2.02 (0.26, 7.58) | 251.90 (152.55, 307.29) |
| Malawi | 6512.00 (4997.76, 8619.64) | 2492.65 (1668.33, 3595.49) | -61.72 (-66.62, -58.29) | 6506.92 (4993.66, 8618.94) | 2379.46 (1585.46, 3455.58) | -63.43 (-68.25, -59.91) | 5.08 (0.48, 20.91) | 111.65 (19.01, 375.94) | 2097.52 (3822.32, 1697.54) | 0.78 (0.10, 2.87) | 1.54 (0.23, 4.91) | 97.52 (118.88, 71.23) |
| Mozambique | 8183.21 (6287.15, 11034.32) | 4286.41 (3039.34, 5631.76) | -47.62 (-51.66, -48.96) | 8134.49 (6227.39, 10971.09) | 3816.65 (2586.58, 5213.50) | -53.08 (-58.46, -52.48) | 48.73 (6.24, 191.38) | 463.41 (106.92, 1241.77) | 851.07 (1612.24, 548.85) | 2.26 (0.84, 5.15) | 6.35 (1.39, 16.58) | 180.79 (66.42, 222.23) |
| Niger | 5502.52 (4191.50, 7610.41) | 1967.08 (1345.74, 3180.21) | -64.25 (-67.89, -58.21) | 5457.26 (4127.34, 7541.74) | 1867.57 (1249.67, 2983.73) | -65.78 (-69.72, -60.44) | 45.26 (3.06, 224.37) | 98.16 (12.62, 316.80) | 116.89 (312.77, 41.20) | 0.74 (0.06, 2.77) | 1.34 (0.15, 4.28) | 80.65 (138.94, 54.32) |
| Nigeria | 3939.47 (3061.77, 5098.15) | 1355.49 (954.41, 1769.29) | -65.59 (-68.83, -65.30) | 3931.72 (3056.60, 5084.28) | 1248.41 (858.89, 1625.45) | -68.25 (-71.90, -68.03) | 7.75 (0.64, 34.71) | 105.63 (22.36, 289.94) | 1262.79 (3385.66, 735.39) | 0.75 (0.12, 2.32) | 1.44 (0.29, 4.04) | 91.40 (137.29, 74.25) |
| South Africa | 3420.54 (2935.50, 4192.02) | 1855.04 (1661.27, 2115.30) | -45.77 (-43.41, -49.54) | 3386.48 (2914.69, 4131.05) | 1748.98 (1496.38, 2028.46) | -48.35 (-48.66, -50.90) | 34.05 (4.85, 117.41) | 104.66 (22.74, 281.55) | 207.34 (368.47, 139.80) | 0.80 (0.25, 2.29) | 1.41 (0.31, 4.02) | 75.73 (24.55, 76.04) |
| South Sudan | 5623.49 (3904.99, 8433.69) | 3784.77 (2655.35, 5727.73) | -32.70 (-32.00, -32.09) | 5612.78 (3902.56, 8428.63) | 3450.94 (2309.31, 5407.06) | -38.52 (-40.83, -35.85) | 10.70 (0.76, 53.41) | 329.33 (48.85, 1011.72) | 2977.08 (6367.48, 1794.12) | 1.03 (0.11, 3.96) | 4.50 (0.68, 14.57) | 339.27 (528.39, 268.24) |
| Sudan | 1002.84 (687.20, 1424.52) | 184.73 (116.85, 270.61) | -81.58 (-83.00, -81.00) | 998.41 (683.55, 1418.05) | 176.33 (109.35, 263.43) | -82.34 (-84.00, -81.42) | 4.43 (0.37, 20.10) | 7.82 (0.95, 28.83) | 76.48 (159.09, 43.44) | 0.82 (0.12, 2.48) | 0.58 (0.06, 2.29) | -29.09 (-47.05, -7.87) |
| Uganda | 4367.14 (3108.26, 6951.99) | 2042.35 (1548.72, 2782.52) | -53.23 (-50.17, -59.98) | 4363.15 (3107.13, 6950.39) | 1895.90 (1389.08, 2579.29) | -56.55 (-55.29, -62.89) | 3.98 (0.31, 20.13) | 144.46 (27.95, 440.87) | 3525.15 (9048.88, 2089.97) | 0.92 (0.14, 3.10) | 1.99 (0.38, 6.75) | 115.46 (172.10, 117.95) |
| United Republic of Tanzania | 4639.93 (3566.27, 6278.63) | 1603.05 (1138.33, 2168.38) | -65.45 (-68.08, -65.46) | 4635.01 (3563.91, 6273.05) | 1514.39 (1035.84, 2029.91) | -67.33 (-70.94, -67.64) | 4.93 (0.38, 23.60) | 87.46 (13.18, 317.05) | 1675.83 (3327.38, 1243.66) | 0.49 (0.09, 1.58) | 1.19 (0.18, 4.66) | 141.90 (105.82, 194.82) |
| Zambia | 6507.35 (5019.08, 7932.47) | 1176.36 (780.69, 1750.75) | -81.92 (-84.45, -77.93) | 6484.41 (5007.90, 7920.97) | 1092.88 (708.83, 1644.23) | -83.15 (-85.85, -79.24) | 22.94 (2.57, 80.74) | 82.36 (11.58, 270.07) | 259.06 (351.27, 234.50) | 1.45 (0.45, 3.58) | 1.11 (0.14, 3.70) | -23.37 (-68.63, 3.49) |
| Zimbabwe | 3304.69 (2717.70, 3891.45) | 4427.65 (2879.56, 5875.10) | 33.98 (5.96, 50.97) | 3285.64 (2710.03, 3864.85) | 4030.88 (2468.70, 5492.73) | 22.68 (-8.90, 42.12) | 19.05 (1.29, 86.23) | 391.37 (55.07, 1266.68) | 1953.95 (4178.93, 1369.00) | 0.56 (0.06, 2.03) | 5.41 (0.76, 17.75) | 868.18 (1140.76, 773.56) |
| TB: Tuberculosis; DS-TB: Drug-susceptible tuberculosis; MDR-TB: Multidrug-resistant tuberculosis; | | | | | | | | | | | | |
| XDR-TB: Extensively drug-resistant tuberculosis. | | | | | | | | | | | | |
| ASMR: Age-standardized mortality rate; ASPR: Age-standardized prevalence rate; ASIR: Age-standardized incidence rate; ASDR: Age-standardized DALY rate (per 100,000 population) (95% UI). | | | | | | | | | | | | |
| Values represent age-standardized rates and percentage change (95% Uncertainty Interval) from 1990 to 2021 for TB, DS-TB, and MDR-TB, and from 2000 to 2021 for XDR-TB. NA indicates data not available. | | | | | | | | | | | | |

**Table S9: Age-Standardized HIV/AIDS-Tuberculosis Incidence Rates and Percentage Change Between 1990 and 2021 in SSA and Subregions**

| Region | TB | | | HIV/AIDS-DS-TB | | | HIV/AIDS-MDR-TB | | | HIV/AIDS- XDR-TB | | |
| --- | --- | --- | --- | --- | --- | --- | --- | --- | --- | --- | --- | --- |
|  | 1990 ASR (95% UI) | 2021 ASR (95% UI) | ASIR % Change (1990–2021) | 1990 ASR (95% UI) | 2021 ASR (95% UI) | ASIR % Change (1990–2021) | 1990 ASR (95% UI) | 2021 ASR (95% UI) | ASIR % Change (1990–2021) | 2000 ASR (95% UI) | 2021 ASR (95% UI) | ASIR % Change (2000–2021) |
| Sub-Saharan Africa | 478.07 (434.72, 529.19) | 257.14 (229.14, 284.70) | -46.21 (-47.29, -46.20) | 112.11 (100.49, 124.63) | 92.04 (82.09, 103.37) | -17.90 (-18.31, -17.06) | 0.27 (0.15, 0.50) | 3.69 (2.35, 5.77) | 1247.21 (1451.54, 1060.95) | 0.03 (0.02, 0.04) | 0.03 (0.02, 0.05) | 19.24 (7.66, 18.83) |
| Western Sub-Saharan Africa | 363.60 (330.80, 399.00) | 177.75 (155.88, 200.88) | -51.11 (-52.88, -49.65) | 44.10 (39.12, 48.42) | 33.03 (28.92, 37.33) | -25.09 (-26.09, -22.91) | 0.23 (0.10, 0.49) | 1.34 (0.61, 2.78) | 485.75 (508.37, 462.25) | 0.01 (0.01, 0.02) | 0.01 (0.01, 0.02) | 5.94 (-6.77, 13.49) |
| Eastern Sub-Saharan Africa | 574.71 (515.67, 636.78) | 282.94 (250.78, 314.99) | -50.77 (-51.37, -50.53) | 161.07 (144.03, 179.34) | 88.11 (77.54, 101.08) | -45.30 (-46.16, -43.64) | 0.14 (0.06, 0.32) | 3.82 (2.16, 6.26) | 2637.66 (3251.24, 1879.39) | 0.03 (0.02, 0.05) | 0.03 (0.02, 0.05) | 4.86 (-2.60, 7.99) |
| Central Sub-Saharan Africa | 545.66 (491.34, 602.20) | 392.31 (352.37, 437.48) | -28.10 (-28.28, -27.35) | 80.50 (71.45, 89.53) | 42.35 (37.64, 47.42) | -47.38 (-47.31, -47.04) | 0.26 (0.06, 1.01) | 1.11 (0.41, 2.42) | 320.59 (628.87, 139.11) | 0.01 (0.00, 0.03) | 0.01 (0.00, 0.02) | -13.62 (-3.01, -31.34) |
| Southern Sub-Saharan Africa | 544.24 (490.50, 608.71) | 417.09 (370.35, 470.40) | -23.36 (-24.49, -22.72) | 215.90 (185.95, 252.25) | 415.95 (369.19, 468.18) | 92.66 (98.55, 85.60) | 0.86 (0.19, 2.67) | 16.40 (7.79, 35.65) | 1804.99 (4013.67, 1233.68) | 0.07 (0.03, 0.16) | 0.12 (0.06, 0.26) | 76.19 (73.50, 59.73) |
| TB: Tuberculosis; HIV/AIDS-DS-TB: HIV/AIDS - Drug-susceptible Tuberculosis; HIV/AIDS-MDR-TB: HIV/AIDS - Multidrug-resistant Tuberculosis; | | | | | | | | | | | | |
| HIV/AIDS-XDR-TB: HIV/AIDS - Extensively drug-resistant Tuberculosis. | | | | | | | | | | | | |
| ASMR: Age-standardized mortality rate; ASPR: Age-standardized prevalence rate; ASIR: Age-standardized incidence rate (per 100,000 population) (95% UI) for all measures. | | | | | | | | | | | | |
| Values represent age-standardized rates and percentage change (95% Uncertainty Interval) from 1990 to 2021 for TB, DS-TB, and MDR-TB, and from 2000 to 2021 for XDR-TB. NA indicates data not available. | | | | | | | | | | | | |

**Table S10: Age-Standardized HIV/AIDS-Tuberculosis Prevalence Rates and Percentage Change Between 1990 and 2021 in SSA and Subregions**

| Region | TB | | | HIV/AIDS-DS-TB | | | HIV/AIDS-MDR-TB | | | HIV/AIDS- XDR-TB | | |
| --- | --- | --- | --- | --- | --- | --- | --- | --- | --- | --- | --- | --- |
|  | 1990 ASR (95% UI) | 2021 ASR (95% UI) | ASPR % Change (1990–2021) | 1990 ASR (95% UI) | 2021 ASR (95% UI) | ASPR % Change (1990–2021) | 1990 ASR (95% UI) | 2021 ASR (95% UI) | ASPR % Change (1990–2021) | 2000 ASR (95% UI) | 2021 ASR (95% UI) | ASPR % Change (2000–2021) |
| Sub-Saharan Africa | 40175.02 (36833.79, 43800.53) | 26028.36 (23494.71, 28695.01) | -35.21 (-36.21, -34.49) | 223.38 (200.36, 247.20) | 165.21 (146.65, 185.01) | -26.04 (-26.81, -25.16) | 0.47 (0.25, 0.85) | 6.22 (4.03, 9.62) | 1238.31 (1498.30, 1034.97) | 0.02 (0.01, 0.03) | 0.04 (0.03, 0.06) | 109.26 (91.24, 112.93) |
| Western Sub-Saharan Africa | 40618.93 (37176.86, 44211.89) | 20691.61 (18526.29, 23170.75) | -49.06 (-50.17, -47.59) | 96.59 (84.88, 106.67) | 66.37 (57.78, 75.03) | -31.29 (-31.93, -29.66) | 0.47 (0.20, 1.03) | 2.47 (1.17, 4.88) | 423.90 (490.26, 375.22) | 0.01 (0.01, 0.02) | 0.02 (0.01, 0.03) | 55.81 (38.87, 64.04) |
| Eastern Sub-Saharan Africa | 39224.45 (35950.54, 42617.07) | 27288.65 (24653.06, 30148.77) | -30.43 (-31.43, -29.26) | 341.36 (302.01, 381.53) | 165.66 (144.49, 187.93) | -51.47 (-52.16, -50.74) | 0.25 (0.12, 0.52) | 6.70 (3.88, 11.16) | 2557.64 (3162.81, 2027.80) | 0.02 (0.01, 0.03) | 0.04 (0.02, 0.07) | 98.07 (74.76, 113.16) |
| Central Sub-Saharan Africa | 40574.80 (36933.84, 44302.99) | 34387.56 (31064.40, 37869.66) | -15.25 (-15.89, -14.52) | 189.19 (168.35, 214.27) | 93.62 (83.18, 104.70) | -50.52 (-50.59, -51.14) | 0.57 (0.12, 2.23) | 2.38 (0.89, 5.40) | 317.28 (661.72, 142.82) | 0.01 (0.00, 0.03) | 0.01 (0.01, 0.03) | 55.81 (71.64, 24.98) |
| Southern Sub-Saharan Africa | 41911.23 (38186.88, 45398.47) | 36089.62 (33130.82, 39353.28) | -13.89 (-13.24, -13.32) | 309.84 (267.72, 359.05) | 666.44 (589.51, 755.78) | 115.09 (120.19, 110.50) | 1.02 (0.24, 3.22) | 25.07 (11.59, 53.46) | 2369.53 (4760.00, 1559.43) | 0.05 (0.03, 0.11) | 0.16 (0.07, 0.33) | 215.51 (181.35, 201.33) |
| TB: Tuberculosis; HIV/AIDS-DS-TB: HIV/AIDS - Drug-susceptible Tuberculosis; HIV/AIDS-MDR-TB: HIV/AIDS - Multidrug-resistant Tuberculosis; | | | | | | | | | | | | |
| HIV/AIDS-XDR-TB: HIV/AIDS - Extensively drug-resistant Tuberculosis. | | | | | | | | | | | | |
| ASMR: Age-standardized mortality rate; ASPR: Age-standardized prevalence rate; ASIR: Age-standardized incidence rate (per 100,000 population) (95% UI) for all measures. | | | | | | | | | | | | |
| Values represent age-standardized rates and percentage change (95% Uncertainty Interval) from 1990 to 2021 for TB, DS-TB, and MDR-TB, and from 2000 to 2021 for XDR-TB. NA indicates data not available. | | | | | | | | | | | | |

**Table S11: Age-Standardized HIV/AIDS-Tuberculosis DALYs Rates and Percentage Change Between 1990 and 2021 in SSA and Subregions**

| Region | TB | | | HIV/AIDS-DS-TB | | | HIV/AIDS-MDR-TB | | | HIV/AIDS- XDR-TB | | |
| --- | --- | --- | --- | --- | --- | --- | --- | --- | --- | --- | --- | --- |
|  | 1990 ASR (95% UI) | 2021 ASR (95% UI) | ASDR % Change (1990–2021) | 1990 ASR (95% UI) | 2021 ASR (95% UI) | ASDR % Change (1990–2021) | 1990 ASR (95% UI) | 2021 ASR (95% UI) | ASDR % Change (1990–2021) | 2000 ASR (95% UI) | 2021 ASR (95% UI) | ASDR % Change (2000–2021) |
| Sub-Saharan Africa | 5496.64 (4671.66, 6492.05) | 2146.64 (1816.86, 2516.99) | -60.95 (-61.11, -61.23) | 1354.17 (977.09, 1884.38) | 902.54 (719.16, 1079.20) | -33.35 (-26.40, -42.73) | 5.87 (1.89, 13.79) | 78.33 (34.30, 148.40) | 1235.21 (1715.57, 975.99) | 0.99 (0.41, 2.08) | 1.07 (0.43, 2.11) | 8.30 (6.12, 1.55) |
| Western Sub-Saharan Africa | 3679.71 (3099.01, 4430.16) | 1401.60 (1096.23, 1729.10) | -61.91 (-64.63, -60.97) | 489.36 (371.25, 656.42) | 386.45 (264.71, 552.51) | -21.03 (-28.70, -15.83) | 6.70 (1.60, 18.37) | 31.97 (10.19, 73.71) | 377.06 (534.93, 301.34) | 0.44 (0.14, 1.01) | 0.44 (0.13, 0.98) | -1.05 (-6.85, -2.77) |
| Eastern Sub-Saharan Africa | 7618.19 (6423.06, 9177.08) | 2538.08 (2096.61, 3066.18) | -66.68 (-67.36, -66.59) | 2376.07 (1668.00, 3329.66) | 989.91 (761.62, 1210.95) | -58.34 (-54.34, -63.63) | 3.44 (0.85, 10.05) | 90.59 (37.58, 182.99) | 2532.50 (4314.08, 1720.98) | 1.24 (0.46, 2.59) | 1.24 (0.49, 2.60) | -0.28 (6.54, 0.44) |
| Central Sub-Saharan Africa | 7331.50 (5252.37, 9365.87) | 3530.20 (2569.58, 4965.79) | -51.85 (-51.08, -46.98) | 1063.94 (744.43, 1544.84) | 461.74 (346.48, 600.28) | -56.60 (-53.46, -61.14) | 7.54 (1.13, 28.56) | 26.72 (8.06, 64.04) | 254.49 (610.45, 124.25) | 0.44 (0.11, 1.33) | 0.36 (0.10, 1.01) | -17.68 (-7.28, -24.41) |
| Southern Sub-Saharan Africa | 3517.78 (3053.72, 4217.58) | 2369.82 (2080.46, 2768.09) | -32.63 (-31.87, -34.37) | 1332.59 (989.65, 1864.73) | 3400.80 (2869.16, 3753.34) | 155.20 (189.92, 101.28) | 9.58 (1.31, 35.94) | 304.42 (114.45, 658.52) | 3076.84 (8641.82, 1732.24) | 2.74 (0.89, 7.12) | 4.17 (1.46, 10.03) | 52.11 (64.22, 40.90) |
| TB: Tuberculosis; HIV/AIDS-DS-TB: HIV/AIDS - Drug-susceptible Tuberculosis; HIV/AIDS-MDR-TB: HIV/AIDS - Multidrug-resistant Tuberculosis; | | | | | | | | | | | | |
| HIV/AIDS-XDR-TB: HIV/AIDS - Extensively drug-resistant Tuberculosis. | | | | | | | | | | | | |
| ASMR: Age-standardized mortality rate; ASPR: Age-standardized prevalence rate; ASIR: Age-standardized incidence rate (per 100,000 population) (95% UI) for all measures. | | | | | | | | | | | | |
| Values represent age-standardized rates and percentage change (95% Uncertainty Interval) from 1990 to 2021 for TB, DS-TB, and MDR-TB, and from 2000 to 2021 for XDR-TB. NA indicates data not available. | | | | | | | | | | | | |

**Table S12: Comparison of Tuberculosis Control Policies in 22 Sub-Saharan African Countries, 2021**

| Country | TB Incidence Trend (2015–2021) | TB Deaths Trend (2015–2021) | Catastrophic Costs (% Households) (95% CI)  2015-2022 | Source |
| --- | --- | --- | --- | --- |
| Angola | -0.21 (-1.00, 1.35) | -9.92 (-13.67, -8.11) | NA |  |
| Cameroon | -11.45 (-11.96, -10.91) | -18.99 (-24.11, -13.25) | (46% (95%CI; 29-65%) | Household costs incurred when seeking and receiving paediatric tuberculosis services: a survey in Cameroon and Kenya |
| Central African Republic | 7.57 (7.54, 8.52) | -2.39 (-3.06, -0.60) | NA | NA |
| Chad | 6.38 (5.31, 6.40) | 0.87 (0.14, 4.02) | NA | NA |
| Côte d'Ivoire | -10.53 (-10.04, -9.94) | -22.74 (-26.39, -15.13) | NA | NA |
| Democratic Republic of Congo | 1.77 (0.51, 2.75) | -13.93 (-16.83, -11.24) | 56 (50–63) | WHO - National surveys of costs faced by tuberculosis patients and their households 2015-2021 |
| Ethiopia | 3.75 (3.94, 3.73) | -10.37 (-14.51, -5.37) | 66.1% | TB-related catastrophic costs and associated factors for patients in Ethiopia |
| Ghana | -8.41 (-9.85, -7.27) | -12.33 (-17.17, -6.78) | 64 (58–69) | WHO - National surveys of costs faced by tuberculosis patients and their households 2015-2021 |
| Guinea | -11.96 (-11.56, -11.47) | -22.96 (-28.41, -15.29) | NA | NA |
| Kenya | -12.60 (-13.11, -12.68) | -8.07 (-10.17, -2.65) | 27 (21–32)  53% (95%CI; 36-70%) | WHO - National surveys of costs faced by tuberculosis patients and their households 2015-2021; Household costs incurred when seeking and receiving paediatric tuberculosis services: a survey in Cameroon and Kenya |
| Madagascar | -4.12 (-5.49, -3.19) | -4.81 (-8.72, -6.87) | NA | NA |
| Malawi | 3.92 (2.80, 4.85) | -8.91 (-10.83, -2.84) | NA | NA |
| Mozambique | 8.85 (8.53, 10.18) | -15.76 (-21.31, -12.75) | NA | NA |
| Niger | 6.34 (7.68, 6.02) | -1.29 (-4.27, -0.41) | 72 (61-81) | WHO -Costs faced by people with TB and their households, social protection and human rights |
| Nigeria | -13.63 (-13.83, -13.25) | -18.24 (-25.80, -10.90) | 71 (66–75) | WHO - National surveys of costs faced by tuberculosis patients and their households 2015-2021 |
| South Africa | 2.72 (3.95, 3.46) | -11.90 (-16.26, -4.21) | 56 (49-63) | WHO - Costs faced by people with TB and their households, social protection and human rights |
| South Sudan | -5.55 (-5.10, -5.30) | 7.50 (4.42, 13.10) | NA | NA |
| Sudan | -7.06 (-7.53, -4.55) | -19.16 (-23.88, -16.65) | NA | NA |
| United Republic of Tanzania | 2.21 (-0.34, 3.74) | -12.46 (-16.09, -6.27) | 45 (37–53) | WHO - National surveys of costs faced by tuberculosis patients and their households 2015-2021 |
| Uganda | -14.21 (-13.88, -14.03) | -11.92 (-14.48, -5.59) | 53 (43–63) | WHO - National surveys of costs faced by tuberculosis patients and their households 2015-2021 |
| Zambia | 4.60 (4.39, 6.49) | -23.42 (-27.66, -16.18) | 58 (55-62) | WHO - Costs faced by people with TB and their households, social protection and human rights |
| Zimbabwe | -2.43 (0.06, -3.71) | -1.54 (-4.17, 3.75) | 80 (74–85) | WHO - National surveys of costs faced by tuberculosis patients and their households 2015-2021 |

Notes: Catastrophic Costs = percentage of TB-affected households facing catastrophic costs due to MTB treatment that is over 20% of annual income. Data sourced from WHO Global TB Reports (2015–2024), GBD 2021, and national reports. WHO; World Health Organization, NA; WHO Survey not yet conducted.
